# Causal Effect of the Triglyceride-Glucose Index and the Joint Exposure of Higher Glucose and Triglyceride With Extensive Cardio-Cerebrovascular Metabolic Outcomes in the UK Biobank: A Mendelian Randomization Study

**DOI:** 10.3389/fcvm.2020.583473

**Published:** 2021-01-22

**Authors:** Shucheng Si, Jiqing Li, Yunxia Li, Wenchao Li, Xiaolu Chen, Tonghui Yuan, Congcong Liu, Hongkai Li, Lei Hou, Bojie Wang, Fuzhong Xue

**Affiliations:** ^1^Department of Biostatistics, School of Public Health, Cheeloo College of Medicine, Shandong University, Jinan, China; ^2^Institute for Medical Dataology, Shandong University, Jinan, China; ^3^National Institute of Health Data Science of China, Jinan, China

**Keywords:** glucose, triglyceride, triglyceride-glucose index, cardio-cerebrovascular diseases, causal association

## Abstract

**Background:** The causal evidence of the triglyceride–glucose (TyG) index, as well as the joint exposure of higher glucose and triglyceride on the risk of cardio-cerebrovascular diseases (CVD), was lacking.

**Methods:** A comprehensive factorial Mendelian randomization (MR) was performed in the UK Biobank cohort involving 273,368 individuals with European ancestry to assess and quantify these effects. The factorial MR, MR-PRESSO, MR-Egger, meta-regression, sensitivity analysis, positive control, and external verification were utilized. Outcomes include major outcomes [overall CVD, ischemic heart diseases (IHD), and cerebrovascular diseases (CED)] and minor outcomes [angina pectoris (AP), acute myocardial infarction (AMI), chronic IHD (CIHD), heart failure (HF), hemorrhagic stroke (HS), and ischemic stroke (IS)].

**Results:** The TyG index significantly increased the risk of overall CVD [OR (95% CI): 1.20 (1.14–1.25)], IHD [OR (95% CI): 1.22 (1.15–1.29)], CED [OR (95% CI): 1.14 (1.05–1.23)], AP [OR (95% CI): 1.29 (1.20–1.39)], AMI [OR (95% CI): 1.27 (1.16–1.39)], CIHD [OR (95% CI): 1.21 (1.13–1.29)], and IS [OR (95% CI): 1.22 (1.06–1.40)]. Joint exposure to genetically higher GLU and TG was significantly associated with a higher risk of overall CVD [OR (95% CI): 1.17 (1.12–1.23)] and IHD [OR (95% CI): 1.22 (1.16–1.29)], but not with CED. The effect of GLU and TG was independent of each other genetically and presented dose–response effects in bivariate meta-regression analysis.

**Conclusions:** Lifelong genetic exposure to higher GLU and TG was jointly associated with higher cardiac metabolic risk while the TyG index additionally associated with several cerebrovascular diseases. The TyG index could serve as a more sensitive pre-diagnostic indicator for CVD while the joint GLU and TG could offer a quantitative risk for cardiac metabolic outcomes.

## Introduction

The lifelong genetic exposure to lower levels of low-density lipoprotein cholesterol (LDL-C) and systolic blood pressure was demonstrated and quantified to be associated with a lower risk of cardio-cerebrovascular diseases (CVD) (1). However, a considerable residual risk remains after achieving the recommended targets (2). Studies showed that the residual risk can be partly attributed to triglyceride-rich lipoproteins (3); moreover, insulin resistance (IR)-related glucose (GLU) and triglyceride (TG) were also thought to play an important role in the development of CVD (4).

To clarify these additional risk factors, researchers have committed to exploring the independent effect of circulating TG or GLU on some cardio-cerebrovascular metabolic outcomes (5–9). However, individuals exposed to both higher TG and GLU widely existed in the general population. The causal association of combined lifelong exposures with the risk of CVD has not been reliably quantified. Meanwhile, the triglyceride–glucose (TyG) index, a surrogate indicator of combined TG and GLU, which represented IR to some extent, has also been widely used to investigate the relationship with health-related outcomes. Still, the causal role of the TyG index on the risk of CVD and various subtypes has not been explored as well.

Collectively, the effectiveness of the TyG index, as well as the combined exposures in causality, was still unclear. Mendelian randomization (MR) could use genetic variation as instrumental variables to quantify the causality of lifelong exposure to certain factors; at the same time, factorial MR was conducive to estimate the effects of combined exposures. So, we aimed to evaluate these associations in a comprehensive design by using the UK Biobank individual dataset and comprehensive factorial MR design.

## Materials and Methods

### Study Population

**Figure 1:**
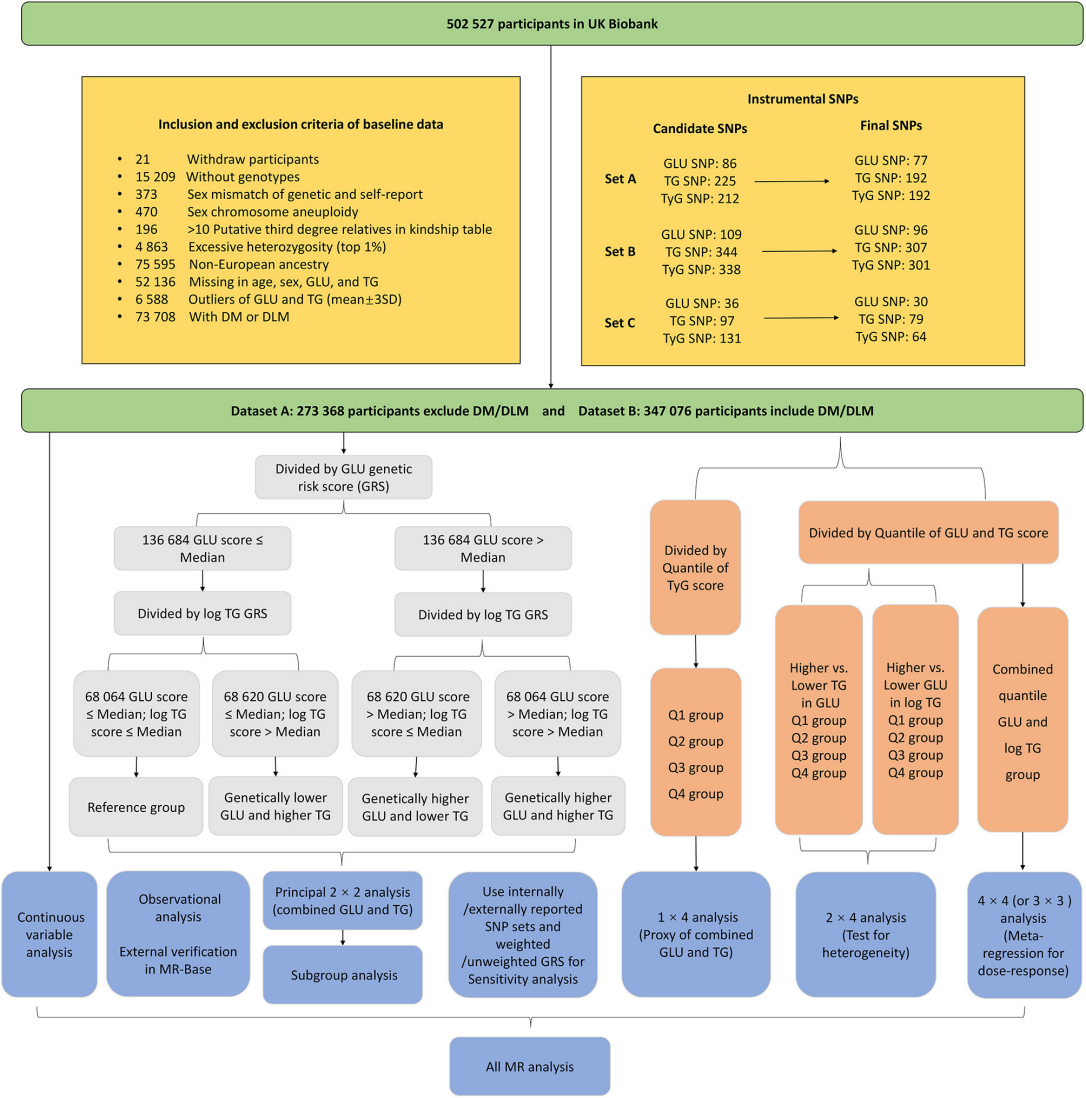
The inclusion and exclusion criteria of study participants and the factorial study design.

This study was performed based on the UK Biobank, a prospective population-based cohort comprising around 500,000 people aged 40–69 years who were recruited from 2006 to 2010 across the UK (10). The database contains genome-wide genotyping of all participants, blood biochemistry, and detailed health information from self-reported and electronic health records (11). This research performed quality control procedures on both UK Biobank participants and genotypes (see details in Figure 1). Here, we derived two datasets according to the above procedure: dataset A enrolled 273,368 participants after excluding individuals with diabetes mellitus (DM) and disorders of lipoprotein metabolism (DLM) to avoid the potential effect of hypoglycemic drugs or lipid-lowering drugs on non-fasting GLU and TG levels; dataset B enrolled 347,076 participants, including those with DM/DLM to assess the performance of generalization.

### Exposures and Outcomes

Non-fasting blood biochemistry was measured by the UK Biobank using standard hematological tests within 24 h of blood collection for all of the participants. Blood samples were taken at random and the time (hours) since the last meal and the fasting time were recorded when collecting. The random plasma TG and GLU in mmol/L were applied in this research. Since the original TG were non-normally distributed, we made a logarithmic transformation (log TG, we called TG in the following text). The non-fasting TyG index was calculated through the formula ln(triglycerides (mg/dl) × glucose (mg/dl)/2) (12).

The outcome in this research was defined by self-reported code, ICD-10, and ICD-9 codes from the health records of UK Biobank. It was divided into three categories: (1) major outcomes including the overall cardiovascular and cerebrovascular metabolic diseases (overall CVD), ischemic heart diseases (IHD), and cerebrovascular diseases (CED); (2) minor outcomes (CVD subtypes) including angina pectoris (AP), acute myocardial infarction (AMI), chronic ischemic heart disease (CIHD), heart failure (HF), hemorrhagic stroke (HS), and ischemic stroke (IS); (3) two related metabolic diseases including DM and DLM as positive controls. The detailed information about the definition of these diseases is shown in Supplementary Table 1.

### Instrumental Variables in MR

The instrumental variables were divided into three SNP sets (Figure 1). Set A and set B were acquired from genome-wide association analysis based on dataset A and dataset B, respectively, while set C was determined by external GWAS or MR studies. The process is as follows:

First, we performed a genome-wide association analysis in the UK Biobank cohort to acquire the single-nucleotide polymorphisms (SNPs) that are significantly associated with circulating GLU, TG, and TyG index. The imputation genotype file was used with the quality scores >0.3, the minor allele frequency >0.01, missing rate <0.05, and Hardy–Weinberg test *P* < 1 × 10^−5^. The effects of the instrumental SNPs were acquired at the genome-wide level of significance (*P* < 5 × 10^−8^) by using linear regression adjusted for age, sex, and the top 5 genetic principal components to control population stratification. This step was performed by Plink and Plink 2 software in the Linux system. To ensure independence, we further pruned these SNPs by linkage disequilibrium with each other by the *R*^2^ < 0.01 using the R function “clump_data” of the “TwoSampleMR” package. A total of 86, 225, and 212 candidate SNPs were selected for GLU, TG, and the TyG index, respectively (for dataset A). Then, we excluded the SNPs that were significantly associated with both TG and GLU and associated with non-lipid and non-glycemic components including SBP, DBP, and BMI to control the potential horizontal pleiotropic with the threshold *P* < 1 × 10^−7^. Besides, we also ruled out the SNP outliers (unknown pleiotropic SNPs) through the MR-PRESSO method (13). In total, 77, 192, and 192 IVs were selected for GLU, TG, and the TyG index, respectively (SNP set A). We repeated the above process to acquire 96, 307, and 301 IVs for GLU, TG, and the TyG index for participants including DM and DLM (SNP set B).

Then, to avoid the overestimated results, we additionally utilized external SNPs (set C) including 36 for GLU and 97 for TG, which was reported by other large genome-wide meta-analyses or MR studies (14–22). Since there were no reported SNPs for the TyG index, the combined IVs of TG and GLU were utilized. The effect values of these IVs for the TyG index were acquired by performing genome-wide association analysis in dataset A. Then, we performed the same procedures mentioned above (here, the MR-MRESSO has not been applied since some standard errors for SNPs were not available in some research and to keep enough IVs as well). Last, a total of 30 SNPs for GLU, 79 SNPs for TG, and 64 SNPs for the TyG index were determined. The detailed information of these instrumentals is shown in Supplementary Tables 2, 3.

Finally, we constructed the genetic risk score (GRS) for GLU, TG, and the TyG index by both weighted and unweighted method. The weighted GRS was calculated for each participant by summing the number of exposure-increasing alleles weighted by the β coefficients acquired from our genome-wide association analysis. The weighted GRS from externally reported SNPs were weighted using the corresponding β coefficients reported by other research, namely, the external effect size. For an SNP reported by more than one research, we used the β coefficients with the lowest *P*-values. The effect of a missing SNP was set to zero. Furthermore, an unweighted GRS was also built by only summing the number of effective alleles directly as a sensitivity analysis.

### Study Design

The main design of this research is presented in Figure 1. Each GRS for the exposure was discretized to two or four subgroups by the median or quantiles. To conduct the 2 × 2 factorial analysis, the GRS was used as an instrument of randomization to divide participants into four groups. First, participants were divided into two groups based on whether their genetic GLU score was equal to or lower than, or higher than the median value. Next, participants in either of these two groups were further divided into two more groups in the same way. Then, by quantile, the GRS for the TyG index was also discretized into four subgroups that are the same as the number of 2 × 2 combinations of GLU and TG score.

To conduct the 2 × 4 factorial analysis, the GRS for one of the exposures (GLU and TG) was dichotomized into a binary exposure by the median; meanwhile, another GRS was divided into four subgroups by quantiles (Q1–Q4). Firstly, we measured the independent effect of binary GLU (higher GRS vs. lower GRS) and test the heterogeneity by Q1–Q4 TG subgroups. Then, the effect of TG was measured in the same way. The 3 × 3 (or 4 × 4 in dataset B since it has a larger sample size) analysis required both quantiles of TG and GLU scores that will produce 9 (or 16) subgroups. We set the lowest combination (combined Q1 GLU and Q1 TG) as the reference and estimate the causal effects of the other 8 (or 15) TG and GLU combinations. The 3 × 3 (or 4 × 4) MR analysis could show the potential dose–response relationship and predict the causal effect from any combination of genetically determined TG and GLU. Additionally, we also take the GLU, TG, and the TyG index as the continuous variable (centralized to the same scale) to perform two-step MR analysis and made an external verification in the MR-Base platform (23).

### Statistical Analysis

Among the factorial subgroups, all continuous baseline variables were summarized by mean ± standard deviation (SD) and compared using one-way ANOVA analysis, while categorical variables were summarized by percentage and tested via Chi-squared statistics. The differences in GLU and TG between a specific subgroup and the reference group were calculated by the difference in the crude means, which will further be utilized in the meta-regression when scaling the causal effect to the suitable clinical unit. The differences in the risk of cardiovascular metabolic disease by factorial subgroups were measured by multivariable logistic regression adjusted for age, sex, BMI, smoke status, fasting time, and LDL. The two-step multivariable MR analysis also adjusted for the same covariates when regressing the continuous GRS and outcome. The causal effects were presented by odds ratios (ORs) and 95% confidence intervals (CIs). A *z*-test was used to assess interactions between pairs of subgroups, and the Cochran *Q*-test was used when comparing more than two subgroups.

To estimate the effect of combined 1-SD increased GLU (per 0.62 mmol/L) and TG (per 0.5 log mmol/L) on CVD and subtypes, the bivariate meta-regression was applied to regress the causal effects in the 3 × 3 (or 4 × 4) factorial analysis and then to predict the causal effect from any combination of GLU and TG. The external replication was performed in the MR-Base platform by two-sample MR (TSMR) analysis that matches our instrumental SNPs to the outcome from Coronary Artery Disease Genome-Wide Replication, Meta-analysis (CARDIOGRAM) plus the Coronary Artery Disease (C4D) genetics consortium (CARDIoGRAMplusC4D) (24, 25) and the MEGASTROKE Consortium (26), including coronary heart disease (CHD), myocardial infarction (MI), stroke, ischemic stroke (IS), cardioembolic stroke (CS), large artery stroke (LAS), and small vessel stroke (SVS). We performed external verification by both univariate and multivariate (BMI-adjusted) TSMR models.

Sensitivity analysis is defined as using both internally and externally reported SNP sets and both weighted and unweighted GRS in different datasets. All analysis based on dataset A with weighted GRS (SNP set A) was taken as the principal results. Meanwhile, we further repeated our analysis with unweighted GRS (SNP set A) in dataset A, weighted GRS (SNP set B) in dataset B, and weighted GRS (SNP set C) in dataset A with external-reported effect values. The capability of these GRS was assessed through the area under the curve (AUC) of the receiver operating characteristic (ROC) curve according to the predictive performance for the three phenotypes (binary variables) by 10-fold cross-validation. The variance explained by GRS was acquired from the regression between GRS and the corresponding phenotype. Then, we also made an observational analysis to repeat these processes by using the GLU, TG, and TyG index phenotypes directly. The DM and DML were taken as positive control outcomes to show the effectiveness of instrumental GRS of GLU and TG (only in dataset B). The remaining pleiotropy was assessed using the MR-Egger method by testing whether the intercept term was equal to zero. All analyses were two-tailed with a significance level of *P* < 0.05 and performed using R (Version 3.6.2), Plink, and Plink 2 software.

## Results

A total of 273,368 participants were included in this research. Supplementary Table 4 showed the baseline characteristics of the study population by the 2 × 2 subgroup of the TG and GLU subgroup. Among these participants, the non-lipid and non-glycemic characteristics including fasting time, sex, smoke, and drink presented no significant differences across 2 × 2 factorial subgroups. The IVs of GLU, TG, and the TyG index explained the variance by 2.3, 6.9, and 6.9%, respectively. Baseline characteristics of participants including DM and DLM are shown in Supplementary Table 5. The predictive performance of GRS for the three phenotypes shown in Supplementary Figure 1 presented approximately equivalent AUC across internal/external and weighted/unweighted GRS. MR-Egger test showed no significant horizontal pleiotropy for the IVs on any CVD (Supplementary Table 6, *P* for intercept term >0.05), except the GLU for HF and IS (*P* for intercept term = 0.041 and 0.037, respectively).

### Independent Associations of GLU and TG With CVD

**Figure 2:**
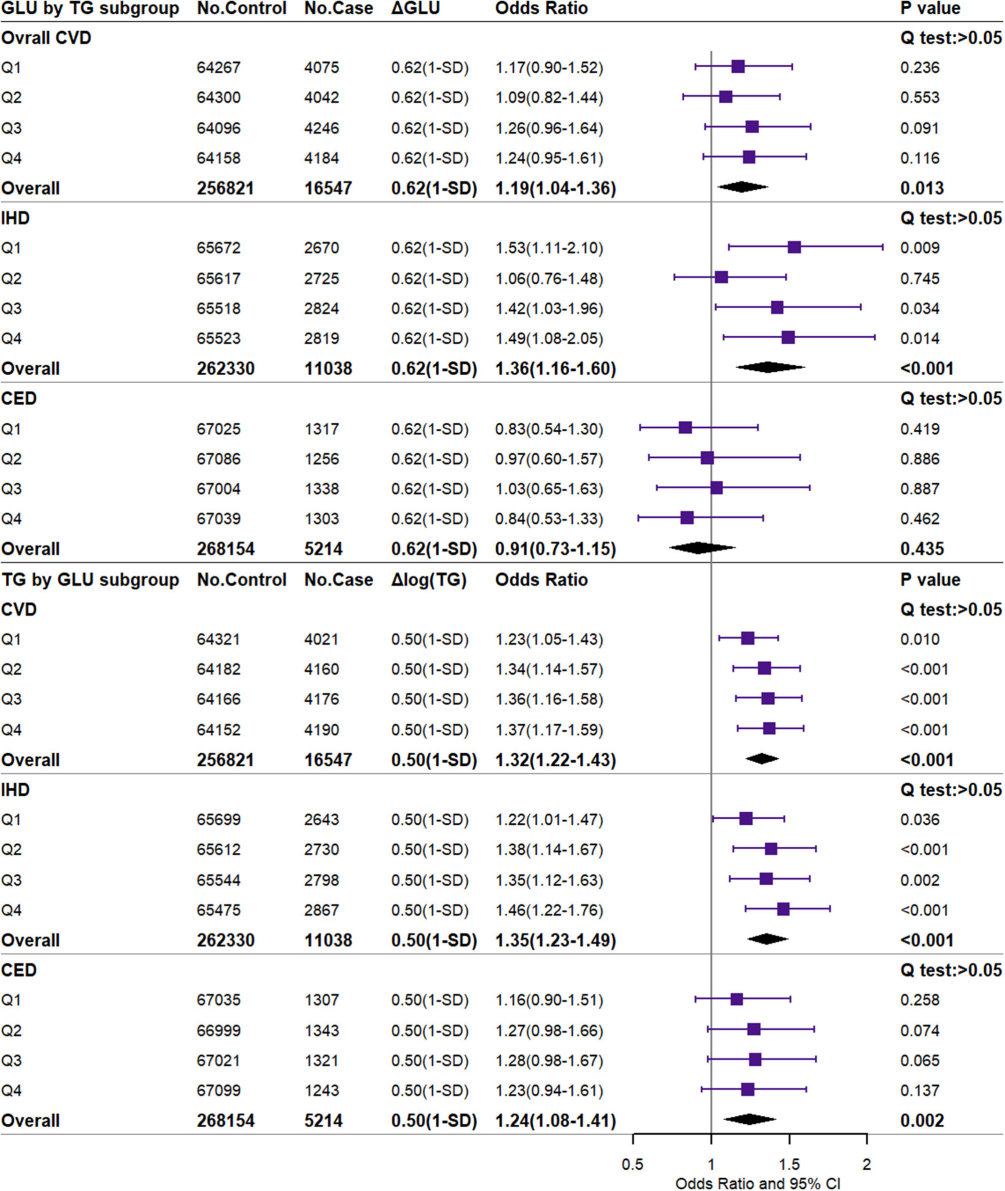
Independent associations of 1-SD higher glucose and triglyceride with the risk of major CVD. CVD, cardiovascular and cerebrovascular metabolic diseases; IHD, ischemic heart diseases; CED, cerebrovascular diseases; GLU, glucose; TG, triglyceride. ΔGLU and Δlog TG: the difference of observed glucose level between the higher GLU genetic score group or the higher TG genetic score group and the reference group. The difference value and corresponding OR (95% CI) were scaled to the 1-SD difference (0.62 mmol/L or 0.50 log mmol/L).

Figure 2 shows the independent causal effect of GLU and TG on CVD and the corresponding effects in TG and GLU subgroups from Q1 to Q4 (2 × 4 analysis). The genetically higher GLU (scaled to 1-SD difference) increased the risk of overall CVD and IHD by 19% [OR (95% CI): 1.19 (1.04–1.36)] and 36% [OR (95% CI): 1.36 (1.16–1.60)] and was not significantly associated with CED. The genetically higher TG (1-SD difference) increased the risk of overall CVD by 32%, the risk of IHD by 35%, and the risk of CED by 24% [OR (95% CI): 1.32 (1.22–1.43), 1.35 (1.23–1.49), and 1.24 (1.08–1.41), respectively]. Overall, no heterogeneity of effects was observed in the Q1–Q4 GLU/TG subgroups (*Q*-test: *p* > 0.05). For the minor CVD events, 1-SD higher GLU significantly increased the risk of AP, AMI, and CIHD by 34, 54, and 38%, respectively (Supplementary Figure 2). 1-SD higher TG significantly increased the risk of AP [OR (95% CI): 1.39 (1.23–1.58)], AMI [OR (95% CI): 1.64 (1.41–1.91)], CIHD [OR (95% CI): 1.35 (1.21–1.51)], HF [OR (95% CI): 1.36 (1.11–1.66)], and IS [OR (95% CI): 1.32 (1.05–1.67)] (Supplementary Figure 3). The non-scaled effects of GLU (0.150 mmol/L difference) and TG (0.210 logmmol/L difference) are shown in Supplementary Figures 4–6. Consistent results were reported by unweighted GRS for dataset A (Supplementary Figures 7–9), weighted GRS for dataset B (Supplementary Figures 10–12), and weighted GRS with external SNP set for dataset A (Supplementary Figures 13–15) in the sensitivity analysis. For dataset B, GLU and TG were also positively associated with DM and DLM. The observational results are shown in Supplementary Figures 16–18. Overall, the observational GLU presented an insignificant or even negative association with CVD or several subtypes, while TG is prone to the positive association.

### Associations of Jointly Higher GLU and TG With CVD

**Figure 3:**
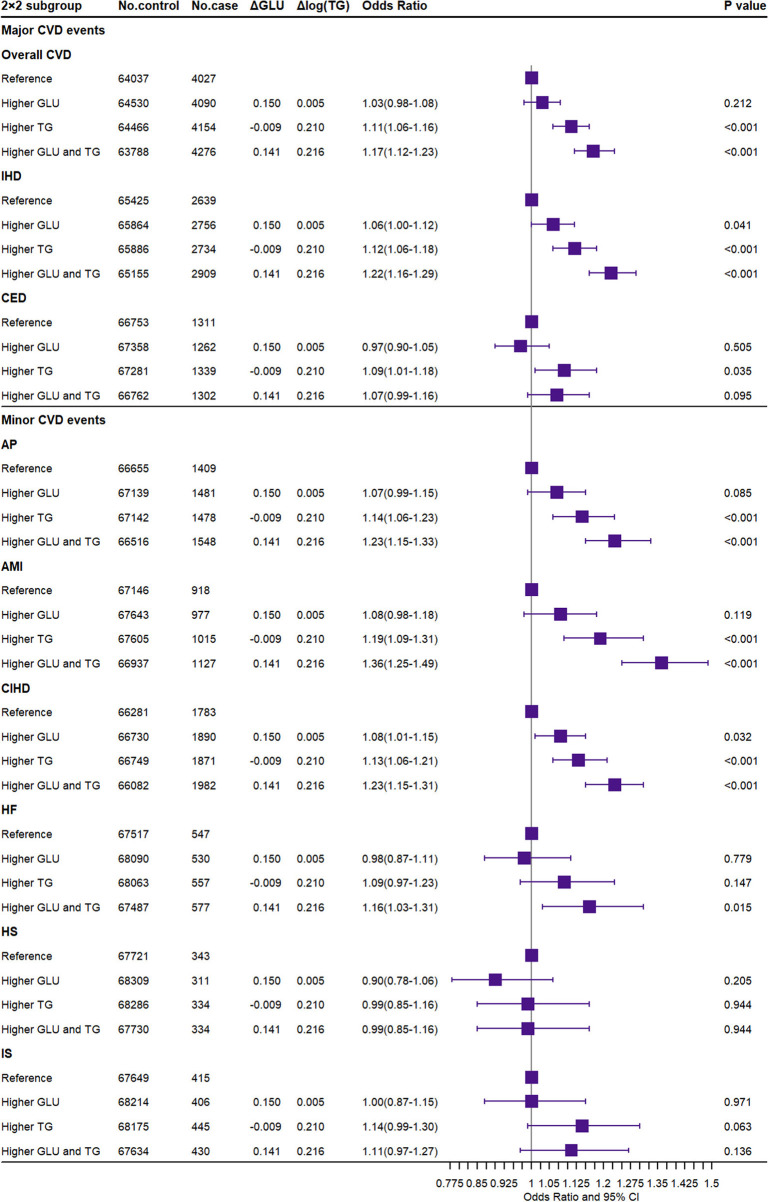
Association of joint exposure to higher glucose and triglyceride with the risk of CVD. CVD, cardiovascular and cerebrovascular metabolic diseases; IHD, ischemic heart diseases; CED, cerebrovascular diseases; AP, angina pectoris; AMI, acute myocardial infarction; CIHD, chronic ischemic heart disease; HF, heart failure; HS, hemorrhagic stroke; IS, ischemic stroke; GLU, glucose; TG, triglyceride. ΔGLU and Δlog TG: the difference of observed glucose level and log converted TG level between the higher GLU/TG genetic score group and the reference group.

As shown in Figure 3, the participants with GLU scores more than the median have 0.150 mmol/L higher GLU, and the OR was 1.03 (95% CI, 0.98–1.08, *P* = 0.212) for overall CVD compared to the reference. Meanwhile, the subgroup with TG scores more than the median has 0.210-unit log TG and an OR of 1.11 (95% CI, 1.06–1.16, *P* < 0.001). Participants in the group with both higher GLU and TG scores had an OR of 1.17 (95% CI, 1.12–1.23; *P* < 0.001) for overall CVD. In addition, the combined higher GLU and TG were strongly associated with an increased risk of IHD [OR (95% CI), 1.22 (1.16–1.29)] but insignificant with CED. When scaled by meta-regression, combined exposure to 1-SD higher GLU and TG was associated with 1.61-fold risk of overall CVD [OR (95% CI): 1.61 (1.44–1.80)], 1.80-fold risk of IHD [OR (95% CI): 1.80 (1.68–1.94)], and 1.39-fold risk of CED [OR (95% CI): 1.39 (1.06–1.83)] (Supplementary Figure 19). The magnitude of the association in the combined exposures was approximately equivalent to the log-additive associations with the risk of major CVD in the groups with higher GLU and TG, respectively.

For minor CVD events, the combined GLU and TG also significantly increased the risk of AP, AMI, CIHD, and HF [OR (95% CI): 1.23 (1.15–1.33), 1.36 (1.25–1.49), 1.23 (1.15–1.31), and 1.16 (1.03–1.31), respectively]. In summary, the combined GLU and TG were only significantly associated with cardiovascular metabolic outcomes but not with cerebrovascular metabolic outcomes. Consistent results were reported by both unweighted GRS for dataset A (Supplementary Figure 20), weighted GRS for dataset B (Supplementary Figure 21), and weighted external IV SNP set for dataset A (Supplementary Figure 22) in the sensitivity analysis. The observational results are shown in Supplementary Figure 23.

### Associations of the TyG Index With CVD

**Figure 4:**
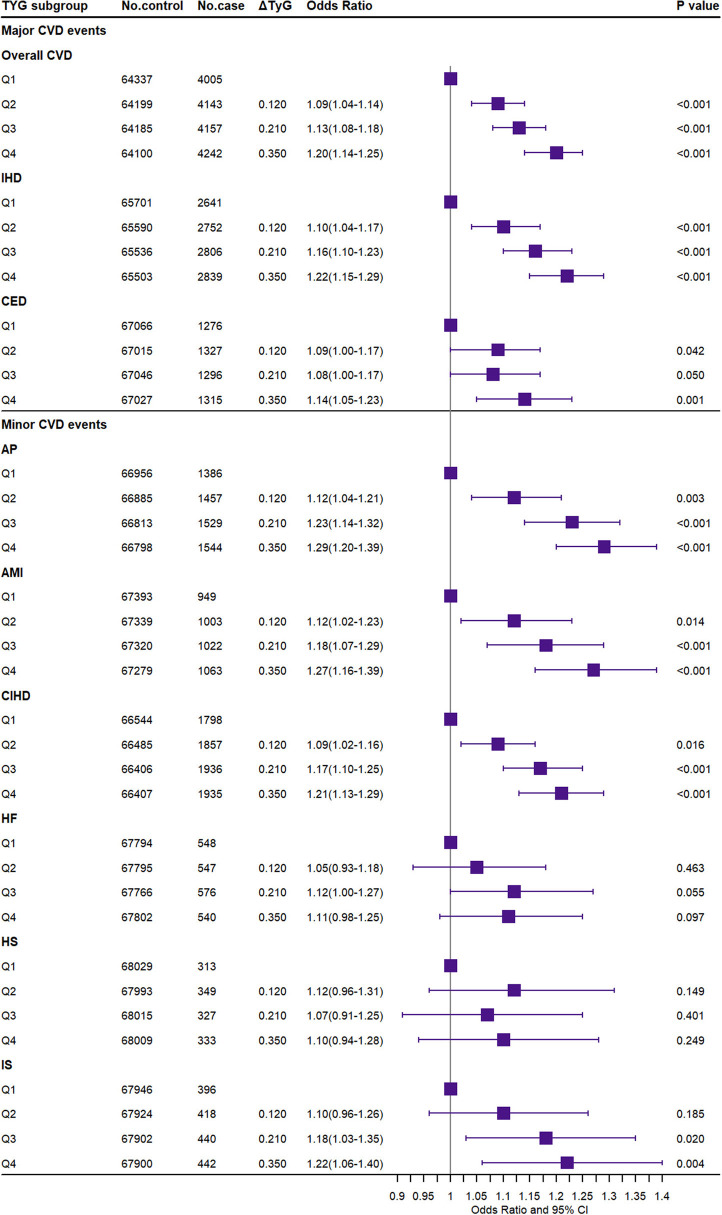
Association of the triglyceride–glucose index with the risk of CVD. CVD, cardiovascular and cerebrovascular metabolic diseases; IHD, ischemic heart diseases; CED, cerebrovascular diseases; AP, angina pectoris; AMI, acute myocardial infarction; CIHD, chronic ischemic heart disease; HF, heart failure; HS, hemorrhagic stroke; IS, ischemic stroke. ΔTyG: the difference of the observed triglyceride–glucose index level between the higher TyG index genetic score group (Q2–Q4) and the reference group (Q1).

Figure 4 presented the association between the TyG index and CVD events. Compared to the Q1 group, the highest TyG index (Q4) significantly increased the risk of major CVD including overall CVD [OR (95% CI): 1.20 (1.14–1.25)], IHD [OR (95% CI): 1.22 (1.15–1.29)], CED [OR (95% CI): 1.14 (1.05–1.23)], and minor CVD events including AP [OR (95% CI): 1.29 (1.20–1.39)], AMI [OR (95% CI): 1.27 (1.16–1.39)], CIHD [OR (95% CI): 1.21 (1.13–1.29)], and IS [OR (95% CI): 1.22 (1.06–1.40)]. In the sensitivity analysis, the above results were replicated. The weighted GRS in dataset B and the weighted external GRS additionally showed a significant association with HF, while the observational TyG index was not significant for IS (Supplementary Figures 24–27). The TyG index also significantly increased the risk of DM and DLM [OR (95% CI): 1.42 (1.36–1.48) and 1.78 (1.74–1.83)].

### Association of Dose–Response

**Figure 5:**
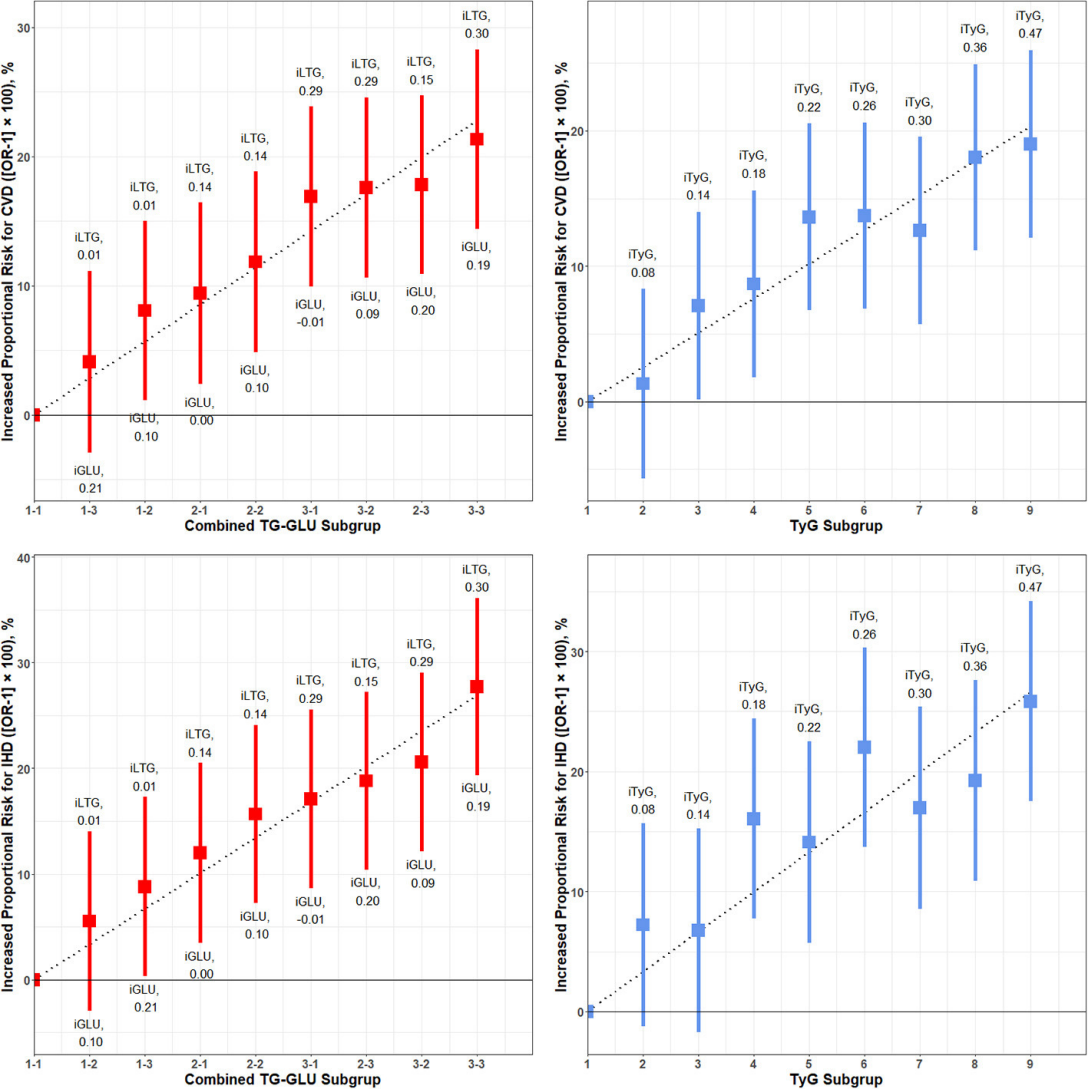
Dose–response associations of meta-regression for combined glucose and triglyceride on the risk of CVD and IHD. CVD, cardiovascular and cerebrovascular metabolic diseases; IHD, ischemic heart diseases; iLTG, incremental log TG; iGLU, incremental GLU, iTyG, incremental TyG index. The combined TG and GLU subgroup linked the Q1–Q4 TG group and Q–Q4 GLU group. The 1-1 subgroup was set as the reference, and the incremental TG and GLU was the crude mean difference compared to the reference. The risk of CVD and IHD for each subgroup relative to the reference group is plotted and expressed as an increased proportional risk. The dashed line is the regression line of increased proportional risk.

Figure 5 shows the results of meta-regression by 3 × 3 combined exposure to GLU and TG and the effect of the independent TyG index in the equal nine subgroups for the increased proportional risk for overall CVD and IHD, respectively. Both combined GLU/TG and the TyG index presented a significant dose–response relationship for CVD and IHD (*P* < 0.001 for meta-regression model). For overall CVD, the beta coefficients of GLU and TG in meta-regression was 0.32 (*P* = 0.01) and 0.56 (*P* < 0.001), and the beta coefficient for the TyG index was 0.46 (*P* < 0.001). For IHD, the beta coefficients of GLU and TG in meta-regression were 0.44 (*P* < 0.001) and 0.64 (*P* < 0.001), and the beta coefficient for the TyG index was 0.61 (*P* < 0.001). The result of TG and GLU was largely consistent with the 2 × 2 analysis. Sensitivity analysis in Supplementary Figures 28–31 further reproduces the above results.

### Subgroup Analysis, Continuous Exposures, and External Verification

The subgroup analysis for overall CVD is showed in Supplementary Figure 32. Among the subgroup of sex, age, BMI, fasting time, HDL-C, HbA1c, SBP, and smoke status, we found few interactions for the combined exposure to GLU and TG and the TyG index. The combined exposures and the TyG index presented strong interactions in the LDL-C subgroup (*P* < 0.001), so it was viewed as a potential covariate to be adjusted. Similar results for other CVD subtypes are also shown in Supplementary Tables 7, 8. Supplementary Figure 33 shows the effect of 1-SD changed (continuous variable) GLU, TG, and the TyG index on any CVD. The three exposures significantly increased the risk of CVD, IHD, AP, AMI, and CIHD, which is consistent with the factorial analysis. The TG and TyG index additionally predicted an increased risk of CED and IS while the TyG index also increased the risk of HF. Supplementary Figures 34, 35 show the results of external verification with univariate and multivariate TSMR analysis adjusted for BMI. These results were still consistent with the previous reports that show that GLU, TG, and TyG significantly increased the risk of cardiovascular metabolic diseases (including coronary heart disease and myocardial infarction) but not robust for cerebrovascular metabolic diseases. Also, TSMR showed that GLU is significantly associated with IS while TG and the TyG index are positively associated with LAS. The TyG index is additionally associated with SVS. In multivariable MR, the effect values only slightly changed, although a few were insignificant.

## Discussion

In this study, we investigated the causal association of the TyG index and the independent and combined association of GLU and TG with major and minor cardiovascular and cerebrovascular metabolic outcomes. The effect of GLU and TG was independent to each other at the genetic level. The combined exposures and TyG index predicted similar, robust, and a significantly increased risk for cardiovascular metabolic outcomes, while the TyG index was also significant for cerebrovascular metabolic outcomes.

For the independent TG and GLU, several studies have provided evidence that genetically elevated triglycerides are causally associated with an increased risk of CVD. Do et al. reported that the genetically determined elevated triglyceride levels were strongly associated with IHD (27). Our findings further confirmed this evidence and extended the scope of the cardiovascular metabolic diseases to more subtypes including the AP, AMI, CIHD, and HF. Besides, we proved that the effect of observational and genetically elevated TG levels was independent of the GLU with each other. Our research also reported some positive results of TG on cerebrovascular metabolic diseases like CED and IS and negative results of TG on HS. These findings were consistent with some observational studies that show that TG was related to a higher risk of stroke (28, 29).

The non-fasting GLU independently predicted the increased risk of IHD, AP, AMI, and CIHD, but not significant for CED, HF, HS, and IS. MR studies have also confirmed that glucose levels are prospectively associated with a high risk of IHD and MI (7, 8). The association between GLU and stroke was still controversial. Lee G et al. reported that increased fasting glucose in the non-diabetic population is associated with risks of MI and stroke in a retrospective cohort, but the effect for MI was larger than that for stroke (30). However, MR research further supports our results that genetically predicted fasting glucose and fasting insulin levels and BMI were not statistically significantly associated with any ischemic stroke subtype (9). Observational results show that glucose tends to have a protective effect on cardiovascular and cerebrovascular diseases and is not significant. There are some differences between these results and the abovementioned studies, indicating that observational results are more likely to be affected by confounders and that it will be difficult to obtain robust conclusions.

For the combined GLU and TG, no previous research has been conducted to quantify the CVD risk associated with the combined high TG and GLU. Similar research performed by Brian A et al. reported the combined exposure to lower LDL-C and SBP with the lifetime risk of CVD. Though the exposures were different, we found similar conclusions that GLU and TG also satisfy an independent, additive, and dose–response relationship for CVD and subtypes, but not for CED and its subtypes.

Our research further explored the causal role of the TyG index in the above diseases. This indicator has previously been widely used in observational studies and has reported positive associations with cardiovascular outcomes, coronary artery disease, IS, and cancer (12, 31–35), but no available causal evidence. The results from MR confirmed the effectiveness and high sensitivity of predicting values for the TyG index that successfully showed an increased risk of overall CVD, IHD, CED, AP, AMI, CIHD, IS, DM, and DLM when compared Q4 to Q1. These conclusions were largely the same as the combined GLU and TG but additionally predicted the risk of CED and IS. Apart from this, we found consistent conclusions from the observational TyG index, which means an ideal application in clinical practice even when lacking genetic information.

Finally, the above finding could also be interpreted and applied in suitable settings. First, the metabolic changes induced by high GLU, together with increased IR and free fatty acids, accelerate the atherosclerotic process through increased oxidative stress in arterial endothelial cells and the formation of advanced glycation end products, collectively resulting in vasoconstriction, inflammation, and thrombosis (7, 36). The potential mechanism for TG could also result in the CVD in similar ways since some studies demonstrated that elevated triglyceride-rich lipoproteins produce lipolytic products such as oxidized FFAs, which induce production of cytokines, interleukins, and proatherogenic adhesion molecules that may generate local inflammation in the arterial wall (37). Second, our research supported an independent effect of higher TG and GLU. Based on the independence, the increased linear proportional risk could be identified from any combination of GLU and TG via the meta-regression or log-additive values of independent GLU and TG. By providing a quantitatively rigorous method to estimate potential differences in cardiovascular risk that might be achieved with various public health strategies. Third, our research also confirmed the effectiveness of the TyG index in causality. The TyG index not only replicated the result of independent GLU and TG but also additionally predicted an increased risk of CED and IS. Besides, the TyG index also showed a linear dose–response relationship with the target outcomes that generate a similar predictive performance as combined GLU and TG. As a result, the TyG index could be utilized as a novel indicator in healthcare practice. Since the TyG index has a different scale from the combined GLU and TG, it seems implausible to compare the absolute values with each other. So, the TyG index could be used as conventional pre-diagnostic indicators while the combined GLU and TG will offer a quantitative risk for target outcome.

The strength of this research is reflected in the following aspects. To start with, the advantage of MR studies over observational studies is that the genetic-dependent exposures will suffer less confounding, reverse causation, and capture a lifelong effect. Then, the independent and combined effect of GLU and TG was measured and quantified for the first time by comprehensive design. Also, we explored the causal effect of TyG on CVD for the first time. Of course, some limitations should also be pointed out. The precision of results in factorial MR was largely dependent on the strength of instrumental SNPs; a more powerful set of IV should be further applied in future research under the control of potential pleiotropy. Second, factorial MR is not applicable for multivariable analysis, but we adjusted some potential covariates when regressing the GRS and outcome to control the remnant of confounding. Then, the fasting GLU and TG were not available in the UK Biobank, but it has been reported that non-fasting measures were superior predictors (38) and our results will not be influenced by the fasting time in subgroup analysis. Finally, it has been reported that the UK Biobank participants were less likely to be obese, to smoke, to drink, and to have fewer self-reported health conditions that are not representative of the sampling population, which suggested a “healthy volunteer” selection bias (39). Though it also mentioned that a valid assessment of exposure–disease relationships does not require participants to be representative of the population at large, more studies are needed to further confirm our findings.

## Conclusions

Lifelong genetic exposure to higher triglyceride and glucose was associated with higher overall CVD and cardiac metabolic risk. The GLU and TG showed an independent, additive, and dose–response effect on CVD. The TyG index could be used as a sensitive pre-diagnostic indicator while the combined GLU and TG will offer a quantitative risk for target outcome.

## Data Availability Statement

This research has been conducted using the UK Biobank Resource (Application ID: 51470). Researchers could acquire this data by submitting an application to the UK Biobank (https://www.ukbiobank.ac.uk/) through the UK Biobank Access Management System (https://bbams.ndph.ox.ac.uk/ams/).

## Ethics Statement

The UK Biobank has ethical approval from the Northwest Multi-Center Research Ethics Committee, and all participants provided written informed consent.

## Author Contributions

SS and FX conceived the study. SS did the statistical analyses and drafted the initial manuscript. All authors participated in the interpretation of the results, and edited and reviewed the manuscript.

## Conflict of Interest

The authors declare that the research was conducted in the absence of any commercial or financial relationships that could be construed as a potential conflict of interest.
