# Supplementary material for: Causal Effect of the Triglyceride-Glucose Index and the Joint Exposure of Higher Glucose and Triglyceride With Extensive Cardio-Cerebrovascular Metabolic Outcomes in the UK Biobank: A Mendelian Randomization Study
Source: Front Cardiovasc Med. 2021 Jan 22;7:583473. doi: 10.3389/fcvm.2020.583473 (PMC7863795; doi:10.3389/fcvm.2020.583473)
Supplement: Supplementary file 1 [file Data_Sheet_1.pdf]

Supplement Tables and Figures

Causal Effect of the Triglyceride-Glucose Index and the Joint Exposure of Higher Glucose and Triglyceride With Extensive Cardio-Cerebrovascular Metabolic Outcomes in the UK Biobank: A Mendelian Randomization Study

Shucheng Si[1,2,3] (0000-0001-9858-7089), Jiqing Li[1], Yunxia Li[1], Wenchao Li[1], Xiaolu Chen[1], Tonghui Yuan[1], Congcong Liu[1], Hongkai Li[1,2,3], Lei Hou[1], Bojie Wang[1], Fuzhong Xue[1,2,3,*](0000-0003-0378-7956)

1. Department of Biostatistics, School of Public Health, Cheeloo College of Medicine, Shandong University, Jinan, 250012, P.R. China.

2. Institute for Medical Dataology, Shandong University, Jinan, 250002, P.R. China.

3. National Institute of Health Data Science of China, Jinan, China

* Correspondence to: Fuzhong Xue, PhD, Department of Biostatistics, School of Public Health, Cheeloo College of Medicine, Shandong University, No.44 Wenhuaxi Road, Jinan, 250012, China. (Email: xuefzh@sdu.edu.cn).

Content

Table S1. Definition of cardio-cerebrovascular metabolic outcomes in the UK Biobank..4
Table S2. Information of internal instrumental SNPs in UK Biobank cohort. 5
Table S3. Information of external instrumental SNPs reported by other studies.............16
Table S4. Baseline characteristics of participants by 2×2 factorial groups in dataset A. 20
Table S5. Baseline characteristics of participants by 2×2 factorial groups in dataset B..21
Table S6. MR-egger test for potential horizontal pleiotropic...22
Table S7. Subgroup analysis for combined exposure to GLU and TG. 23
Table S8. Subgroup analysis for TyG index. 30
Figure S1. ROC curve for internal/external and weighted/unweighted GRS. 37
Figure S2. Assessment of independent higher GLU in scaled difference with the risk of minor CVD by weighted GRS in dataset A. 38
Figure S3. Assessment of independent higher TG in scaled difference with the risk of minor CVD by weighted GRS in dataset A. 39
Figure S4. Assessment of independent higher GLU and TG in crude difference with the risk of major CVD by weighted GRS in dataset A. 40
Figure S5. Assessment of independent higher GLU in crude difference with the risk of minor CVD by weighted GRS in dataset A. 41
Figure S6. Assessment of independent higher TG in crude difference with the risk of minor CVD by weighted GRS in dataset A. 42
Figure S7. Assessment of independent higher GLU and TG in scaled difference with the risk of major CVD by unweighted GRS in dataset A. 43
Figure S8. Assessment of independent higher GLU in scaled difference with the risk of minor CVD by unweighted GRS in dataset A. 44
Figure S9. Assessment of independent higher TG in scaled difference with the risk of minor CVD by unweighted GRS in dataset A. 45
Figure S10. Assessment of independent higher GLU and TG in scaled difference with the risk of major CVD by unweighted GRS in dataset B...46
Figure S11. Assessment of independent higher GLU in scaled difference with the risk of minor CVD by unweighted GRS in dataset B..47
Figure S12. Assessment of independent higher TG in scaled difference with the risk of minor CVD by unweighted GRS in dataset B..48
Figure S13. Assessment of independent higher GLU and TG in scaled difference with the risk of major CVD by weighted external reported SNPs in dataset A. 49
Figure S14. Assessment of independent higher GLU in scaled difference with the risk of minor CVD by weighted external reported SNPs in dataset A. 50
Figure S15. Assessment of independent higher TG in scaled difference with the risk of minor CVD by weighted external reported SNPs in dataset A. 51
Figure S16. Assessment of independent higher GLU and TG in scaled difference with the risk of major CVD by observational phenotypes in dataset A. 52
Figure S17. Assessment of independent higher GLU in scaled difference with the risk of minor CVD by observational phenotypes in dataset A. 53
Figure S18. Assessment of independent higher TG in scaled difference with the risk of

minor CVD by observational phenotypes in dataset A. 54
Figure S19. Association of combined exposure to GLU and TG in scaled difference with the risk of CVD by weighted GRS in dataset A........ 55
Figure S20. Association of combined exposure to GLU and TG in crude difference with the risk of CVD by unweighted GRS in dataset A. 56
Figure S21. Association of combined exposure to GLU and TG in crude difference with the risk of CVD by weighted GRS in dataset B. 57
Figure S22. Association of combined exposure to GLU and TG in crude difference with the risk of CVD by weighted external reported SNPs in dataset A. 58
Figure S23. Association of combined exposure to GLU and TG in crude difference with the risk of CVD by observational phenotypes in dataset A. 59
Figure S24. Association of TyG index with the risk of CVD by unweighted GRS in dataset A. 60
Figure S25. Association of TyG index with the risk of CVD by weighted GRS in dataset B. 61
Figure S26. Association of TyG index with the risk of CVD by weighted external reported SNPs in dataset A. 62
Figure S27. Association of observational TyG index with the risk of CVD in dataset A. . 63
Figure S28. Dose-response associations of meta-regression for combined glucose and triglyceride on the risk of CVD and IHD by unweighted GRS in dataset A. 64
Figure S29. Dose-response associations of meta-regression for combined glucose and triglyceride on the risk of CVD and IHD by weighted GRS in dataset B. 65
Figure S30. Dose-response associations of meta-regression for combined glucose and triglyceride on the risk of CVD and IHD by weighted external reported SNPs in dataset A. 66
Figure S31. Dose-response associations of meta-regression for combined glucose and triglyceride on the risk of CVD and IHD by observational phenotypes in dataset A. 67
Figure S32. Association of combined GLU and TG with the risk of CVD in the subgroup in dataset A. 68
Figure S33. The effect of continuous GLU, TG, and TyG index on CVD and subtypes in dataset A. 69
Figure S34. The association of GLU, TG, and TyG index with major CVD in external verification. 70
Figure S35. The association of GLU, TG, and TyG index with major CVD in external verification adjusted for BMI. 71

Table S1. Definition of cardio-cerebrovascular metabolic outcomes in the UK Biobank.

Disease	Code in UK Biobank
Overall cardiovascular and cerebrovascular metabolic disease	ICD10: I20-I25, I50, I60-I69<br>ICD9: 410-414, 428, 430-438<br>Self-report code: 1074, 1075, 1076, 1086, 1491, 1083, 1583, 1081
Ischaemic heart diseases	ICD10: I20-I25<br>ICD9: 410-414<br>Self-report code: 1074, 1075
Cerebrovascular diseases	ICD10: I60-I69<br>ICD9: 430-438<br>Self-report code: 1086, 1491, 1083, 1583, 1081
Angina pectoris	ICD10: I20<br>ICD9: 413<br>Self-report code: 1074
Acute myocardial infarction	ICD10: I21-I24<br>ICD9: 410-413<br>Self-report code: 1075
Chronic ischaemic heart disease	ICD10: I25<br>ICD9: 414<br>Self-report code: NA
Heart failure	ICD10: I50<br>ICD9: 428<br>Self-report code: 1076
Hemorrhagic stroke	ICD10: I60-I62<br>ICD9: 430-432<br>Self-report code: 1083, 1086, 1491
Ischaemic stroke	ICD10: I63, I65, I66<br>ICD9: 433-434<br>Self-report code: 1583
Diabetes mellitus	ICD10: E10-E14<br>ICD9: 250<br>Self-report code: 1220, 1222, 1223, 1468, 1607
Disorders of lipoprotein metabolism	ICD10: E78<br>ICD9: 272<br>Self-report code: 1473

Table S2. Information of internal instrumental SNPs in UK Biobank cohort.

No.	SNP	REF	ALT	BETA	SE	P	Phenotype
1	rs114165349	G	C	0.0474481	0.00444682	1.42182E-26	TG
2	rs213498	T	A	-0.00762761	0.00139905	4.98514E-08	TG
3	rs10889332	C	T	-0.0389253	0.00136702	4.4233E-178	TG
4	rs72669514	C	T	0.0195218	0.00306502	1.9029E-10	TG
5	rs1782815	A	C	0.0105244	0.00176558	2.51195E-09	TG
6	rs16836630	G	C	-0.0151299	0.00240964	3.4144E-10	TG
7	rs1760801	G	A	-0.0089832	0.00144549	5.15146E-10	TG
8	rs115276619	T	A	-0.0329127	0.00549343	2.08455E-09	TG
9	rs3897379	G	A	0.0114914	0.00166781	5.58649E-12	TG
10	rs61830291	A	C	0.0150972	0.00222378	1.13134E-11	TG
11	rs4846922	C	T	0.0224411	0.00139817	6.05241E-58	TG
12	rs907866	G	A	-0.00924841	0.00132625	3.10216E-12	TG
13	rs111585158	C	T	0.0124922	0.00202046	6.30536E-10	TG
14	rs76384951	A	C	-0.0305494	0.00241217	9.49983E-37	TG
15	rs533617	T	C	-0.0489754	0.00331386	2.09243E-49	TG
16	rs35750610	T	C	0.0170528	0.00224311	2.91863E-14	TG
17	rs34921778	A	G	0.00778415	0.0013859	1.94849E-08	TG
18	rs12617848	C	T	0.0142877	0.00191724	9.20438E-14	TG
19	rs80216311	C	T	-0.0152351	0.00228395	2.55412E-11	TG
20	rs61737373	G	A	-0.0309149	0.00279914	2.36481E-28	TG
21	rs56242350	A	G	-0.0280678	0.00470612	2.46257E-09	TG
22	rs10206462	T	C	-0.00813719	0.00136453	2.47385E-09	TG
23	rs6760053	C	G	-0.00727147	0.00131624	3.30905E-08	TG
24	rs79953491	A	G	-0.0226369	0.00201482	2.78084E-29	TG
25	rs6433249	A	T	0.00847441	0.0013682	5.88048E-10	TG
26	rs2278530	A	G	-0.0078909	0.00132677	2.72648E-09	TG
27	rs2943645	T	C	-0.0208738	0.00136824	1.58049E-52	TG
28	rs7596814	G	T	-0.00833088	0.00144535	8.2276E-09	TG
29	rs11715261	G	C	-0.013726	0.00250917	4.49579E-08	TG
30	rs73136795	G	A	0.0132215	0.00210008	3.06507E-10	TG
31	rs390802	G	A	-0.0158788	0.0016866	4.78004E-21	TG
32	rs62271373	T	A	0.0243757	0.00290459	4.7963E-17	TG
33	rs13074711	T	C	0.0114012	0.00208837	4.78266E-08	TG
34	rs13108218	G	A	0.0152879	0.00137454	9.92862E-29	TG
35	rs71603401	A	G	0.011269	0.0019637	9.55446E-09	TG
36	rs6448429	C	T	0.0125008	0.00179502	3.31153E-12	TG
37	rs9998485	A	G	-0.00770907	0.00134229	9.30016E-09	TG
38	rs1471251	A	T	0.0163899	0.00134529	3.90079E-34	TG
39	rs4134363	G	A	-0.00916993	0.00162651	1.72419E-08	TG
40	rs9991328	C	T	0.00870671	0.0013195	4.16131E-11	TG
41	rs2035816	A	G	-0.0149703	0.00238055	3.20885E-10	TG
42	rs78025076	C	T	0.0255399	0.00461429	3.11584E-08	TG

No.	SNP	REF	ALT	BETA	SE	P	Phenotype
43	rs1694068	A	T	-0.00769126	0.00135381	1.33892E-08	TG
44	rs40567	T	G	-0.0122152	0.00175386	3.29739E-12	TG
45	rs3936511	A	G	0.0210181	0.00166931	2.42503E-36	TG
46	rs151913	G	A	0.00793661	0.00135121	4.26699E-09	TG
47	rs7703744	C	G	-0.0100225	0.00148307	1.40252E-11	TG
48	rs11134475	G	A	-0.0168149	0.00136024	4.29914E-35	TG
49	rs2963476	A	G	0.01224	0.0016237	4.77498E-14	TG
50	rs1117488	G	T	-0.00957407	0.00154905	6.39416E-10	TG
51	rs2894211	C	A	0.0178255	0.0020917	1.57541E-17	TG
52	rs7758790	T	C	0.014167	0.00157506	2.38577E-19	TG
53	rs55697600	A	G	0.0354985	0.00344485	6.7735E-25	TG
54	rs149268820	G	A	0.0202365	0.00313461	1.07801E-10	TG
55	rs3025053	G	A	-0.0132403	0.00203085	7.06157E-11	TG
56	rs6458867	G	A	0.00991944	0.00137561	5.57192E-13	TG
57	rs2800713	G	A	0.0193064	0.00292568	4.14902E-11	TG
58	rs6916318	T	A	-0.0154098	0.00131662	1.23688E-31	TG
59	rs7451021	C	T	-0.00844877	0.00141525	2.37834E-09	TG
60	rs632057	G	T	0.0154436	0.00135792	5.78918E-30	TG
61	rs9403164	G	C	-0.00926081	0.00168056	3.58061E-08	TG
62	rs12208357	C	T	0.0226794	0.0026041	3.079E-18	TG
63	rs4709746	C	T	-0.0113782	0.00194206	4.66651E-09	TG
64	rs852392	G	A	0.00871299	0.00158842	4.13094E-08	TG
65	rs38205	C	A	0.00765656	0.00137587	2.62568E-08	TG
66	rs2106727	G	A	-0.00966587	0.00136507	1.43618E-12	TG
67	rs17138358	G	C	0.00771059	0.00134426	9.70832E-09	TG
68	rs4722551	T	C	-0.0190526	0.00179769	3.06865E-26	TG
69	rs1534696	A	C	0.00992776	0.00131553	4.48138E-14	TG
70	rs2971676	G	A	0.0127807	0.00229236	2.47267E-08	TG
71	rs183867209	T	C	-0.0221865	0.00260084	1.46444E-17	TG
72	rs799157	C	T	0.0420029	0.00325128	3.61376E-38	TG
73	rs13234131	A	G	-0.06337	0.00194942	2.3539E-231	TG
74	rs10260148	C	T	0.0147706	0.0014735	1.20468E-23	TG
75	rs13223343	A	C	0.00860614	0.00156283	3.65843E-08	TG
76	rs7821812	G	C	0.0169479	0.00162229	1.53123E-25	TG
77	rs904009	A	C	0.015918	0.00155418	1.298E-24	TG
78	rs7508	A	G	-0.00811404	0.00147772	4.00205E-08	TG
79	rs4921914	T	C	0.0186841	0.00158577	4.9015E-32	TG
80	rs7012105	G	C	0.013255	0.00161172	1.97421E-16	TG
81	rs77557266	A	G	-0.0183926	0.00184303	1.89053E-23	TG
82	rs268	A	G	0.11244	0.0049239	2.6138E-115	TG
83	rs287	A	G	-0.0593077	0.00152434	0	TG
84	rs142288236	C	T	0.0364855	0.00561022	7.86607E-11	TG
85	rs7000494	G	C	0.0688147	0.00395379	8.26319E-68	TG

No.	SNP	REF	ALT	BETA	SE	P	Phenotype
86	rs140064750	T	C	0.028162	0.00464296	1.31704E-09	TG
87	rs118045108	C	T	0.0297384	0.00435181	8.29969E-12	TG
88	rs28550053	A	G	-0.0178646	0.00174051	1.03464E-24	TG
89	rs28742908	C	A	-0.0131395	0.0013433	1.36323E-22	TG
90	rs73210884	G	A	-0.00865583	0.00150108	8.1081E-09	TG
91	rs73224085	C	T	0.00958057	0.0017014	1.79364E-08	TG
92	rs2081687	C	T	0.0125543	0.00138949	1.6472E-19	TG
93	rs71525127	C	G	0.0186655	0.00243479	1.77813E-14	TG
94	rs17321515	A	G	-0.0437663	0.00131055	4.9765E-244	TG
95	rs78309295	A	G	0.0256463	0.00443382	7.29143E-09	TG
96	rs62521590	T	G	0.0145444	0.00148865	1.52509E-22	TG
97	rs13289566	C	T	-0.0119283	0.00159506	7.55349E-14	TG
98	rs2244278	C	A	-0.0132459	0.00202501	6.11579E-11	TG
99	rs75113691	G	C	-0.0193972	0.00352584	3.77012E-08	TG
100	rs17134531	G	A	-0.0113886	0.00181489	3.49894E-10	TG
101	rs10826100	G	A	-0.00732206	0.0013298	3.67149E-08	TG
102	rs1171616	T	G	-0.00987224	0.00155685	2.28372E-10	TG
103	rs142164605	T	A	-0.0165316	0.00265831	5.01636E-10	TG
104	rs10786069	T	C	0.0124112	0.00131732	4.47823E-21	TG
105	rs113344423	G	A	0.02159	0.00288455	7.19221E-14	TG
106	rs2803609	G	A	-0.0102415	0.00147042	3.29113E-12	TG
107	rs55645497	T	A	0.0102586	0.00172172	2.55079E-09	TG
108	rs10832027	A	G	-0.0122768	0.00141683	4.5421E-18	TG
109	rs76613195	C	A	-0.0109206	0.00189742	8.64953E-09	TG
110	rs35169799	C	T	0.0247583	0.00270753	6.04499E-20	TG
111	rs678614	C	A	0.0101123	0.00146441	5.01783E-12	TG
112	rs2302883	T	C	0.00908916	0.00155113	4.64196E-09	TG
113	rs678904	T	A	0.00827292	0.00141013	4.44849E-09	TG
114	rs647071	G	C	0.00780391	0.00134659	6.82615E-09	TG
115	rs187217942	G	A	0.028266	0.00516384	4.40798E-08	TG
116	rs17119701	A	G	0.0367684	0.00358447	1.10394E-24	TG
117	rs61362984	A	G	-0.0138841	0.00135921	1.72016E-24	TG
118	rs61904855	C	A	0.023066	0.00391529	3.83687E-09	TG
119	rs11216117	C	A	0.101108	0.00255121	0	TG
120	rs11216122	G	T	-0.0202963	0.00296612	7.78668E-12	TG
121	rs7930786	G	C	0.125546	0.00266243	0	TG
122	rs2075294	G	T	0.0404176	0.00554258	3.05725E-13	TG
123	rs75919952	C	T	-0.0466964	0.00302955	1.39583E-53	TG
124	rs11600380	T	C	-0.0369004	0.00242821	3.91452E-52	TG
125	rs12721078	C	A	-0.032761	0.00376331	3.1825E-18	TG
126	rs71480323	G	A	-0.0198306	0.00202106	1.00845E-22	TG
127	rs11216236	C	T	0.024291	0.00327258	1.15185E-13	TG
128	rs61903415	A	G	-0.015936	0.00206506	1.19513E-14	TG

No.	SNP	REF	ALT	BETA	SE	P	Phenotype
129	rs187929675	C	T	-0.0776417	0.00579892	7.22396E-41	TG
130	rs11045171	A	G	-0.0119379	0.00166026	6.47847E-13	TG
131	rs67981690	A	G	0.0144658	0.00198305	3.00111E-13	TG
132	rs11183127	T	C	0.00866648	0.00155351	2.42629E-08	TG
133	rs10783828	G	A	0.0085335	0.00140975	1.42197E-09	TG
134	rs7296326	T	C	-0.01146	0.00207649	3.41374E-08	TG
135	rs4435046	C	A	-0.00727601	0.00131681	3.28877E-08	TG
136	rs10861679	T	C	0.0100321	0.00143999	3.25066E-12	TG
137	rs150362789	C	A	-0.0112743	0.00163807	5.88558E-12	TG
138	rs12301673	C	T	-0.0141473	0.0013901	2.53254E-24	TG
139	rs863750	T	C	-0.0145788	0.00134707	2.72571E-27	TG
140	rs7140110	T	C	0.015075	0.00144131	1.34403E-25	TG
141	rs112740904	T	G	-0.0142211	0.00187171	3.01916E-14	TG
142	rs12897637	T	C	0.0102005	0.00180382	1.56036E-08	TG
143	rs35477346	T	C	0.00801648	0.00143102	2.12192E-08	TG
144	rs139974673	T	C	0.0729373	0.00423408	1.83046E-66	TG
145	rs28607776	A	G	-0.0119681	0.00198016	1.50545E-09	TG
146	rs1532085	G	A	0.0182037	0.00134799	1.52034E-41	TG
147	rs261334	C	G	0.0251625	0.00160348	1.80023E-55	TG
148	rs11636087	T	C	0.0121246	0.00147671	2.21168E-16	TG
149	rs8028620	T	C	-0.00829158	0.0013139	2.7827E-10	TG
150	rs7175132	A	G	-0.00771376	0.00135116	1.13762E-08	TG
151	rs8025505	C	T	0.0104853	0.00151413	4.37039E-12	TG
152	rs1684608	C	A	0.00949503	0.00166887	1.27552E-08	TG
153	rs9935836	A	C	0.00944214	0.00169461	2.52286E-08	TG
154	rs11075253	C	A	-0.0145289	0.0014355	4.49736E-24	TG
155	rs5880	G	C	0.0212821	0.00284893	8.02987E-14	TG
156	rs12934528	T	C	0.0140557	0.00187146	5.90551E-14	TG
157	rs2925979	C	T	0.0152245	0.00143185	2.12388E-26	TG
158	rs9905191	C	T	0.0191155	0.00277441	5.59458E-12	TG
159	rs4793090	A	G	0.00896774	0.00138498	9.49864E-11	TG
160	rs72836561	C	T	0.0690761	0.00372651	1.17075E-76	TG
161	rs1801689	A	C	-0.0288134	0.00390049	1.50465E-13	TG
162	rs114248359	C	A	-0.0151483	0.00266665	1.34326E-08	TG
163	rs77244849	T	C	-0.008385	0.00142239	3.75143E-09	TG
164	rs12451715	C	A	0.0088804	0.00134203	3.66863E-11	TG
165	rs12955996	C	T	-0.0163597	0.00261087	3.71015E-10	TG
166	rs8092347	A	G	0.00817058	0.0013488	1.38309E-09	TG
167	rs263067	C	A	-0.00893997	0.00140012	1.71489E-10	TG
168	rs1035941	G	A	0.0109507	0.00146526	7.82935E-14	TG
169	rs4804413	C	T	0.00919008	0.00132396	3.89244E-12	TG
170	rs116843064	G	A	-0.109632	0.00469392	1.5677E-120	TG
171	rs57192995	G	C	-0.0202215	0.00289005	2.62229E-12	TG

No.	SNP	REF	ALT	BETA	SE	P	Phenotype
172	rs58542926	C	T	-0.0535288	0.00246291	1.2015E-104	TG
173	rs188247550	C	T	-0.064097	0.00613707	1.57463E-25	TG
174	rs11671010	T	C	0.0126972	0.00170008	8.13065E-14	TG
175	rs541012177	G	T	0.0253803	0.00337964	5.94136E-14	TG
176	rs41290100	C	T	-0.0248275	0.00424748	5.06485E-09	TG
177	rs41290108	G	C	-0.0217497	0.00341152	1.82817E-10	TG
178	rs11667640	C	T	-0.0254875	0.00269727	3.43408E-21	TG
179	rs72654472	G	T	-0.0329043	0.004001	1.97623E-16	TG
180	rs483082	G	T	0.0453986	0.00154495	1.6907E-189	TG
181	rs79429216	G	A	0.0386075	0.00601992	1.42647E-10	TG
182	rs146390218	A	G	0.035511	0.00415825	1.3501E-17	TG
183	rs62132802	C	T	-0.00893069	0.00143608	5.01659E-10	TG
184	rs142385484	C	T	-0.0117967	0.00190342	5.74001E-10	TG
185	rs2902745	G	T	0.0164709	0.00204845	8.97141E-16	TG
186	rs2207132	G	A	0.0291442	0.00370676	3.78068E-15	TG
187	rs6073958	T	C	0.0286387	0.00164789	1.29426E-67	TG
188	rs4812995	T	C	0.0092951	0.00154578	1.82112E-09	TG
189	rs6066138	G	A	-0.00818147	0.00145609	1.92504E-08	TG
190	rs6090040	C	A	0.00807134	0.00132682	1.1796E-09	TG
191	rs2277844	A	G	-0.00904792	0.00132383	8.23652E-12	TG
192	rs4253749	G	A	0.00888358	0.00160714	3.25006E-08	TG
193	rs9787076	A	C	0.00993509	0.00176983	1.98383E-08	GLU
194	rs79435107	G	T	0.0148107	0.00223161	3.21139E-11	GLU
195	rs1966228	A	G	-0.01308	0.00192633	1.12271E-11	GLU
196	rs2075423	G	T	-0.0174423	0.00177337	7.9751E-23	GLU
197	rs12724128	C	T	0.011492	0.00207043	2.85044E-08	GLU
198	rs13391244	T	C	0.0101232	0.00183272	3.32446E-08	GLU
199	rs12712928	G	C	0.0177943	0.00223902	1.91299E-15	GLU
200	rs1220129	C	T	0.0173309	0.00245075	1.53422E-12	GLU
201	rs12692596	C	T	0.0108159	0.00171944	3.17202E-10	GLU
202	rs6752810	G	C	-0.0190316	0.00290493	5.7075E-11	GLU
203	rs114072917	C	T	-0.0265467	0.00469056	1.51875E-08	GLU
204	rs4668101	G	A	-0.0408559	0.00320038	2.60362E-37	GLU
205	rs138293199	A	C	0.0464344	0.00711402	6.71385E-11	GLU
206	rs560494	G	T	0.0293571	0.00216936	1.03598E-41	GLU
207	rs116710147	C	T	0.0431224	0.00749295	8.66992E-09	GLU
208	rs71430651	G	A	0.0562608	0.00653608	7.49054E-18	GLU
209	rs78200061	A	G	0.040945	0.00657806	4.8384E-10	GLU
210	rs115128825	C	A	0.0718658	0.00589647	3.67869E-34	GLU
211	rs2232315	G	A	0.0393127	0.00605869	8.67508E-11	GLU
212	rs484066	T	A	-0.07413	0.00171058	0	GLU
213	rs372482560	C	T	0.0417674	0.00760142	3.91778E-08	GLU
214	rs11708067	A	G	-0.0297001	0.00192354	9.23912E-54	GLU

No.	SNP	REF	ALT	BETA	SE	P	Phenotype
215	rs113861493	A	G	-0.0320571	0.00401229	1.35758E-15	GLU
216	rs73174306	A	T	0.0252405	0.0041131	8.44217E-10	GLU
217	rs74598960	T	C	-0.0214623	0.00367621	5.28433E-09	GLU
218	rs6840504	C	T	0.0104611	0.00168617	5.50936E-10	GLU
219	rs58515625	T	C	-0.017132	0.00239344	8.21109E-13	GLU
220	rs7708285	A	G	0.0130581	0.00179616	3.60384E-13	GLU
221	rs9285019	C	T	-0.0185972	0.00184236	5.91752E-24	GLU
222	rs59598688	T	C	0.0100142	0.0016679	1.9266E-09	GLU
223	rs3756602	A	G	0.0143449	0.00238874	1.91333E-09	GLU
224	rs35742417	C	A	-0.019572	0.00212803	3.69872E-20	GLU
225	rs7766070	C	A	0.0132743	0.00189011	2.17663E-12	GLU
226	rs13198907	T	A	-0.00959814	0.00165928	7.28045E-09	GLU
227	rs2750417	T	C	-0.0117465	0.00177949	4.09009E-11	GLU
228	rs17168486	C	T	0.0165994	0.00220983	5.85845E-14	GLU
229	rs62448618	A	T	-0.021227	0.00167033	5.45305E-37	GLU
230	rs76323047	A	G	0.0344288	0.00259581	3.89538E-40	GLU
231	rs112484505	G	A	-0.0457814	0.00620445	1.60188E-13	GLU
232	rs741037	G	A	0.0699376	0.00216721	4.8396E-228	GLU
233	rs138917529	A	T	-0.0683718	0.00706867	3.97834E-22	GLU
234	rs2715131	T	C	0.0124704	0.0019822	3.15533E-10	GLU
235	rs11773850	G	A	0.031007	0.0056243	3.52993E-08	GLU
236	rs9987289	G	A	0.0230461	0.00285772	7.38327E-16	GLU
237	rs62523081	G	A	0.00946451	0.00173168	4.61994E-08	GLU
238	rs3802177	G	A	-0.0263302	0.00179116	6.7205E-49	GLU
239	rs4237150	G	C	0.0143283	0.00169567	2.92851E-17	GLU
240	rs4258054	T	C	-0.0110812	0.0018921	4.73149E-09	GLU
241	rs10811662	G	A	-0.0169494	0.00218306	8.25229E-15	GLU
242	rs706115	C	G	-0.0150415	0.00227794	4.03408E-11	GLU
243	rs143858621	C	T	-0.0323913	0.005277	8.35715E-10	GLU
244	rs28533815	C	T	-0.0138657	0.00193164	7.08319E-13	GLU
245	rs11257655	C	T	0.013043	0.00204544	1.81329E-10	GLU
246	rs8190647	C	T	-0.0121655	0.00217547	2.24523E-08	GLU
247	rs7075575	G	A	-0.01163	0.00181659	1.53511E-10	GLU
248	rs7895494	A	C	-0.010143	0.0017274	4.31483E-09	GLU
249	rs76385632	C	G	-0.0309417	0.00292346	3.57952E-26	GLU
250	rs3842752	G	A	0.0160665	0.00197954	4.82583E-16	GLU
251	rs141521721	C	A	0.0298797	0.00543118	3.76876E-08	GLU
252	rs2856653	C	T	-0.0159173	0.00174878	8.93092E-20	GLU
253	rs11172211	C	G	-0.00919942	0.00166459	3.26893E-08	GLU
254	rs6538804	C	G	-0.0105164	0.00173502	1.35251E-09	GLU
255	rs34701644	C	T	0.0148003	0.00200882	1.7413E-13	GLU
256	rs11619319	A	G	0.0188694	0.00200373	4.67066E-21	GLU
257	rs7997912	T	C	0.0182092	0.00224294	4.74083E-16	GLU

No.	SNP	REF	ALT	BETA	SE	P	Phenotype
258	rs243169	G	A	0.0161127	0.00190642	2.88203E-17	GLU
259	rs1836996	T	C	-0.0139553	0.00166351	4.92324E-17	GLU
260	rs67507374	T	A	-0.010961	0.00181194	1.45628E-09	GLU
261	rs57943755	A	G	-0.0166472	0.00230688	5.35488E-13	GLU
262	rs12449517	T	G	-0.00988257	0.0017163	8.51834E-09	GLU
263	rs4264433	T	A	0.0095181	0.00166839	1.16487E-08	GLU
264	rs76388330	A	C	0.0156499	0.00278209	1.85429E-08	GLU
265	rs11077553	G	A	0.0109796	0.00199506	3.72871E-08	GLU
266	rs1964272	G	A	-0.0105085	0.00166123	2.52464E-10	GLU
267	rs6048183	A	G	-0.0299965	0.00432111	3.87862E-12	GLU
268	rs8356	C	T	-0.0121857	0.00187363	7.84504E-11	GLU
269	rs6026578	G	C	0.0099065	0.00172647	9.589E-09	GLU
270	rs114165349	G	C	0.0445298	0.00465259	1.06735E-21	TYG
271	rs72904790	T	C	-0.0137147	0.00240632	1.20326E-08	TYG
272	rs213498	T	A	-0.00802754	0.00146386	4.16621E-08	TYG
273	rs10889332	C	T	-0.0388967	0.00143046	1.3382E-162	TYG
274	rs72669514	C	T	0.018609	0.00320686	6.52566E-09	TYG
275	rs17656269	C	T	0.00874937	0.00147002	2.65405E-09	TYG
276	rs16836630	G	C	-0.0173377	0.00252122	6.13938E-12	TYG
277	rs1760801	G	A	-0.00895698	0.0015125	3.18509E-09	TYG
278	rs340836	T	C	-0.00869811	0.00139646	4.71102E-10	TYG
279	rs76172548	A	C	0.0228139	0.00383242	2.63796E-09	TYG
280	rs3120619	G	A	0.0118809	0.0018033	4.45332E-11	TYG
281	rs11118610	A	C	-0.00902819	0.00138913	8.08985E-11	TYG
282	rs4846922	C	T	0.0221545	0.00146286	8.63176E-52	TYG
283	rs907866	G	A	-0.00927654	0.00138756	2.30552E-11	TYG
284	rs111585158	C	T	0.0121133	0.00211397	1.00464E-08	TYG
285	rs144470864	A	C	0.0172612	0.00307793	2.04824E-08	TYG
286	rs76384951	A	C	-0.0295509	0.0025237	1.16168E-31	TYG
287	rs533617	T	C	-0.0464535	0.00346743	6.47625E-41	TYG
288	rs35750610	T	C	0.0185128	0.00234709	3.0936E-15	TYG
289	rs34921778	A	G	0.00842606	0.00144997	6.20955E-09	TYG
290	rs12617848	C	T	0.0142794	0.00200585	1.09099E-12	TYG
291	rs80216311	C	T	-0.0141686	0.00238957	3.04527E-09	TYG
292	rs61737373	G	A	-0.028607	0.00292878	1.5652E-22	TYG
293	rs6547692	A	G	0.0375073	0.00138448	2.0518E-161	TYG
294	rs10206462	T	C	-0.00893744	0.00142769	3.85345E-10	TYG
295	rs6760053	C	G	-0.00778921	0.00137713	1.54972E-08	TYG
296	rs6710938	A	C	-0.00898973	0.00161797	2.75984E-08	TYG
297	rs79953491	A	G	-0.0237048	0.00210804	2.48953E-29	TYG
298	rs115128825	C	A	0.026901	0.00487631	3.45763E-08	TYG
299	rs484066	T	A	-0.0159198	0.00141892	3.31444E-29	TYG
300	rs17694506	T	C	0.00900471	0.00141859	2.18964E-10	TYG

No.	SNP	REF	ALT	BETA	SE	P	Phenotype
301	rs2943645	T	C	-0.0209209	0.0014316	2.39752E-48	TYG
302	rs6437249	C	T	0.00838469	0.00149284	1.94953E-08	TYG
303	rs147764624	G	C	-0.03019	0.00550582	4.17837E-08	TYG
304	rs390802	G	A	-0.0153354	0.00176462	3.62724E-18	TYG
305	rs62271373	T	A	0.0253586	0.00303878	7.15497E-17	TYG
306	rs13074711	T	C	0.0120107	0.00218488	3.86212E-08	TYG
307	rs13108218	G	A	0.0156395	0.00143815	1.54185E-27	TYG
308	rs71603401	A	G	0.0125089	0.00205483	1.14786E-09	TYG
309	rs6448429	C	T	0.0126753	0.00187808	1.49083E-11	TYG
310	rs1471251	A	T	0.0164471	0.00140761	1.55816E-31	TYG
311	rs4134363	G	A	-0.00950005	0.00170166	2.36868E-08	TYG
312	rs3822076	T	A	0.00867089	0.0013818	3.50043E-10	TYG
313	rs2035816	A	G	-0.0159447	0.00249077	1.54069E-10	TYG
314	rs78025076	C	T	0.0271124	0.00482777	1.95708E-08	TYG
315	rs390556	T	C	-0.0132704	0.00220378	1.72864E-09	TYG
316	rs72754154	G	A	-0.0213999	0.00314107	9.58248E-12	TYG
317	rs3936511	A	G	0.0216126	0.00174659	3.68546E-35	TYG
318	rs151913	G	A	0.00786984	0.00141384	2.6049E-08	TYG
319	rs7703744	C	G	-0.0109345	0.00155172	1.83654E-12	TYG
320	rs72801474	G	A	-0.0146422	0.00235286	4.8802E-10	TYG
321	rs12173130	T	C	0.00971075	0.00176945	4.06883E-08	TYG
322	rs11134475	G	A	-0.0169417	0.00142319	1.14894E-32	TYG
323	rs2963476	A	G	0.0133691	0.00169886	3.57579E-15	TYG
324	rs6923241	C	T	-0.0109234	0.00154721	1.66805E-12	TYG
325	rs2745400	G	A	0.00826032	0.00137239	1.75789E-09	TYG
326	rs2894211	C	A	0.0174652	0.00218851	1.46384E-15	TYG
327	rs7758790	T	C	0.0142803	0.00164795	4.51962E-18	TYG
328	rs55697600	A	G	0.0351072	0.00360432	2.04622E-22	TYG
329	rs185139895	G	A	0.0207564	0.00338431	8.62902E-10	TYG
330	rs3025053	G	A	-0.0134581	0.00212481	2.39594E-10	TYG
331	rs4715317	G	T	0.00974329	0.00143947	1.30219E-11	TYG
332	rs1967685	G	C	-0.0142769	0.0013714	2.24508E-25	TYG
333	rs632057	G	T	0.0153665	0.00142077	2.9415E-27	TYG
334	rs12208357	C	T	0.0219558	0.00272455	7.7538E-16	TYG
335	rs77009508	A	G	0.0224178	0.00259593	5.86411E-18	TYG
336	rs55730499	C	T	-0.0182481	0.0025524	8.73842E-13	TYG
337	rs186696265	C	T	-0.0472632	0.00592501	1.50634E-15	TYG
338	rs4709746	C	T	-0.0112466	0.00203182	3.1114E-08	TYG
339	rs852424	C	T	0.00852818	0.0014628	5.54712E-09	TYG
340	rs38205	C	A	0.00791466	0.00143983	3.86804E-08	TYG
341	rs2106727	G	A	-0.0108194	0.00142823	3.59218E-14	TYG
342	rs4722551	T	C	-0.0185803	0.00188091	5.21341E-23	TYG
343	rs1534696	A	C	0.0107368	0.00137638	6.17667E-15	TYG

No.	SNP	REF	ALT	BETA	SE	P	Phenotype
344	rs2971676	G	A	0.0133494	0.00239848	2.61247E-08	TYG
345	rs878521	G	A	0.0217619	0.00158662	8.43491E-43	TYG
346	rs62459110	G	C	-0.0213924	0.00364332	4.31891E-09	TYG
347	rs799157	C	T	0.0407906	0.00340184	4.05026E-33	TYG
348	rs17145750	C	T	-0.0560614	0.00185642	5.2775E-200	TYG
349	rs10260148	C	T	0.0149589	0.00154174	2.96412E-22	TYG
350	rs73198299	T	C	0.0122709	0.00222876	3.68068E-08	TYG
351	rs7821812	G	C	0.0163357	0.00169741	6.38886E-22	TYG
352	rs904009	A	C	0.0159306	0.00162585	1.15506E-22	TYG
353	rs4921914	T	C	0.0194386	0.00165914	1.07361E-31	TYG
354	rs2975424	T	C	0.0106046	0.0017554	1.53218E-09	TYG
355	rs1388941	G	A	0.0143536	0.00145941	8.01352E-23	TYG
356	rs268	A	G	0.10865	0.00515243	1.25248E-98	TYG
357	rs117026536	G	T	-0.0951759	0.00226332	0	TYG
358	rs57295072	G	C	-0.0304863	0.00469748	8.60288E-11	TYG
359	rs17091881	T	C	0.0785949	0.00427426	1.82213E-75	TYG
360	rs74444445	T	C	0.034928	0.00488632	8.8198E-13	TYG
361	rs117805502	C	T	-0.0321654	0.00439412	2.48432E-13	TYG
362	rs28550053	A	G	-0.0177064	0.00182104	2.42155E-22	TYG
363	rs75662196	G	C	-0.0279294	0.00434519	1.29803E-10	TYG
364	rs17092008	C	T	0.0208253	0.00285324	2.91106E-13	TYG
365	rs11781356	T	A	0.00993171	0.00176553	1.85318E-08	TYG
366	rs2081687	C	T	0.011677	0.00145382	9.63108E-16	TYG
367	rs71525127	C	G	0.019603	0.00254745	1.4181E-14	TYG
368	rs11558471	A	G	-0.011475	0.00147145	6.29222E-15	TYG
369	rs17321515	A	G	-0.0439	0.00137141	2.0055E-224	TYG
370	rs62521590	T	G	0.0146537	0.00155763	5.10758E-21	TYG
371	rs10811661	T	C	-0.00987338	0.0018054	4.53471E-08	TYG
372	rs13289566	C	T	-0.0118284	0.00166888	1.36744E-12	TYG
373	rs2244278	C	A	-0.0133902	0.00211876	2.62264E-10	TYG
374	rs3750571	C	A	-0.0124087	0.00189812	6.27151E-11	TYG
375	rs11006681	G	A	-0.0110061	0.00184368	2.38065E-09	TYG
376	rs142164605	T	A	-0.0177614	0.00278129	1.70522E-10	TYG
377	rs10786069	T	C	0.0130977	0.00137832	2.06173E-21	TYG
378	rs113344423	G	A	0.0212993	0.00301795	1.69875E-12	TYG
379	rs2792736	A	T	-0.0100505	0.00153811	6.40182E-11	TYG
380	rs10832027	A	G	-0.0122569	0.00148245	1.36822E-16	TYG
381	rs3808976	A	G	0.00981886	0.00170108	7.8353E-09	TYG
382	rs99780	C	T	0.020203	0.00143553	5.72743E-45	TYG
383	rs35169799	C	T	0.0247241	0.00283283	2.61306E-18	TYG
384	rs678614	C	A	0.00935072	0.00153217	1.04275E-09	TYG
385	rs2302883	T	C	0.00886478	0.00162296	4.70928E-08	TYG
386	rs187217942	G	A	0.031159	0.00540272	8.06492E-09	TYG

No.	SNP	REF	ALT	BETA	SE	P	Phenotype
387	rs17119701	A	G	0.0370675	0.00375036	4.9416E-23	TYG
388	rs61362984	A	G	-0.0139461	0.00142207	1.06033E-22	TYG
389	rs61904855	C	A	0.0233784	0.00409645	1.15104E-08	TYG
390	rs11216122	G	T	-0.0181908	0.00310342	4.59108E-09	TYG
391	rs7930786	G	C	0.124688	0.00278666	0	TYG
392	rs56225305	G	A	0.108415	0.00278601	0	TYG
393	rs2075294	G	T	0.0388118	0.00579905	2.19382E-11	TYG
394	rs75919952	C	T	-0.046805	0.0031699	2.55685E-49	TYG
395	rs11600380	T	C	-0.03673	0.00254055	2.3403E-47	TYG
396	rs5110	C	A	-0.0185398	0.00248476	8.58565E-14	TYG
397	rs12721078	C	A	-0.0322797	0.0039376	2.45839E-16	TYG
398	rs71480323	G	A	-0.0195603	0.00211444	2.24331E-20	TYG
399	rs11216236	C	T	0.0240392	0.00342403	2.21223E-12	TYG
400	rs187929675	C	T	-0.0767868	0.00606752	1.06977E-36	TYG
401	rs11045171	A	G	-0.0116623	0.00173721	1.90756E-11	TYG
402	rs67981690	A	G	0.0148562	0.00207488	8.08699E-13	TYG
403	rs10783828	G	A	0.00903509	0.00147493	9.03755E-10	TYG
404	rs7296326	T	C	-0.0118561	0.00217251	4.8374E-08	TYG
405	rs1585705	A	C	0.00876614	0.001496	4.64145E-09	TYG
406	rs10861679	T	C	0.00935439	0.00150663	5.34607E-10	TYG
407	rs1882491	T	C	-0.0134975	0.00148108	8.04481E-20	TYG
408	rs1716407	A	G	-0.0150613	0.00139861	4.89601E-27	TYG
409	rs7140110	T	C	0.0143695	0.00150803	1.60565E-21	TYG
410	rs112740904	T	G	-0.0149023	0.00195835	2.75811E-14	TYG
411	rs12885801	C	A	0.00908515	0.00162151	2.10994E-08	TYG
412	rs34820917	G	A	-0.0157999	0.00285011	2.96594E-08	TYG
413	rs35477346	T	C	0.00929684	0.00149721	5.32484E-10	TYG
414	rs139974673	T	C	0.0717689	0.00443008	5.3425E-59	TYG
415	rs72739147	A	T	-0.0121232	0.00206079	4.03892E-09	TYG
416	rs1532085	G	A	0.0180035	0.0014104	2.64155E-37	TYG
417	rs261334	C	G	0.0261448	0.00167767	9.88122E-55	TYG
418	rs11636087	T	C	0.0116533	0.00154515	4.64913E-14	TYG
419	rs8028620	T	C	-0.00897866	0.00137468	6.52538E-11	TYG
420	rs7175132	A	G	-0.00811532	0.00141367	9.44485E-09	TYG
421	rs8025505	C	T	0.00964703	0.00158419	1.13378E-09	TYG
422	rs9935836	A	C	0.00988172	0.00177279	2.49033E-08	TYG
423	rs11075253	C	A	-0.0141141	0.00150196	5.64657E-21	TYG
424	rs12446515	C	T	-0.0187602	0.00147498	4.75764E-37	TYG
425	rs5880	G	C	0.0221143	0.00298074	1.18303E-13	TYG
426	rs12934528	T	C	0.0135148	0.00195818	5.1503E-12	TYG
427	rs2925979	C	T	0.0154375	0.00149811	6.78754E-25	TYG
428	rs11651957	G	A	0.0186479	0.00293615	2.14066E-10	TYG
429	rs12937081	A	G	0.0108836	0.00189096	8.64186E-09	TYG

No.	SNP	REF	ALT	BETA	SE	P	Phenotype
430	rs72836561	C	T	0.0682178	0.00389919	1.69357E-68	TYG
431	rs231539	C	T	0.0130645	0.00188077	3.75707E-12	TYG
432	rs11657238	G	A	-0.00784868	0.00138378	1.41337E-08	TYG
433	rs1801689	A	C	-0.0293183	0.00408097	6.77966E-13	TYG
434	rs77244849	T	C	-0.00875261	0.00148835	4.08914E-09	TYG
435	rs9891030	G	A	0.00993826	0.00159406	4.53669E-10	TYG
436	rs71352934	A	C	-0.0163114	0.00273609	2.5014E-09	TYG
437	rs8092347	A	G	0.00812063	0.00141115	8.69431E-09	TYG
438	rs197156	A	G	-0.00925016	0.00145381	1.98528E-10	TYG
439	rs1035941	G	A	0.0110148	0.00153309	6.75221E-13	TYG
440	rs4804413	C	T	0.00948499	0.00138516	7.52701E-12	TYG
441	rs116843064	G	A	-0.108878	0.00491157	8.7939E-109	TYG
442	rs57192995	G	C	-0.019903	0.00302387	4.65156E-11	TYG
443	rs58542926	C	T	-0.0520456	0.00257716	1.26346E-90	TYG
444	rs188247550	C	T	-0.0644712	0.00642107	1.02053E-23	TYG
445	rs62102718	A	T	0.0115887	0.00152691	3.2178E-14	TYG
446	rs58895965	C	A	0.0127238	0.00180395	1.75142E-12	TYG
447	rs541012177	G	T	0.0242543	0.00353606	6.94208E-12	TYG
448	rs41290102	C	T	-0.0331177	0.00588779	1.85884E-08	TYG
449	rs419925	G	C	-0.0130424	0.00149941	3.38538E-18	TYG
450	rs483082	G	T	0.0446753	0.00161686	8.0918E-168	TYG
451	rs79429216	G	A	0.0378212	0.0062985	1.91817E-09	TYG
452	rs146390218	A	G	0.0355235	0.00435052	3.21892E-16	TYG
453	rs62132802	C	T	-0.00911832	0.00150245	1.28918E-09	TYG
454	rs12610709	G	A	0.013994	0.00183258	2.24391E-14	TYG
455	rs2207132	G	A	0.0283225	0.00387832	2.82644E-13	TYG
456	rs2250900	C	T	0.00899617	0.00163964	4.09926E-08	TYG
457	rs6073958	T	C	0.0274094	0.00172428	7.14303E-57	TYG
458	rs4812995	T	C	0.0091331	0.00161739	1.63621E-08	TYG
459	rs6066138	G	A	-0.00850564	0.00152344	2.36363E-08	TYG
460	rs6090040	C	A	0.0089174	0.00138822	1.33287E-10	TYG
461	rs2277844	A	G	-0.00908433	0.00138503	5.4289E-11	TYG

Table S3. Information of external instrumental SNPs reported by other studies.

No.	SNP	BETA	SE	A1	A2	P	Phenotype
1	rs10489615	-0.039	0.0027	G	A	4.2E-49	TG
2	rs1077514	0.019	0.0035	T	C	0.000000025	TG
3	rs10861661	0.019	0.0032	C	A	2.7E-09	TG
4	rs10889353	-0.077	0.0028	C	A	6.4E-170	TG
5	rs11057401	-0.028	0.0028	A	T	7.2E-23	TG
6	rs1126673	0.017	0.0029	T	C	9.9E-09	TG
7	rs116843064	-0.27	0.0097	A	G	4.2E-175	TG
8	rs12208357	0.032	0.0054	T	C	3.9E-09	TG
9	rs12453522	0.021	0.0034	G	A	1.2E-09	TG
10	rs12748152	0.037		T	C	0.000000001	TG
11	rs13326165	0.02	0.0033	G	A	9.7E-10	TG
12	rs1344642	-0.015	0.0026	A	G	0.000000014	TG
13	rs1495741	-0.035	0.003	A	G	8.1E-31	TG
14	rs1532085	-0.031	0.0026	G	A	4.6E-32	TG
15	rs1564348	0.02	0.0036	C	T	0.000000028	TG
16	rs1800588	0.047	0.003	T	C	8.6E-54	TG
17	rs1801177	0.17	0.01	A	G	1.1E-61	TG
18	rs1801689	-0.047	0.008	C	A	3.9E-09	TG
19	rs1832007	-0.033		G	A	2E-12	TG
20	rs1883025	-0.022	0.0029	T	C	1.2E-13	TG
21	rs2000999	0.021	0.0035	A	G	7.7E-10	TG
22	rs2068888	-0.032	0.0026	A	G	4.3E-34	TG
23	rs2081687	-0.019	0.0027	C	T	1.3E-11	TG
24	rs2287922	0.019	0.0028	A	G	4.8E-12	TG
25	rs2292642	-0.02	0.0026	T	C	3.4E-14	TG
26	rs247616	-0.036	0.0028	T	C	2.4E-38	TG
27	rs26008	-0.028	0.0048	C	T	5.3E-09	TG
28	rs268	0.24	0.01	G	A	1E-125	TG
29	rs2745353	0.02	0.0026	T	C	3.3E-15	TG
30	rs2785990	0.016	0.0028	T	C	0.000000012	TG
31	rs2792751	0.02	0.0029	C	T	1.4E-11	TG
32	rs28399654	0.073	0.0082	A	G	8.5E-19	TG
33	rs2844480	0.023	0.0033	T	C	5.4E-12	TG
34	rs2925979	-0.029	0.0028	C	T	1.2E-24	TG
35	rs2943641	0.033	0.0028	C	T	4.9E-33	TG
36	rs2954029	-0.08	0.0026	T	A	8.3E-205	TG
37	rs301	-0.12	0.0031	C	T	0	TG
38	rs3135506	0.24	0.0055	C	G	0	TG
39	rs35169799	0.038	0.0055	T	C	3.1E-12	TG
40	rs35332062	-0.12	0.0041	A	G	5.2E-205	TG
41	rs3748034	0.035	0.0042	T	G	4.7E-17	TG
42	rs3769823	0.017	0.0028	G	A	1.4E-09	TG

43	rs3803357	-0.017	0.0026	A	C	1.2E-10	TG
44	rs3873379	0.028	0.0029	C	T	7E-23	TG
45	rs38855	-0.019		G	A	0.00000002	TG
46	rs3927680	-0.018	0.0027	A	T	2E-11	TG
47	rs4149056	0.029	0.0038	C	T	3E-14	TG
48	rs4245791	-0.019	0.003	T	C	3.4E-10	TG
49	rs4311394	0.018	0.0029	G	A	4.7E-10	TG
50	rs439401	0.075	0.0027	C	T	2.7E-168	TG
51	rs4410790	0.015	0.0027	C	T	0.000000012	TG
52	rs442177	0.031	0.0026	T	G	4E-31	TG
53	rs4722551	-0.026	0.0037	C	T	4.4E-12	TG
54	rs4976033	0.018	0.0026	G	A	1.7E-11	TG
55	rs55707100	0.13	0.0085	T	C	8.6E-54	TG
56	rs58542926	-0.12	0.005	T	C	3.7E-125	TG
57	rs5880	0.039	0.0067	C	G	0.00000001	TG
58	rs6062343	-0.018	0.0027	A	G	4.1E-11	TG
59	rs61995676	0.093	0.0094	T	C	2.7E-23	TG
60	rs643381	-0.023	0.0026	A	C	2E-18	TG
61	rs6749689	-0.016	0.0026	C	T	1.1E-09	TG
62	rs676210	-0.071	0.0031	A	G	4.9E-118	TG
63	rs6882076	0.038	0.0027	C	T	1.2E-44	TG
64	rs7157785	0.023	0.0037	T	G	6.4E-10	TG
65	rs7200543	0.024	0.0029	G	A	5.6E-17	TG
66	rs7248104	-0.02	0.0026	A	G	1.8E-14	TG
67	rs72836561	0.13	0.0079	T	C	5.9E-64	TG
68	rs731839	0.022		G	A	0.000000003	TG
69	rs7350481	-0.23	0.0047	C	T	0	TG
70	rs738322	-0.02	0.0026	G	A	3.4E-14	TG
71	rs738409	-0.018	0.0031	G	C	4.3E-09	TG
72	rs7679	0.053	0.0035	C	T	2.4E-53	TG
73	rs7758229	0.018	0.0029	T	G	4.5E-10	TG
74	rs7901016	0.042	0.0056	C	T	5E-14	TG
75	rs7946	-0.016	0.0029	T	C	0.000000012	TG
76	rs9311651	-0.021	0.0036	G	A	2.6E-09	TG
77	rs9472138	-0.02	0.003	T	C	5E-11	TG
78	rs9686661	0.042	0.0033	T	C	2E-37	TG
79	rs998584	0.034	0.0027	A	C	1.2E-35	TG
80	rs1019503	0.0628	0.0109	A	G	8.87E-09	GLU
81	rs10747083	0.013344	0.002308	A	G	7.57E-09	GLU
82	rs10811661	0.0238	0.0028	T	C	5.6E-18	GLU
83	rs10814916	0.015819	0.002156	C	A	2.26E-13	GLU
84	rs10830963	0.0779	0.0025	G	C	0	GLU
85	rs11195502	0.032448	0.003702	C	T	1.97E-18	GLU
86	rs11558471	0.0289	0.0023	A	G	7.8E-37	GLU

87	rs11603334	0.0192	0.0028	G	A	1.1E-11	GLU
88	rs11605924	0.0202	0.0023	A	C	3.93E-19	GLU
89	rs11619319	0.0195	0.0024	G	A	1.3E-15	GLU
90	rs11708067	0.023	0.0026	A	G	1.3E-18	GLU
91	rs1280	0.026374	0.003067	T	C	8.56E-18	GLU
92	rs16913693	0.0434	0.0066	T	G	3.5E-11	GLU
93	rs2191349	0.0292	0.0021	T	G	0	GLU
94	rs2657879	0.0157	0.0029	G	A	0.000000039	GLU
95	rs340874	0.02		C	T	1.69E-13	GLU
96	rs3783347	0.0168	0.0026	G	T	1.3E-10	GLU
97	rs3829109	0.017249	0.002673	G	A	1.13E-10	GLU
98	rs4502156	0.022435	0.002144	T	C	1.38E-25	GLU
99	rs4869272	0.0177	0.0022	T	C	1E-15	GLU
100	rs560887	0.0711	0.0025	C	T	0	GLU
101	rs576674	0.016697	0.002984	G	A	2.26E-08	GLU
102	rs6072275	0.015924	0.002819	A	G	1.66E-08	GLU
103	rs6113722	0.035313	0.005287	G	A	2.49E-11	GLU
104	rs6943153	0.0154	0.0022	T	C	1.6E-12	GLU
105	rs730497	0.057464	0.002903	A	G	0	GLU
106	rs7651090	0.012838	0.002276	G	A	1.75E-08	GLU
107	rs7708285	0.015	0.0026	G	A	0.000000012	GLU
108	rs9368222	0.014251	0.002331	A	C	0.000000001	GLU
109	rs983309	0.025609	0.003282	T	G	6.29E-15	GLU
110	rs12748152	0.0184701	0.00253152	T	C	2.97071E-13	TYG
111	rs10889353	-0.0386213	0.00142965	C	A	1.6238E-160	TYG
112	rs10489615	0.0241455	0.00141046	A	G	1.1609E-65	TYG
113	rs676210	-0.0357831	0.00169405	A	G	5.8918E-99	TYG
114	rs1260326	0.0461227	0.00140559	T	C	1.0834E-235	TYG
115	rs560887	-0.0205745	0.00149507	T	C	4.48573E-43	TYG
116	rs2943641	-0.0206862	0.00143191	T	C	2.74161E-47	TYG
117	rs13326165	-0.0122007	0.00169483	A	G	6.09121E-13	TYG
118	rs3748034	0.0175337	0.00197191	T	G	6.05021E-19	TYG
119	rs442177	-0.0156929	0.00139532	G	T	2.43776E-29	TYG
120	rs9686661	0.0208186	0.00171603	T	C	7.30709E-34	TYG
121	rs6882076	-0.0169384	0.00142095	T	C	9.43231E-33	TYG
122	rs3873379	0.014718	0.00152609	C	T	5.24098E-22	TYG
123	rs2844480	0.0137414	0.00172605	T	C	1.71021E-15	TYG
124	rs61995676	0.0474907	0.00540829	T	C	1.62797E-18	TYG
125	rs998584	0.0192617	0.00138059	A	C	3.17906E-44	TYG
126	rs2745353	-0.0142542	0.00137129	C	T	2.64988E-25	TYG
127	rs643381	0.0140525	0.00137484	C	A	1.60937E-24	TYG
128	rs12208357	0.0219558	0.00272455	T	C	7.7538E-16	TYG
129	rs4410790	-0.0111451	0.00142489	T	C	5.22764E-15	TYG
130	rs4722551	-0.0185803	0.00188091	C	T	5.21341E-23	TYG

131	rs730497	0.0213329	0.00179597	A	G	1.56419E-32	TYG
132	rs35332062	-0.0609699	0.00204438	A	G	4.0354E-195	TYG
133	rs1495741	0.019957	0.00165657	G	A	2.04557E-33	TYG
134	rs1801177	0.0802336	0.00531625	A	G	1.91379E-51	TYG
135	rs268	0.10865	0.00515243	G	A	1.25248E-98	TYG
136	rs301	-0.0598103	0.00161337	C	T	4.5014E-300	TYG
137	rs2081687	0.011677	0.00145382	T	C	9.63108E-16	TYG
138	rs11558471	-0.011475	0.00147145	G	A	6.29222E-15	TYG
139	rs2954029	-0.0437087	0.00137736	T	A	1.3519E-220	TYG
140	rs10811661	-0.0098734	0.0018054	C	T	4.53471E-08	TYG
141	rs1832007	-0.0122404	0.00189787	G	A	1.12354E-10	TYG
142	rs2068888	-0.0157168	0.00137845	A	G	4.15948E-30	TYG
143	rs2792751	-0.0100993	0.00153887	T	C	5.28969E-11	TYG
144	rs2167079	-0.009738	0.00148936	T	C	6.2299E-11	TYG
145	rs174546	0.0199228	0.00144018	T	C	1.65448E-43	TYG
146	rs174576	0.019814	0.00143578	A	C	2.62854E-43	TYG
147	rs35169799	0.0247241	0.00283283	T	C	2.61306E-18	TYG
148	rs7350481	0.124543	0.00296014	T	C	0	TYG
149	rs3135506	0.114697	0.00285743	C	G	0	TYG
150	rs4149056	0.0126505	0.00191707	C	T	4.15004E-11	TYG
151	rs1106766	-0.0159069	0.00159257	T	C	1.73266E-23	TYG
152	rs10861661	0.00947809	0.00159299	C	A	2.68678E-09	TYG
153	rs11057401	-0.0132748	0.00147944	A	T	2.90774E-19	TYG
154	rs55707100	0.0705316	0.00441895	T	C	2.5295E-57	TYG
155	rs1532085	0.0180035	0.0014104	A	G	2.64155E-37	TYG
156	rs1800588	0.0257794	0.00167039	T	C	1.03161E-53	TYG
157	rs7200543	0.0118871	0.00150255	G	A	2.55692E-15	TYG
158	rs247616	-0.018572	0.00146483	T	C	7.9525E-37	TYG
159	rs5880	0.0221143	0.00298074	C	G	1.18303E-13	TYG
160	rs2000999	0.0136007	0.00176221	A	G	1.18587E-14	TYG
161	rs2925979	0.0154375	0.00149811	T	C	6.78754E-25	TYG
162	rs72836561	0.0682178	0.00389919	T	C	1.69357E-68	TYG
163	rs12453522	0.0120865	0.00183913	G	A	4.97779E-11	TYG
164	rs1801689	-0.0293183	0.00408097	C	A	6.77966E-13	TYG
165	rs2292642	0.00854886	0.00140345	C	T	1.1213E-09	TYG
166	rs116843064	-0.108878	0.00491157	A	G	8.7939E-109	TYG
167	rs58542926	-0.0520456	0.00257716	T	C	1.26346E-90	TYG
168	rs731839	0.0104226	0.00145663	G	A	8.37274E-13	TYG
169	rs28399654	0.027563	0.00375771	A	G	2.22252E-13	TYG
170	rs439401	-0.038144	0.00142601	T	C	2.0361E-157	TYG
171	rs2287922	-0.0095132	0.00137968	G	A	5.38933E-12	TYG
172	rs7679	0.0273082	0.00176281	C	T	4.19262E-54	TYG
173	rs738322	-0.008272	0.00137428	G	A	1.75588E-09	TYG

Table S4. Baseline characteristics of participants by 2×2 factorial groups in dataset A.

Characteristics	2×2 Group, Mean (SD)				P value
	Reference (Both Genetic Scores ≤ Median)	Genetically Higher GLU (GLU Genetic Score > Median; log-TG Genetic Score ≤ Median)	Genetically Higher log-TG(log-TG Genetic Score > Median; GLU Genetic Score ≤ Median)	Both Genetically Higher GLU and Higher log-TG (Both Genetic Scores > Median)	
**Age, years**	56.00±8.02	55.85±8.05	55.75±8.08	55.69±8.06	<0.001
**BMI, kg/m^2**	26.95±4.55	26.91±4.53	26.83±4.48	26.81±4.44	<0.001
**TC, mmol/L**	5.78±1.02	5.78±1.02	5.92±1.09	5.93±1.09	<0.001
**TG, mmol/L**	1.47±0.77	1.48±0.78	1.84±1.01	1.85±1.01	<0.001
**log TG, log mmol/l**	0.27±0.47	0.27±0.48	0.48±0.50	0.48±0.50	<0.001
**HDL-C, mmol/l**	1.53±0.38	1.53±0.38	1.45±0.37	1.45±0.37	<0.001
**LDL-C, mmol/l**	3.62±0.79	3.62±0.79	3.73±0.83	3.74±0.83	<0.001
**SBP, mm Hg**	138.88±19.61	138.88±19.59	138.65±19.31	138.94±19.52	0.044
**DBP, mm Hg**	82.14±10.70	81.95±10.66	82.09±10.60	82.08±10.66	0.015
**GLU, mmol/l**	4.87±0.61	5.02±0.62	4.86±0.61	5.01±0.62	<0.001
**HbA1c, mmol/l**	34.31±3.67	35.03±3.78	34.23±4.16	34.98±3.70	<0.001
**TyG index**	8.53±0.50	8.56±0.50	8.73±0.53	8.77±0.53	<0.001
**Fasting time, hours**	3.74±2.39	3.74±2.37	3.74±2.39	3.75±2.38	0.924
**Sex, No. (%)**					0.115
Female	38787(56.99)	39302(57.27)	39436(57.47)	39204(57.60)	
Male	29277(43.01)	29318(42.73)	29184(42.53)	28860(42.40)	
**Smoke, No. (%)**					0.326
Never	38816(57.22)	38962(56.93)	39340(57.50)	38920(57.36)	
Previous	22451(33.10)	22739(33.23)	22368(32.69)	22267(32.82)	
Current	6569(9.68)	6736(9.84)	6713(9.81)	6664(9.82)	
**Drink, No (%)**					0.397
Never	2022(2.97)	1923(2.80)	1982(2.89)	1990(2.93)	
Previous	2076(3.05)	2008(2.93)	2064(3.01)	2073(3.05)	
Current	63917(93.97)	64648(94.27)	64522(94.10)	63935(94.02)	

Table S5. Baseline characteristics of participants by 2×2 factorial groups in dataset B.

Characteristics	2×2 Group, Mean (SD)				P value
	Reference (Both Genetic Scores ≤ Median)	Genetically Higher GLU (GLU Genetic Score > Median; log-TG Genetic Score ≤ Median)	Genetically Higher log-TG(log-TG Genetic Score > Median; GLU Genetic Score ≤ Median)	Both Genetically Higher GLU and Higher log-TG (Both Genetic Scores > Median)	
**Age, years**	56.96±7.98	56.81±8.02	56.90±8.01	56.92±8.01	0.001
**BMI, kg/m^2**	27.36±4.77	27.35±4.71	27.37±4.68	27.34±4.64	0.587
**TC, mmol/L**	5.69±1.10	5.69±1.11	5.77±1.16	5.77±1.17	<0.001
**TG, mmol/L**	1.56±0.84	1.57±0.85	1.89±1.03	1.90±1.04	<0.001
**log TG, log mmol/l**	0.32±0.49	0.33±0.49	0.51±0.51	0.51±0.51	<0.001
**HDL-C, mmol/l**	1.50±0.39	1.50±0.39	1.42±0.37	1.42±0.37	<0.001
**LDL-C, mmol/l**	3.54±0.84	3.54±0.85	3.63±0.88	3.63±0.89	<0.001
**SBP, mm Hg**	139.92±19.60	140.00±19.65	140.27±19.62	140.47±19.66	<0.001
**DBP, mm Hg**	82.29±10.64	82.13±10.63	82.40±10.66	82.33±10.66	<0.001
**GLU, mmol/l**	4.94±0.68	5.06±0.70	4.95±0.69	5.07±0.72	<0.001
**HbA1c, mmol/l**	35.13±5.00	35.76±4.93	35.22±4.92	35.92±5.21	<0.001
**TyG index**	8.59±0.52	8.62±0.52	8.78±0.54	8.81±0.54	<0.001
**Fasting time, hours**	3.76±2.38	3.78±2.41	3.76±2.35	3.78±2.39	0.026
**Sex, No. (%)**					0.292
Female	46824(54.25)	47053(53.94)	47189(54.10)	46448(53.82)	
Male	39484(45.75)	40177(46.06)	40041(45.90)	39860(46.18)	
**DM, No. (%)**					<0.001
No	82258(95.31)	82229(94.27)	82557(94.64)	80551(93.33)	
Yes	4050(4.69)	5001(5.73)	4673(5.36)	5757(6.67)	
**DLM, No. (%)**					<0.001
No	72016(83.44)	72672(83.31)	69531(79.71)	68223(79.05)	
Yes	14292(16.56)	14558(16.69)	17699(20.29)	18085(20.95)	
**Smoke, No. (%)**					0.144
Never	47242(54.92)	47351(54.46)	47410(54.55)	47165(54.83)	
Previous	30182(35.09)	30621(35.22)	30569(35.18)	30204(35.12)	
Current	8590(9.99)	8979(10.33)	8926(10.27)	8645(10.05)	
**Drink, No (%)**					0.2
Never	2681(3.11)	2691(3.09)	2669(3.06)	2734(3.17)	
Previous	2816(3.27)	2928(3.36)	3028(3.47)	2842(3.30)	
Current	80743(93.63)	81538(93.55)	81456(93.46)	80647(93.53)	

Table S6. MR-egger test for potential horizontal pleiotropic.

Exposure	Outcome	Intercept	Intercept. P value	Beta	Beta. P value
TG	CIHD	3.0864E-05	0.991732104	0.36125835	0.01851754
TG	CVD	0.00180128	0.356001968	0.14234473	0.1547231
TG	IS	-0.0015649	0.770227693	0.40957005	0.13602321
TG	CED	0.003529	0.284498693	-0.0788192	0.64032799
TG	AP	-0.0008571	0.778789586	0.36160901	0.02136737
TG	AMI	-0.0034734	0.33267875	0.69886574	0.00017009
TG	HS	0.00656659	0.29560799	-0.2381102	0.45992285
TG	HF	-0.0050776	0.283897964	0.29170463	0.22760377
TG	IHD	0.00032439	0.8940705	0.28753496	0.02195141
GLU	IHD	-0.0064363	0.079815596	0.57496199	0.00110093
GLU	HF	0.01306181	0.040739066	-0.433967	0.14253532
GLU	CED	-0.0001816	0.968385449	-0.0480336	0.82239484
GLU	IS	0.01785204	0.03664271	-0.7570544	0.05684163
GLU	CIHD	-0.0090155	0.055432179	0.78119302	0.00056263
GLU	AP	-0.0075345	0.095000124	0.49166678	0.02075182
GLU	CVD	-0.0041085	0.142205816	0.37254235	0.00519515
GLU	HS	-0.0092702	0.280097362	0.51821349	0.19605438
GLU	AMI	-0.0055513	0.403855341	0.67625288	0.03183475
TYG	HS	0.00302644	0.633357676	-0.095691	0.75878738
TYG	IS	-0.0053631	0.369294603	0.44485788	0.13049643
TYG	IHD	5.6328E-05	0.984644889	0.26718249	0.06409977
TYG	AMI	-0.0050776	0.255778202	0.5935294	0.00699687
TYG	CIHD	-0.001184	0.74926765	0.33192703	0.0689957
TYG	HF	-0.0088394	0.06779737	0.41315274	0.08116945
TYG	CED	0.00043876	0.898813822	0.00510985	0.97594748
TYG	AP	-5.15E-05	0.988248897	0.38553822	0.02561126
TYG	CVD	0.00054079	0.817240803	0.17703633	0.1245364

Table S7. Subgroup analysis for combined exposure to GLU and TG.

No.	Subgroup	Control	Case	OR(95%CI)	P for interaction	Outcome
1	Sex					CVD
2	Male	149846	6883	1.03(0.96-1.10)	>0.05	CVD
3	Female	106975	9664	1.11(1.04-1.17)		CVD
4	Age					CVD
5	≤median	139741	4745	1.14(1.05-1.24)	>0.05	CVD
6	>median	117080	11802	1.06(1.01-1.12)		CVD
7	BMI					CVD
8	≤median	129787	6533	1.08(1.01-1.16)	>0.05	CVD
9	>median	126407	9900	1.07(1.01-1.13)		CVD
10	Fasting time					CVD
11	≤median	144955	8540	1.08(1.01-1.15)	>0.05	CVD
12	>median	111857	8007	1.05(0.99-1.12)		CVD
13	HDL-C					CVD
14	≤median	126383	10416	1.04(0.98-1.10)	>0.05	CVD
15	>median	130384	6131	0.99(0.92-1.07)		CVD
16	LDL-C					CVD
17	≤median	126931	9608	1.17(1.10-1.24)	0.000	CVD
18	>median	129527	6916	0.99(0.93-1.06)		CVD
19	HbA1c					CVD
20	≤median	125362	6315	1.03(0.96-1.11)	>0.05	CVD
21	>median	119846	9516	1.03(0.97-1.10)		CVD
22	SBP					CVD
23	≤median	123129	6268	1.10(1.03-1.19)	>0.05	CVD
24	>median	116137	8960	1.05(0.99-1.12)		CVD
25	DBP					CVD
26	≤median	128232	7636	1.13(1.06-1.21)	0.014	CVD
27	>median	111038	7592	1.02(0.95-1.09)		CVD
28	Smoke status					CVD
29	No	148394	7644	1.12(1.05-1.19)	0.026	CVD
30	Yes	107690	8817	1.02(0.96-1.09)		CVD
31	Sex					IHD
32	Male	152459	4270	1.08(0.99-1.17)	>0.05	IHD
33	Female	109871	6768	1.14(1.06-1.22)		IHD
34	Age					IHD
35	≤median	141531	2955	1.16(1.05-1.28)	>0.05	IHD
36	>median	120799	8083	1.11(1.05-1.19)		IHD
37	BMI					IHD
38	≤median	132205	4115	1.11(1.01-1.21)	>0.05	IHD
39	>median	129444	6863	1.12(1.05-1.20)		IHD
40	Fasting time					IHD
41	≤median	147789	5706	1.11(1.03-1.20)	>0.05	IHD
42	>median	114532	5332	1.10(1.02-1.19)		IHD

No.	Subgroup	Control	Case	OR(95%CI)	P for interaction	Outcome
43	HDL-C					IHD
44	≤median	129524	7275	1.08(1.01-1.15)	>0.05	IHD
45	>median	132752	3763	1.00(0.92-1.10)		IHD
46	LDL-C					IHD
47	≤median	130058	6481	1.22(1.14-1.31)	0.000	IHD
48	>median	131905	4538	1.02(0.94-1.11)		IHD
49	HbA1c					IHD
50	≤median	127623	4054	1.09(1.00-1.20)	>0.05	IHD
51	>median	122840	6522	1.05(0.98-1.12)		IHD
52	SBP					IHD
53	≤median	125233	4164	1.14(1.04-1.24)	>0.05	IHD
54	>median	119093	6004	1.11(1.03-1.19)		IHD
55	DBP					IHD
56	≤median	130655	5213	1.17(1.08-1.27)	>0.05	IHD
57	>median	113675	4955	1.07(0.99-1.16)		IHD
58	Smoke status					IHD
59	No	151047	4991	1.13(1.05-1.23)	>0.05	IHD
60	Yes	110521	5986	1.09(1.01-1.17)		IHD
61	Sex					CED
62	Male	154283	2446	0.98(0.87-1.10)	>0.05	CED
63	Female	113871	2768	1.01(0.91-1.12)		CED
64	Age					CED
65	≤median	142847	1639	1.08(0.94-1.24)	>0.05	CED
66	>median	125307	3575	0.97(0.88-1.07)		CED
67	BMI					CED
68	≤median	134026	2294	1.00(0.89-1.12)	>0.05	CED
69	>median	133436	2871	1.00(0.90-1.11)		CED
70	Fasting time					CED
71	≤median	150846	2649	1.00(0.89-1.11)	>0.05	CED
72	>median	117299	2565	0.99(0.89-1.10)		CED
73	HDL-C					CED
74	≤median	133775	3024	0.96(0.86-1.06)	>0.05	CED
75	>median	134325	2190	0.98(0.87-1.11)		CED
76	LDL-C					CED
77	≤median	133478	3061	1.07(0.97-1.19)	0.030	CED
78	>median	134297	2146	0.94(0.83-1.06)		CED
79	HbA1c					CED
80	≤median	129594	2083	0.95(0.84-1.07)	>0.05	CED
81	>median	126465	2897	1.00(0.90-1.11)		CED
82	SBP					CED
83	≤median	127442	1955	1.02(0.90-1.16)	>0.05	CED
84	>median	122276	2821	0.99(0.89-1.10)		CED
85	DBP					CED

No.	Subgroup	Control	Case	OR(95%CI)	P for interaction	Outcome
86	≤median	133574	2294	1.06(0.94-1.19)	>0.05	CED
87	>median	116148	2482	0.95(0.85-1.06)		CED
88	Smoke status					CED
89	No	153600	2438	1.04(0.93-1.17)	>0.05	CED
90	Yes	113759	2748	0.95(0.85-1.06)		CED
91	Sex					AP
92	Male	154184	2545	1.11(0.99-1.24)	>0.05	AP
93	Female	113268	3371	1.10(1.00-1.22)		AP
94	Age					AP
95	≤median	142980	1506	1.22(1.05-1.40)	>0.05	AP
96	>median	124472	4410	1.09(1.00-1.19)		AP
97	BMI					AP
98	≤median	134229	2091	1.11(0.98-1.25)	>0.05	AP
99	>median	132524	3783	1.12(1.02-1.22)		AP
100	Fasting time					AP
101	≤median	150451	3044	1.15(1.04-1.27)	>0.05	AP
102	>median	116992	2872	1.05(0.95-1.17)		AP
103	HDL-C					AP
104	≤median	132848	3951	1.07(0.98-1.17)	>0.05	AP
105	>median	134550	1965	0.99(0.87-1.13)		AP
106	LDL-C					AP
107	≤median	132857	3682	1.24(1.12-1.36)	0.001	AP
108	>median	134218	2225	0.99(0.89-1.12)		AP
109	HbA1c					AP
110	≤median	129587	2090	1.12(0.99-1.27)	>0.05	AP
111	>median	125804	3558	1.02(0.93-1.12)		AP
112	SBP					AP
113	≤median	127081	2316	1.17(1.04-1.32)	>0.05	AP
114	>median	121977	3120	1.08(0.97-1.19)		AP
115	DBP					AP
116	≤median	132923	2945	1.18(1.07-1.32)	0.042	AP
117	>median	116139	2491	1.04(0.93-1.16)		AP
118	Smoke status					AP
119	No	153360	2678	1.12(1.01-1.25)	>0.05	AP
120	Yes	113302	3205	1.08(0.98-1.20)		AP
121	Sex					AMI
122	Male	155645	1084	1.12(0.95-1.32)	>0.05	AMI
123	Female	113686	2953	1.29(1.16-1.43)		AMI
124	Age					AMI
125	≤median	143349	1137	1.34(1.13-1.58)	>0.05	AMI
126	>median	125982	2900	1.22(1.10-1.35)		AMI
127	BMI					AMI
128	≤median	134779	1541	1.15(1.00-1.33)	>0.05	AMI

No.	Subgroup	Control	Case	OR(95%CI)	P for interaction	Outcome
129	>median	133832	2475	1.30(1.16-1.45)		AMI
130	Fasting time					AMI
131	≤median	151409	2086	1.21(1.07-1.37)	>0.05	AMI
132	>median	117913	1951	1.25(1.10-1.42)		AMI
133	HDL-C					AMI
134	≤median	133882	2917	1.19(1.07-1.32)	>0.05	AMI
135	>median	135395	1120	1.06(0.90-1.25)		AMI
136	LDL-C					AMI
137	≤median	134007	2532	1.35(1.21-1.51)	0.026	AMI
138	>median	134941	1502	1.15(1.00-1.33)		AMI
139	HbA1c					AMI
140	≤median	130278	1399	1.22(1.06-1.42)	>0.05	AMI
141	>median	126873	2489	1.13(1.01-1.26)		AMI
142	SBP					AMI
143	≤median	127874	1523	1.29(1.12-1.49)	>0.05	AMI
144	>median	122941	2156	1.23(1.09-1.38)		AMI
145	DBP					AMI
146	≤median	133964	1904	1.25(1.10-1.42)	>0.05	AMI
147	>median	116855	1775	1.26(1.10-1.44)		AMI
148	Smoke status					AMI
149	No	154379	1659	1.21(1.05-1.38)	>0.05	AMI
150	Yes	114152	2355	1.26(1.12-1.41)		AMI
151	Sex					CIHD
152	Male	154222	2507	1.09(0.97-1.22)	>0.05	CIHD
153	Female	111620	5019	1.14(1.05-1.24)		CIHD
154	Age					CIHD
155	≤median	142605	1881	1.19(1.05-1.35)	>0.05	CIHD
156	>median	123237	5645	1.12(1.04-1.21)		CIHD
157	BMI					CIHD
158	≤median	133484	2836	1.12(1.01-1.24)	>0.05	CIHD
159	>median	131654	4653	1.12(1.03-1.22)		CIHD
160	Fasting time					CIHD
161	≤median	149596	3899	1.11(1.02-1.22)	>0.05	CIHD
162	>median	116237	3627	1.12(1.02-1.22)		CIHD
163	HDL-C					CIHD
164	≤median	131668	5131	1.09(1.00-1.18)	0.047	CIHD
165	>median	134120	2395	0.99(0.88-1.11)		CIHD
166	LDL-C					CIHD
167	≤median	132103	4436	1.25(1.15-1.36)	0.000	CIHD
168	>median	133370	3073	1.01(0.91-1.11)		CIHD
169	HbA1c					CIHD
170	≤median	128927	2750	1.08(0.97-1.20)	>0.05	CIHD
171	>median	124890	4472	1.08(0.99-1.17)		CIHD

No.	Subgroup	Control	Case	OR(95%CI)	P for interaction	Outcome
172	SBP					CIHD
173	≤median	126666	2731	1.16(1.04-1.29)	>0.05	CIHD
174	>median	120905	4192	1.11(1.01-1.21)		CIHD
175	DBP					CIHD
176	≤median	132357	3511	1.17(1.07-1.29)	>0.05	CIHD
177	>median	115218	3412	1.08(0.98-1.19)		CIHD
178	Smoke status					CIHD
179	No	152736	3302	1.11(1.01-1.22)	>0.05	CIHD
180	Yes	112325	4182	1.13(1.03-1.23)		CIHD
181	Sex					HF
182	Male	155925	804	0.91(0.75-1.10)	0.040	HF
183	Female	115232	1407	1.16(1.00-1.35)		HF
184	Age					HF
185	≤median	143971	515	1.19(0.93-1.51)	>0.05	HF
186	>median	127186	1696	1.04(0.91-1.19)		HF
187	BMI					HF
188	≤median	135475	845	1.11(0.92-1.34)	>0.05	HF
189	>median	134968	1339	1.04(0.89-1.21)		HF
190	Fasting time					HF
191	≤median	152415	1080	1.15(0.97-1.36)	>0.05	HF
192	>median	118733	1131	0.97(0.82-1.14)		HF
193	HDL-C					HF
194	≤median	135408	1391	1.03(0.89-1.20)	>0.05	HF
195	>median	135695	820	0.98(0.80-1.19)		HF
196	LDL-C					HF
197	≤median	135246	1293	1.15(0.98-1.34)	>0.05	HF
198	>median	135526	917	0.98(0.82-1.17)		HF
199	HbA1c					HF
200	≤median	130890	787	0.93(0.76-1.13)	>0.05	HF
201	>median	128029	1333	1.04(0.89-1.21)		HF
202	SBP					HF
203	≤median	128569	828	1.05(0.86-1.27)	>0.05	HF
204	>median	123895	1202	1.00(0.85-1.17)		HF
205	DBP					HF
206	≤median	134879	989	1.06(0.89-1.27)	>0.05	HF
207	>median	117589	1041	0.98(0.82-1.16)		HF
208	Smoke status					HF
209	No	155069	969	1.18(0.99-1.41)	>0.05	HF
210	Yes	115276	1231	0.97(0.83-1.14)		HF
211	Sex					HS
212	Male	156041	688	1.05(0.85-1.29)	>0.05	HS
213	Female	116005	634	0.90(0.73-1.12)		HS
214	Age					HS

No.	Subgroup	Control	Case	OR(95%CI)	P for interaction	Outcome
215	≤median	143960	526	1.00(0.79-1.27)	>0.05	HS
216	>median	128086	796	0.97(0.80-1.18)		HS
217	BMI					HS
218	≤median	135656	664	0.95(0.77-1.17)	>0.05	HS
219	>median	135658	649	0.98(0.78-1.22)		HS
220	Fasting time					HS
221	≤median	152808	687	0.94(0.76-1.16)	>0.05	HS
222	>median	119229	635	1.01(0.82-1.25)		HS
223	HDL-C					HS
224	≤median	136113	686	1.02(0.82-1.26)	>0.05	HS
225	>median	135879	636	0.92(0.74-1.15)		HS
226	LDL-C					HS
227	≤median	135845	694	1.00(0.81-1.23)	>0.05	HS
228	>median	135817	626	0.95(0.76-1.19)		HS
229	HbA1c					HS
230	≤median	131109	568	0.83(0.66-1.04)	0.042	HS
231	>median	128671	691	1.10(0.89-1.36)		HS
232	SBP					HS
233	≤median	128884	513	1.23(0.97-1.57)	0.029	HS
234	>median	124400	697	0.89(0.72-1.09)		HS
235	DBP					HS
236	≤median	135278	590	1.07(0.85-1.34)	>0.05	HS
237	>median	118010	620	0.98(0.79-1.22)		HS
238	Smoke status					HS
239	No	155424	614	0.92(0.74-1.15)	>0.05	HS
240	Yes	115802	705	1.03(0.84-1.27)		HS
241	Sex					IS
242	Male	156052	677	1.00(0.81-1.24)	>0.05	IS
243	Female	115620	1019	1.07(0.90-1.27)		IS
244	Age					IS
245	≤median	144028	458	1.15(0.89-1.49)	>0.05	IS
246	>median	127644	1238	1.02(0.87-1.19)		IS
247	BMI					IS
248	≤median	135583	737	0.95(0.77-1.18)	>0.05	IS
249	>median	135359	948	1.11(0.93-1.33)		IS
250	Fasting time					IS
251	≤median	152604	891	0.97(0.80-1.17)	>0.05	IS
252	>median	119059	805	1.12(0.92-1.36)		IS
253	HDL-C					IS
254	≤median	135752	1047	0.98(0.83-1.17)	>0.05	IS
255	>median	135866	649	1.02(0.82-1.27)		IS
256	LDL-C					IS
257	≤median	135596	943	1.14(0.95-1.36)	>0.05	IS

No.	Subgroup	Control	Case	OR(95%CI)	P for interaction	Outcome
258	>median	135692	751	0.95(0.78-1.16)		IS
259	HbA1c					IS
260	≤median	131026	651	1.07(0.85-1.34)	>0.05	IS
261	>median	128390	972	0.97(0.82-1.16)		IS
262	SBP					IS
263	≤median	128845	552	1.05(0.83-1.34)	>0.05	IS
264	>median	124114	983	1.08(0.90-1.29)		IS
265	DBP					IS
266	≤median	135176	692	1.09(0.88-1.36)	>0.05	IS
267	>median	117787	843	1.05(0.87-1.27)		IS
268	Smoke status					IS
269	No	155278	760	1.15(0.94-1.42)	>0.05	IS
270	Yes	115581	926	0.95(0.79-1.14)		IS

Table S8. Subgroup analysis for TyG index.

No.	Subgroup	Control	Case	OR(95%CI)	P for interaction	Outcome
1	Sex					CVD
2	Male	149846	6883	1.06(0.99-1.13)	>0.05	CVD
3	Female	106975	9664	1.08(1.02-1.14)		CVD
4	Age					CVD
5	≤median	139741	4745	1.12(1.03-1.21)	>0.05	CVD
6	>median	117080	11802	1.07(1.01-1.13)		CVD
7	BMI					CVD
8	≤median	129787	6533	1.12(1.05-1.21)	0.048	CVD
9	>median	126407	9900	1.03(0.97-1.09)		CVD
10	Fasting time					CVD
11	≤median	144955	8540	1.08(1.01-1.15)	>0.05	CVD
12	>median	111857	8007	1.05(0.98-1.11)		CVD
13	HDL-C					CVD
14	≤median	126383	10416	0.97(0.92-1.03)	>0.05	CVD
15	>median	130384	6131	0.99(0.92-1.07)		CVD
16	LDL-C					CVD
17	≤median	126931	9608	1.20(1.13-1.27)	0.000	CVD
18	>median	129527	6916	0.98(0.91-1.05)		CVD
19	HbA1c					CVD
20	≤median	125362	6315	1.13(1.05-1.22)	0.034	CVD
21	>median	119846	9516	1.03(0.97-1.09)		CVD
22	SBP					CVD
23	≤median	123129	6268	1.06(0.99-1.14)	>0.05	CVD
24	>median	116137	8960	1.07(1.01-1.14)		CVD
25	DBP					CVD
26	≤median	128232	7636	1.09(1.02-1.16)	>0.05	CVD
27	>median	111038	7592	1.05(0.98-1.12)		CVD
28	Smoke status					CVD
29	No	148394	7644	1.11(1.04-1.19)	0.040	CVD
30	Yes	107690	8817	1.02(0.96-1.09)		CVD
31	Sex					IHD
32	Male	152459	4270	1.08(0.99-1.17)	>0.05	IHD
33	Female	109871	6768	1.09(1.02-1.17)		IHD
34	Age					IHD
35	≤median	141531	2955	1.16(1.04-1.29)	>0.05	IHD
36	>median	120799	8083	1.08(1.01-1.15)		IHD
37	BMI					IHD
38	≤median	132205	4115	1.15(1.05-1.26)	>0.05	IHD
39	>median	129444	6863	1.05(0.98-1.12)		IHD
40	Fasting time					IHD
41	≤median	147789	5706	1.12(1.04-1.20)	>0.05	IHD

42	>median	114532	5332	1.04(0.96-1.12)		IHD
43	HDL-C					IHD
44	≤median	129524	7275	0.98(0.92-1.05)	>0.05	IHD
45	>median	132752	3763	0.98(0.90-1.08)		IHD
46	LDL-C					IHD
47	≤median	130058	6481	1.23(1.14-1.31)	0.000	IHD
48	>median	131905	4538	0.98(0.90-1.07)		IHD
49	HbA1c					IHD
50	≤median	127623	4054	1.15(1.05-1.25)	>0.05	IHD
51	>median	122840	6522	1.05(0.98-1.13)		IHD
52	SBP					IHD
53	≤median	125233	4164	1.09(1.00-1.19)	>0.05	IHD
54	>median	119093	6004	1.07(0.99-1.15)		IHD
55	DBP					IHD
56	≤median	130655	5213	1.07(0.99-1.16)	>0.05	IHD
57	>median	113675	4955	1.08(1.00-1.17)		IHD
58	Smoke status					IHD
59	No	151047	4991	1.07(0.99-1.16)	>0.05	IHD
60	Yes	110521	5986	1.08(1.01-1.17)		IHD
61	Sex					CED
62	Male	154283	2446	1.06(0.95-1.19)	>0.05	CED
63	Female	113871	2768	1.01(0.91-1.12)		CED
64	Age					CED
65	≤median	142847	1639	1.09(0.95-1.25)	>0.05	CED
66	>median	125307	3575	1.02(0.93-1.13)		CED
67	BMI					CED
68	≤median	134026	2294	1.10(0.98-1.24)	>0.05	CED
69	>median	133436	2871	0.99(0.89-1.10)		CED
70	Fasting time					CED
71	≤median	150846	2649	0.98(0.88-1.09)	>0.05	CED
72	>median	117299	2565	1.09(0.97-1.22)		CED
73	HDL-C					CED
74	≤median	133775	3024	0.94(0.85-1.04)	>0.05	CED
75	>median	134325	2190	1.04(0.92-1.17)		CED
76	LDL-C					CED
77	≤median	133478	3061	1.14(1.03-1.27)	0.010	CED
78	>median	134297	2146	0.96(0.85-1.09)		CED
79	HbA1c					CED
80	≤median	129594	2083	1.13(1.00-1.28)	0.046	CED
81	>median	126465	2897	0.98(0.88-1.08)		CED
82	SBP					CED
83	≤median	127442	1955	1.03(0.91-1.17)	>0.05	CED
84	>median	122276	2821	1.07(0.96-1.19)		CED
85	DBP					CED

86	≤median	133574	2294	1.15(1.02-1.29)	0.024	CED
87	>median	116148	2482	0.97(0.87-1.09)		CED
88	Smoke status					CED
89	No	153600	2438	1.11(0.99-1.24)	0.046	CED
90	Yes	113759	2748	0.97(0.87-1.08)		CED
91	Sex					AP
92	Male	154184	2545	1.08(0.97-1.21)	>0.05	AP
93	Female	113268	3371	1.15(1.05-1.27)		AP
94	Age					AP
95	≤median	142980	1506	1.16(1.00-1.34)	>0.05	AP
96	>median	124472	4410	1.13(1.04-1.23)		AP
97	BMI					AP
98	≤median	134229	2091	1.16(1.03-1.31)	>0.05	AP
99	>median	132524	3783	1.11(1.01-1.21)		AP
100	Fasting time					AP
101	≤median	150451	3044	1.18(1.07-1.31)	>0.05	AP
102	>median	116992	2872	1.05(0.95-1.17)		AP
103	HDL-C					AP
104	≤median	132848	3951	1.05(0.96-1.15)	>0.05	AP
105	>median	134550	1965	0.97(0.85-1.10)		AP
106	LDL-C					AP
107	≤median	132857	3682	1.31(1.19-1.43)	0.000	AP
108	>median	134218	2225	0.98(0.87-1.10)		AP
109	HbA1c					AP
110	≤median	129587	2090	1.17(1.03-1.32)	>0.05	AP
111	>median	125804	3558	1.09(0.99-1.20)		AP
112	SBP					AP
113	≤median	127081	2316	1.14(1.01-1.28)	>0.05	AP
114	>median	121977	3120	1.10(1.00-1.22)		AP
115	DBP					AP
116	≤median	132923	2945	1.13(1.02-1.25)	>0.05	AP
117	>median	116139	2491	1.11(0.99-1.24)		AP
118	Smoke status					AP
119	No	153360	2678	1.13(1.01-1.25)	>0.05	AP
120	Yes	113302	3205	1.11(1.01-1.23)		AP
121	Sex					AMI
122	Male	155645	1084	1.18(1.00-1.40)	>0.05	AMI
123	Female	113686	2953	1.12(1.01-1.24)		AMI
124	Age					AMI
125	≤median	143349	1137	1.30(1.09-1.54)	>0.05	AMI
126	>median	125982	2900	1.09(0.98-1.21)		AMI
127	BMI					AMI
128	≤median	134779	1541	1.21(1.05-1.40)	>0.05	AMI
129	>median	133832	2475	1.08(0.96-1.21)		AMI

130	Fasting time					AMI
131	≤median	151409	2086	1.16(1.03-1.31)	>0.05	AMI
132	>median	117913	1951	1.08(0.95-1.23)		AMI
133	HDL-C					AMI
134	≤median	133882	2917	0.97(0.87-1.08)	>0.05	AMI
135	>median	135395	1120	1.03(0.87-1.22)		AMI
136	LDL-C					AMI
137	≤median	134007	2532	1.31(1.17-1.46)	0.000	AMI
138	>median	134941	1502	0.99(0.85-1.15)		AMI
139	HbA1c					AMI
140	≤median	130278	1399	1.25(1.08-1.45)	>0.05	AMI
141	>median	126873	2489	1.09(0.97-1.22)		AMI
142	SBP					AMI
143	≤median	127874	1523	1.17(1.01-1.35)	>0.05	AMI
144	>median	122941	2156	1.09(0.97-1.24)		AMI
145	DBP					AMI
146	≤median	133964	1904	1.11(0.98-1.26)	>0.05	AMI
147	>median	116855	1775	1.15(1.00-1.31)		AMI
148	Smoke status					AMI
149	No	154379	1659	1.08(0.95-1.24)	>0.05	AMI
150	Yes	114152	2355	1.16(1.03-1.30)		AMI
151	Sex					CIHD
152	Male	154222	2507	1.07(0.96-1.20)	>0.05	CIHD
153	Female	111620	5019	1.10(1.01-1.19)		CIHD
154	Age					CIHD
155	≤median	142605	1881	1.17(1.02-1.33)	>0.05	CIHD
156	>median	123237	5645	1.08(1.00-1.16)		CIHD
157	BMI					CIHD
158	≤median	133484	2836	1.13(1.01-1.25)	>0.05	CIHD
159	>median	131654	4653	1.06(0.97-1.15)		CIHD
160	Fasting time					CIHD
161	≤median	149596	3899	1.11(1.02-1.22)	>0.05	CIHD
162	>median	116237	3627	1.04(0.95-1.14)		CIHD
163	HDL-C					CIHD
164	≤median	131668	5131	0.97(0.90-1.05)	>0.05	CIHD
165	>median	134120	2395	0.97(0.86-1.08)		CIHD
166	LDL-C					CIHD
167	≤median	132103	4436	1.23(1.13-1.34)	0.000	CIHD
168	>median	133370	3073	0.98(0.88-1.08)		CIHD
169	HbA1c					CIHD
170	≤median	128927	2750	1.17(1.05-1.31)	>0.05	CIHD
171	>median	124890	4472	1.05(0.96-1.14)		CIHD
172	SBP					CIHD
173	≤median	126666	2731	1.07(0.96-1.20)	>0.05	CIHD

174	>median	120905	4192	1.08(0.99-1.18)		CIHD
175	DBP					CIHD
176	≤median	132357	3511	1.05(0.96-1.16)	>0.05	CIHD
177	>median	115218	3412	1.10(1.00-1.22)		CIHD
178	Smoke status					CIHD
179	No	152736	3302	1.06(0.96-1.17)	>0.05	CIHD
180	Yes	112325	4182	1.09(1.00-1.19)		CIHD
181	Sex					HF
182	Male	155925	804	0.98(0.80-1.19)	>0.05	HF
183	Female	115232	1407	1.00(0.86-1.16)		HF
184	Age					HF
185	≤median	143971	515	0.85(0.65-1.09)	>0.05	HF
186	>median	127186	1696	1.05(0.92-1.21)		HF
187	BMI					HF
188	≤median	135475	845	1.06(0.88-1.29)	>0.05	HF
189	>median	134968	1339	0.95(0.82-1.11)		HF
190	Fasting time					HF
191	≤median	152415	1080	1.06(0.90-1.26)	>0.05	HF
192	>median	118733	1131	0.91(0.77-1.08)		HF
193	HDL-C					HF
194	≤median	135408	1391	0.86(0.74-0.99)	0.050	HF
195	>median	135695	820	1.02(0.84-1.25)		HF
196	LDL-C					HF
197	≤median	135246	1293	1.06(0.91-1.24)	>0.05	HF
198	>median	135526	917	0.97(0.80-1.17)		HF
199	HbA1c					HF
200	≤median	130890	787	0.90(0.74-1.10)	>0.05	HF
201	>median	128029	1333	1.04(0.89-1.21)		HF
202	SBP					HF
203	≤median	128569	828	0.87(0.71-1.05)	>0.05	HF
204	>median	123895	1202	1.02(0.87-1.20)		HF
205	DBP					HF
206	≤median	134879	989	0.95(0.80-1.13)	>0.05	HF
207	>median	117589	1041	0.97(0.81-1.15)		HF
208	Smoke status					HF
209	No	155069	969	1.20(1.00-1.44)	0.006	HF
210	Yes	115276	1231	0.86(0.73-1.00)		HF
211	Sex					HS
212	Male	156041	688	1.16(0.93-1.43)	>0.05	HS
213	Female	116005	634	0.97(0.78-1.22)		HS
214	Age					HS
215	≤median	143960	526	1.04(0.82-1.33)	>0.05	HS
216	>median	128086	796	1.10(0.90-1.34)		HS
217	BMI					HS

218	≤median	135656	664	1.10(0.88-1.38)	>0.05	HS
219	>median	135658	649	1.01(0.82-1.25)		HS
220	Fasting time					HS
221	≤median	152808	687	0.91(0.73-1.13)	0.022	HS
222	>median	119229	635	1.25(1.00-1.56)		HS
223	HDL-C					HS
224	≤median	136113	686	1.09(0.88-1.36)	>0.05	HS
225	>median	135879	636	1.01(0.81-1.27)		HS
226	LDL-C					HS
227	≤median	135845	694	1.11(0.90-1.37)	>0.05	HS
228	>median	135817	626	1.04(0.83-1.30)		HS
229	HbA1c					HS
230	≤median	131109	568	1.02(0.80-1.30)	>0.05	HS
231	>median	128671	691	1.10(0.89-1.36)		HS
232	SBP					HS
233	≤median	128884	513	1.15(0.90-1.46)	>0.05	HS
234	>median	124400	697	1.05(0.84-1.30)		HS
235	DBP					HS
236	≤median	135278	590	1.18(0.94-1.48)	>0.05	HS
237	>median	118010	620	1.01(0.81-1.27)		HS
238	Smoke status					HS
239	No	155424	614	1.05(0.84-1.31)	>0.05	HS
240	Yes	115802	705	1.08(0.87-1.34)		HS
241	Sex					IS
242	Male	156052	677	1.13(0.91-1.39)	>0.05	IS
243	Female	115620	1019	1.12(0.94-1.34)		IS
244	Age					IS
245	≤median	144028	458	1.32(1.02-1.72)	>0.05	IS
246	>median	127644	1238	1.07(0.92-1.26)		IS
247	BMI					IS
248	≤median	135583	737	1.00(0.82-1.23)	>0.05	IS
249	>median	135359	948	1.23(1.02-1.48)		IS
250	Fasting time					IS
251	≤median	152604	891	1.00(0.82-1.20)	0.040	IS
252	>median	119059	805	1.26(1.04-1.54)		IS
253	HDL-C					IS
254	≤median	135752	1047	0.98(0.82-1.17)	>0.05	IS
255	>median	135866	649	1.17(0.94-1.46)		IS
256	LDL-C					IS
257	≤median	135596	943	1.29(1.08-1.56)	0.013	IS
258	>median	135692	751	0.96(0.79-1.18)		IS
259	HbA1c					IS
260	≤median	131026	651	1.19(0.96-1.48)	>0.05	IS
261	>median	128390	972	1.11(0.93-1.33)		IS

SBP					IS
≤median	128845	552	1.22(0.96-1.55)	>0.05	IS
>median	124114	983	1.15(0.96-1.37)		IS
DBP					IS
≤median	135176	692	1.27(1.02-1.56)	>0.05	IS
>median	117787	843	1.10(0.90-1.34)		IS
Smoke status					IS
No	155278	760	1.26(1.03-1.55)	>0.05	IS
Yes	115581	926	1.01(0.84-1.22)		IS

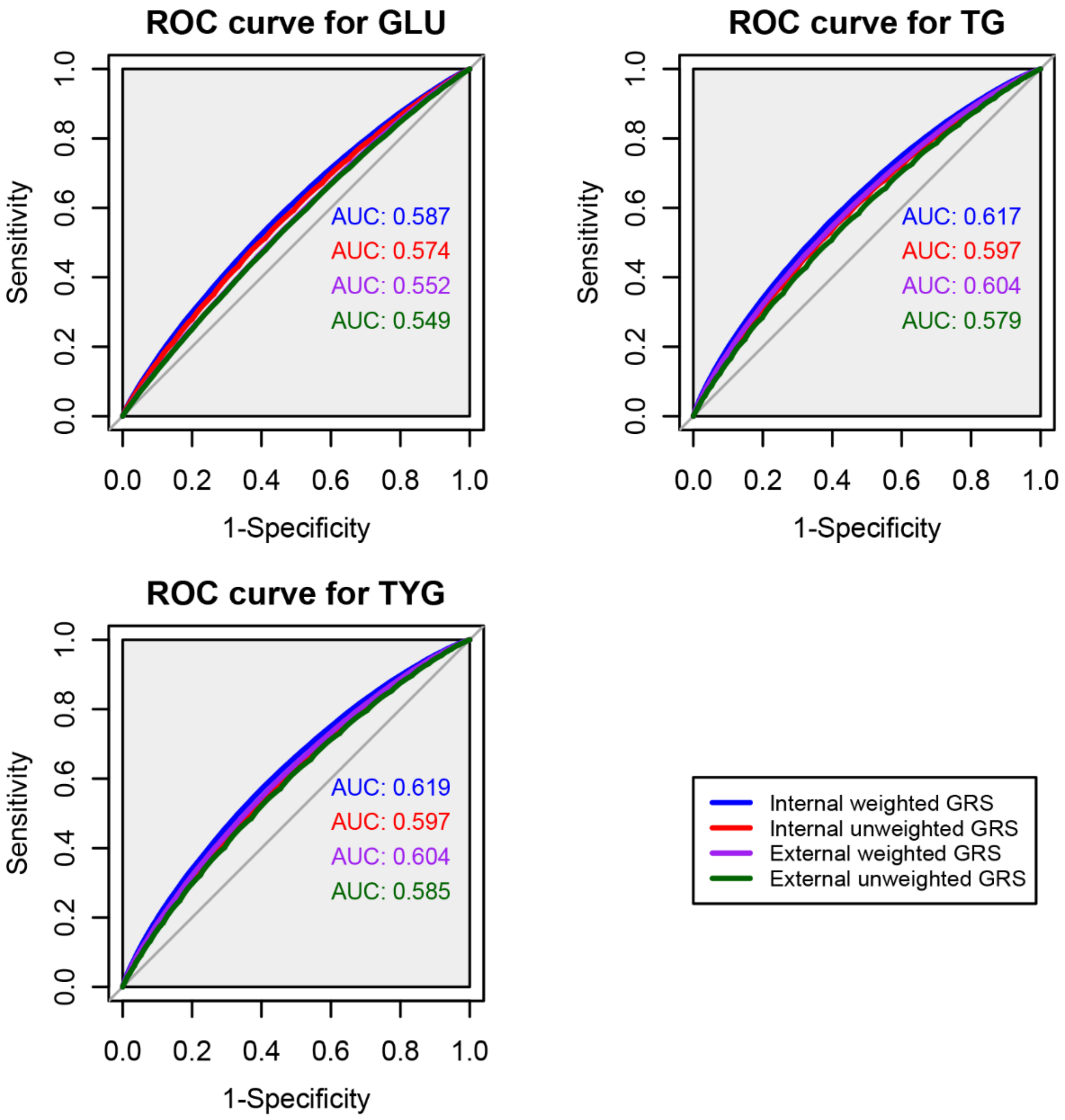

Figure S1. ROC curve for internal/external and weighted/unweighted GRS.

GLU by TG subgroup	No.Control	No.Case	ΔGLU	Odds Ratio	P value
**AP**					**Q test:>0.05**
Q1	66892	1450	0.62(1-SD)	1.61(1.05-2.47)	0.028
Q2	66902	1440	0.62(1-SD)	1.06(0.67-1.68)	0.797
Q3	66827	1515	0.62(1-SD)	1.41(0.91-2.17)	0.120
Q4	66831	1511	0.62(1-SD)	1.32(0.86-2.03)	0.201
**Overall**	**267452**	**5916**	**0.62(1-SD)**	**1.34(1.08-1.66)**	**0.009**
**AMI**					**Q test:>0.05**
Q1	67407	935	0.62(1-SD)	1.21(0.72-2.06)	0.469
Q2	67382	960	0.62(1-SD)	1.53(0.88-2.67)	0.132
Q3	67286	1056	0.62(1-SD)	1.52(0.91-2.55)	0.110
Q4	67256	1086	0.62(1-SD)	2.00(1.21-3.30)	0.007
**Overall**	**269331**	**4037**	**0.62(1-SD)**	**1.54(1.19-2.00)**	**0.001**
**CIHD**					**Q test:>0.05**
Q1	66547	1795	0.62(1-SD)	1.63(1.11-2.40)	0.012
Q2	66464	1878	0.62(1-SD)	1.12(0.75-1.68)	0.572
Q3	66447	1895	0.62(1-SD)	1.38(0.93-2.03)	0.108
Q4	66384	1958	0.62(1-SD)	1.44(0.98-2.10)	0.062
**Overall**	**265842**	**7526**	**0.62(1-SD)**	**1.38(1.13-1.67)**	**0.001**
**HF**					**Q test:>0.05**
Q1	67799	543	0.62(1-SD)	0.95(0.48-1.88)	0.872
Q2	67808	534	0.62(1-SD)	0.92(0.44-1.92)	0.820
Q3	67749	593	0.62(1-SD)	0.94(0.48-1.86)	0.864
Q4	67801	541	0.62(1-SD)	1.78(0.88-3.61)	0.108
**Overall**	**271157**	**2211**	**0.62(1-SD)**	**1.09(0.77-1.55)**	**0.615**
**HS**					**Q test:>0.05**
Q1	68041	301	0.62(1-SD)	0.58(0.23-1.43)	0.234
Q2	67989	353	0.62(1-SD)	0.75(0.30-1.84)	0.529
Q3	67989	353	0.62(1-SD)	1.44(0.60-3.45)	0.416
Q4	68027	315	0.62(1-SD)	0.67(0.27-1.68)	0.390
**Overall**	**272046**	**1322**	**0.62(1-SD)**	**0.81(0.52-1.28)**	**0.370**
**IS**					**Q test:>0.05**
Q1	67921	421	0.62(1-SD)	1.10(0.51-2.38)	0.814
Q2	67942	400	0.62(1-SD)	0.88(0.38-2.05)	0.762
Q3	67915	427	0.62(1-SD)	1.13(0.51-2.51)	0.771
Q4	67894	448	0.62(1-SD)	0.74(0.34-1.60)	0.447
**Overall**	**271672**	**1696**	**0.62(1-SD)**	**0.94(0.63-1.40)**	**0.771**

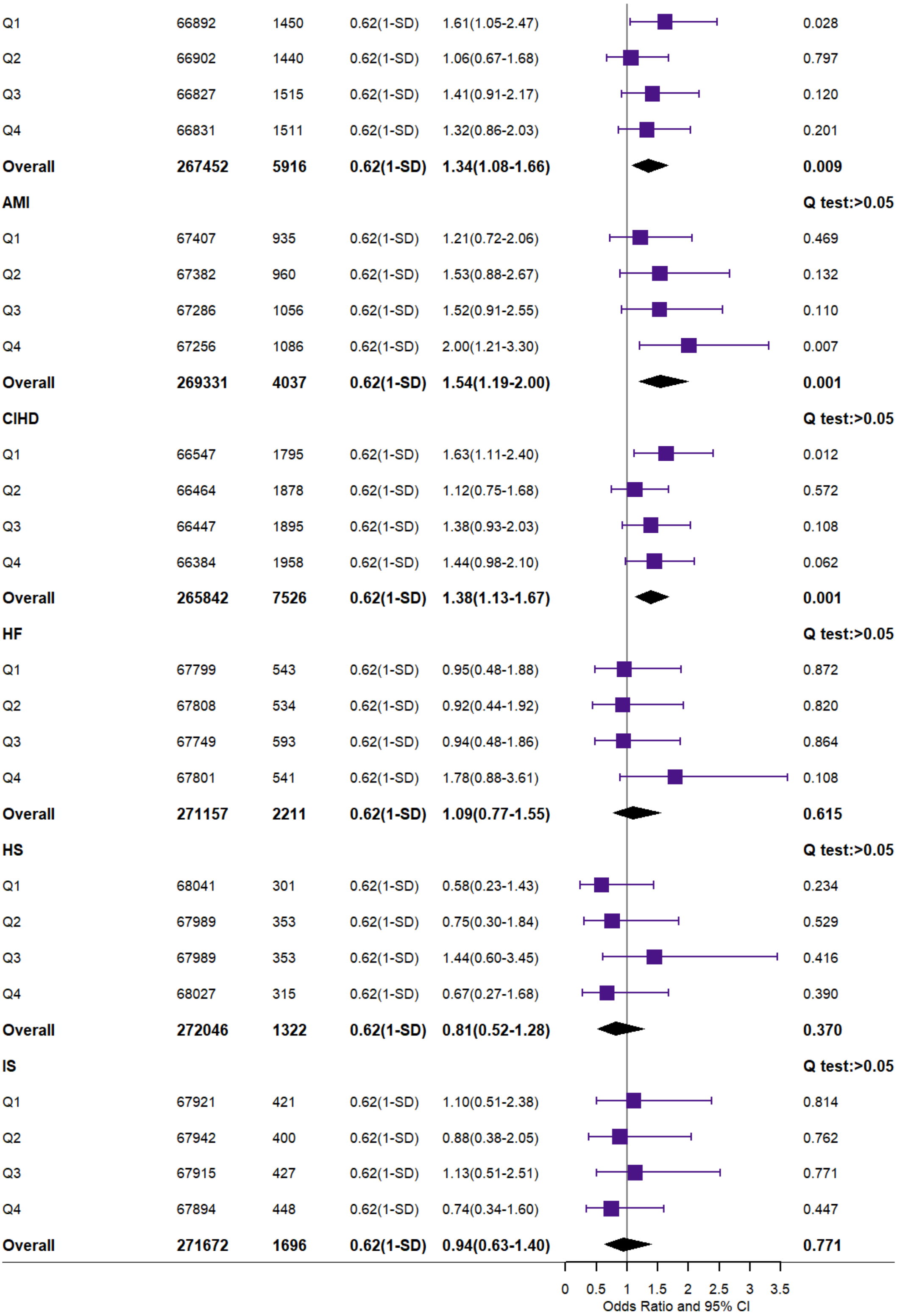

Figure S2. Assessment of independent higher GLU in scaled difference with the risk of minor CVD by weighted GRS in dataset A.

TG by GLU subgroup	No.Control	No.Case	Δlog(TG)	Odds Ratio	P value
**AP**					**Q test:>0.05**
Q1	66886	1456	0.50(1-SD)	1.18(0.92-1.51)	0.204
Q2	66911	1431	0.50(1-SD)	1.62(1.25-2.10)	<0.001
Q3	66824	1518	0.50(1-SD)	1.28(1.00-1.64)	0.051
Q4	66831	1511	0.50(1-SD)	1.56(1.22-2.00)	<0.001
**Overall**	**267452**	**5916**	**0.50(1-SD)**	**1.39(1.23-1.58)**	**<0.001**
**AMI**					**Q test:>0.05**
Q1	67392	950	0.50(1-SD)	1.33(0.98-1.81)	0.065
Q2	67359	983	0.50(1-SD)	1.73(1.26-2.36)	<0.001
Q3	67313	1029	0.50(1-SD)	1.81(1.34-2.45)	<0.001
Q4	67267	1075	0.50(1-SD)	1.70(1.27-2.28)	<0.001
**Overall**	**269331**	**4037**	**0.50(1-SD)**	**1.64(1.41-1.91)**	**<0.001**
**CIHD**					**Q test:>0.05**
Q1	66569	1773	0.50(1-SD)	1.27(1.01-1.60)	0.038
Q2	66461	1881	0.50(1-SD)	1.40(1.12-1.76)	0.004
Q3	66484	1858	0.50(1-SD)	1.41(1.13-1.77)	0.003
Q4	66328	2014	0.50(1-SD)	1.32(1.07-1.64)	0.011
**Overall**	**265842**	**7526**	**0.50(1-SD)**	**1.35(1.21-1.51)**	**<0.001**
**HF**					**Q test:>0.05**
Q1	67786	556	0.50(1-SD)	1.37(0.92-2.05)	0.120
Q2	67794	548	0.50(1-SD)	1.10(0.73-1.66)	0.652
Q3	67802	540	0.50(1-SD)	1.63(1.08-2.46)	0.019
Q4	67775	567	0.50(1-SD)	1.37(0.92-2.04)	0.120
**Overall**	**271157**	**2211**	**0.50(1-SD)**	**1.36(1.11-1.66)**	**0.003**
**HS**					**Q test:>0.05**
Q1	68021	321	0.50(1-SD)	0.97(0.58-1.62)	0.896
Q2	67986	356	0.50(1-SD)	1.00(0.60-1.66)	0.997
Q3	68011	331	0.50(1-SD)	1.60(0.95-2.69)	0.078
Q4	68028	314	0.50(1-SD)	0.98(0.58-1.66)	0.934
**Overall**	**272046**	**1322**	**0.50(1-SD)**	**1.11(0.86-1.44)**	**0.436**
**IS**					**Q test:>0.05**
Q1	67901	441	0.50(1-SD)	1.29(0.83-2.02)	0.259
Q2	67923	419	0.50(1-SD)	1.43(0.90-2.29)	0.132
Q3	67926	416	0.50(1-SD)	1.24(0.78-1.97)	0.360
Q4	67922	420	0.50(1-SD)	1.33(0.84-2.10)	0.226
**Overall**	**271672**	**1696**	**0.50(1-SD)**	**1.32(1.05-1.67)**	**0.017**

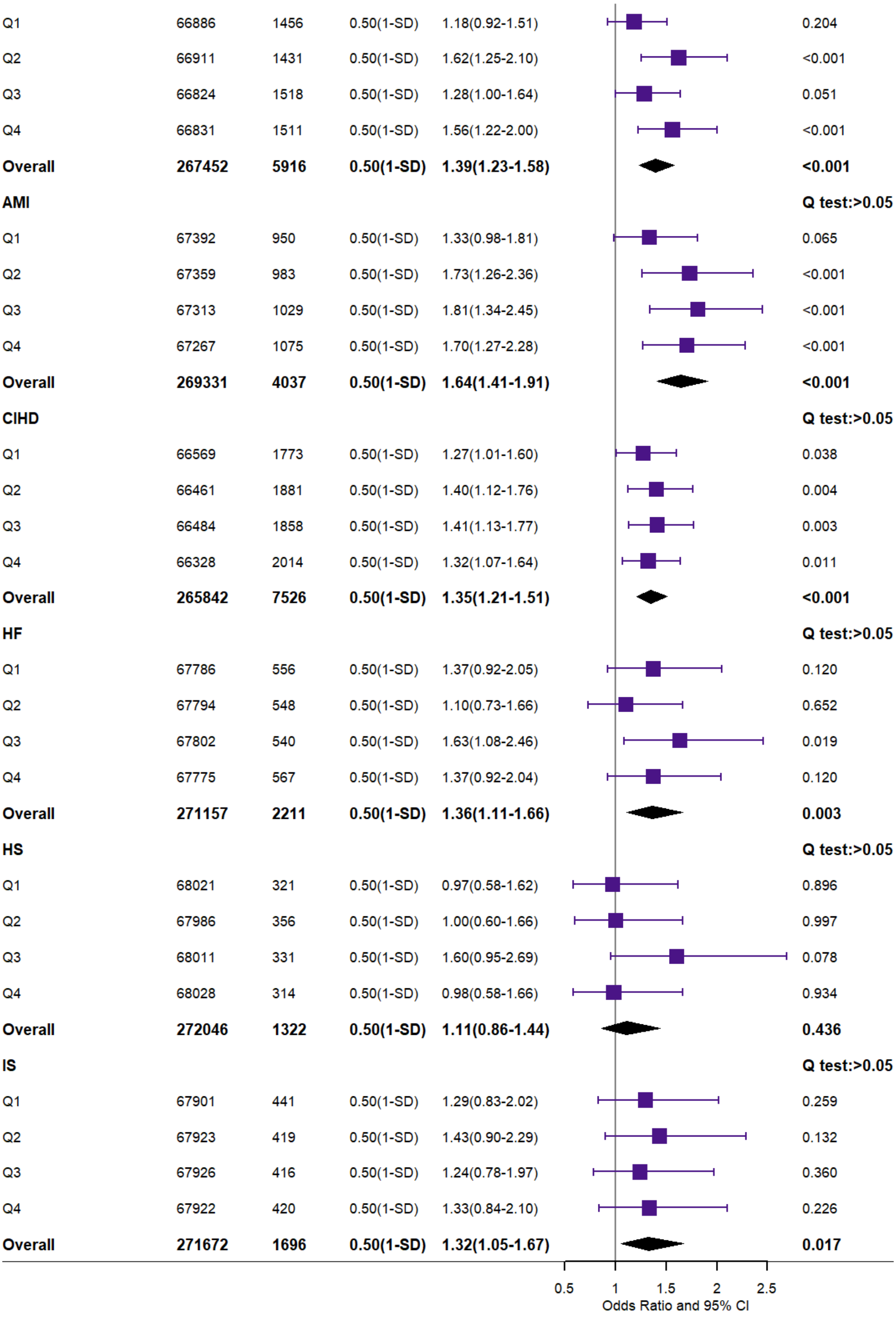

Figure S3. Assessment of independent higher TG in scaled difference with the risk of minor CVD by weighted GRS in dataset A.

GLU by TG subgroup	No.control	No.case	ΔGLU	Odds Ratio	P value
**Overall CVD**					**Q test:>0.05**
Q1	64267	4075	0.155	1.04(0.97-1.11)	0.236
Q2	64300	4042	0.145	1.02(0.96-1.09)	0.553
Q3	64096	4246	0.149	1.06(0.99-1.13)	0.091
Q4	64158	4184	0.151	1.05(0.99-1.12)	0.116
**Overall**	**256821**	**16547**	**0.150**	**1.04(1.01-1.08)**	**0.013**
**IHD**					**Q test:>0.05**
Q1	65672	2670	0.155	1.11(1.03-1.20)	0.009
Q2	65617	2725	0.145	1.01(0.94-1.10)	0.745
Q3	65518	2824	0.149	1.09(1.01-1.18)	0.034
Q4	65523	2819	0.151	1.10(1.02-1.19)	0.014
**Overall**	**262330**	**11038**	**0.150**	**1.08(1.04-1.12)**	**<0.001**
**CED**					**Q test:>0.05**
Q1	67025	1317	0.155	0.96(0.86-1.07)	0.419
Q2	67086	1256	0.145	0.99(0.89-1.11)	0.886
Q3	67004	1338	0.149	1.01(0.90-1.13)	0.887
Q4	67039	1303	0.151	0.96(0.86-1.07)	0.462
**Overall**	**268154**	**5214**	**0.150**	**0.98(0.93-1.03)**	**0.435**
**TG by GLU subgroup**	**No.Control**	**No.Case**	**Δlog(TG)**	**Odds Ratio**	**P value**
**CVD**					**Q test:>0.05**
Q1	64321	4021	0.213	1.09(1.02-1.16)	0.010
Q2	64182	4160	0.207	1.13(1.06-1.20)	<0.001
Q3	64166	4176	0.210	1.14(1.07-1.21)	<0.001
Q4	64152	4190	0.211	1.14(1.07-1.22)	<0.001
**Overall**	**256821**	**16547**	**0.210**	**1.12(1.09-1.16)**	**<0.001**
**IHD**					**Q test:>0.05**
Q1	65699	2643	0.213	1.09(1.01-1.18)	0.036
Q2	65612	2730	0.207	1.14(1.06-1.24)	<0.001
Q3	65544	2798	0.210	1.13(1.05-1.23)	0.002
Q4	65475	2867	0.211	1.17(1.09-1.27)	<0.001
**Overall**	**262330**	**11038**	**0.210**	**1.14(1.09-1.18)**	**<0.001**
**CED**					**Q test:>0.05**
Q1	67035	1307	0.213	1.07(0.95-1.19)	0.258
Q2	66999	1343	0.207	1.10(0.99-1.23)	0.074
Q3	67021	1321	0.210	1.11(0.99-1.24)	0.065
Q4	67099	1243	0.211	1.09(0.97-1.22)	0.137
**Overall**	**268154**	**5214**	**0.210**	**1.09(1.03-1.16)**	**0.002**

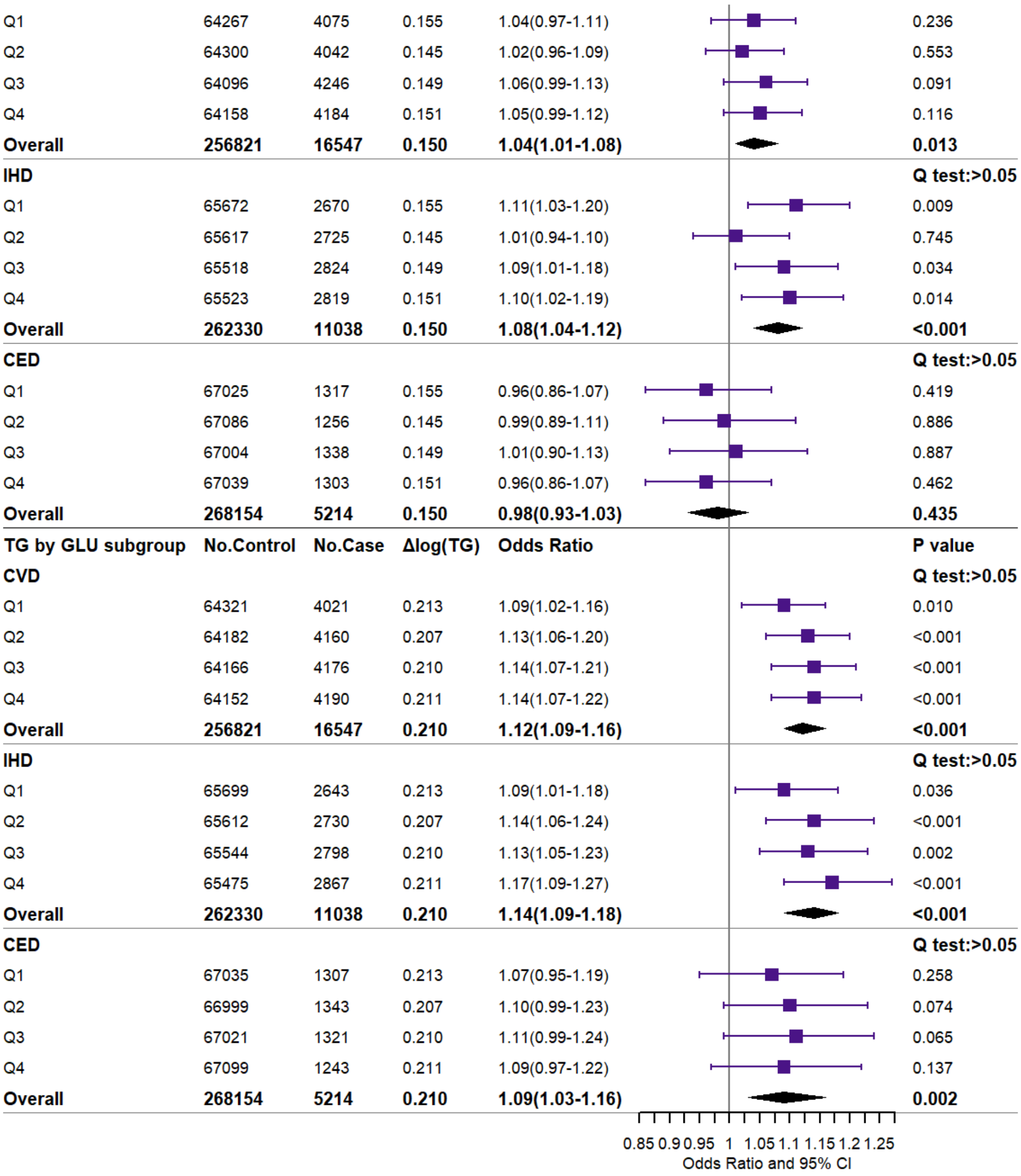

Figure S4. Assessment of independent higher GLU and TG in crude difference with the risk of major CVD by weighted GRS in dataset A.

GLU by TG subgroup	No.control	No.case	ΔGLU	Odds Ratio	P value
**AP**					**Q test:>0.05**
Q1	66892	1450	0.155	1.13(1.01-1.25)	0.028
Q2	66902	1440	0.145	1.01(0.91-1.13)	0.797
Q3	66827	1515	0.149	1.09(0.98-1.20)	0.120
Q4	66831	1511	0.151	1.07(0.96-1.19)	0.201
**Overall**	**267452**	**5916**	**0.150**	**1.07(1.02-1.13)**	**0.009**
**AMI**					**Q test:>0.05**
Q1	67407	935	0.155	1.05(0.92-1.20)	0.469
Q2	67382	960	0.145	1.10(0.97-1.26)	0.132
Q3	67286	1056	0.149	1.11(0.98-1.25)	0.110
Q4	67256	1086	0.151	1.18(1.05-1.34)	0.007
**Overall**	**269331**	**4037**	**0.150**	**1.11(1.04-1.18)**	**0.001**
**CIHD**					**Q test:>0.05**
Q1	66547	1795	0.155	1.13(1.03-1.24)	0.012
Q2	66464	1878	0.145	1.03(0.94-1.13)	0.572
Q3	66447	1895	0.149	1.08(0.98-1.19)	0.108
Q4	66384	1958	0.151	1.09(1.00-1.20)	0.062
**Overall**	**265842**	**7526**	**0.150**	**1.08(1.03-1.13)**	**0.001**
**HF**					**Q test:>0.05**
Q1	67799	543	0.155	0.99(0.83-1.17)	0.872
Q2	67808	534	0.145	0.98(0.82-1.17)	0.820
Q3	67749	593	0.149	0.99(0.84-1.16)	0.864
Q4	67801	541	0.151	1.15(0.97-1.37)	0.108
**Overall**	**271157**	**2211**	**0.150**	**1.02(0.94-1.11)**	**0.615**
**HS**					**Q test:>0.05**
Q1	68041	301	0.155	0.87(0.69-1.09)	0.234
Q2	67989	353	0.145	0.93(0.76-1.15)	0.529
Q3	67989	353	0.149	1.09(0.88-1.35)	0.416
Q4	68027	315	0.151	0.91(0.72-1.13)	0.390
**Overall**	**272046**	**1322**	**0.150**	**0.95(0.85-1.06)**	**0.370**
**IS**					**Q test:>0.05**
Q1	67921	421	0.155	1.02(0.84-1.24)	0.814
Q2	67942	400	0.145	0.97(0.80-1.18)	0.762
Q3	67915	427	0.149	1.03(0.85-1.25)	0.771
Q4	67894	448	0.151	0.93(0.77-1.12)	0.447
**Overall**	**271672**	**1696**	**0.150**	**0.99(0.90-1.09)**	**0.771**

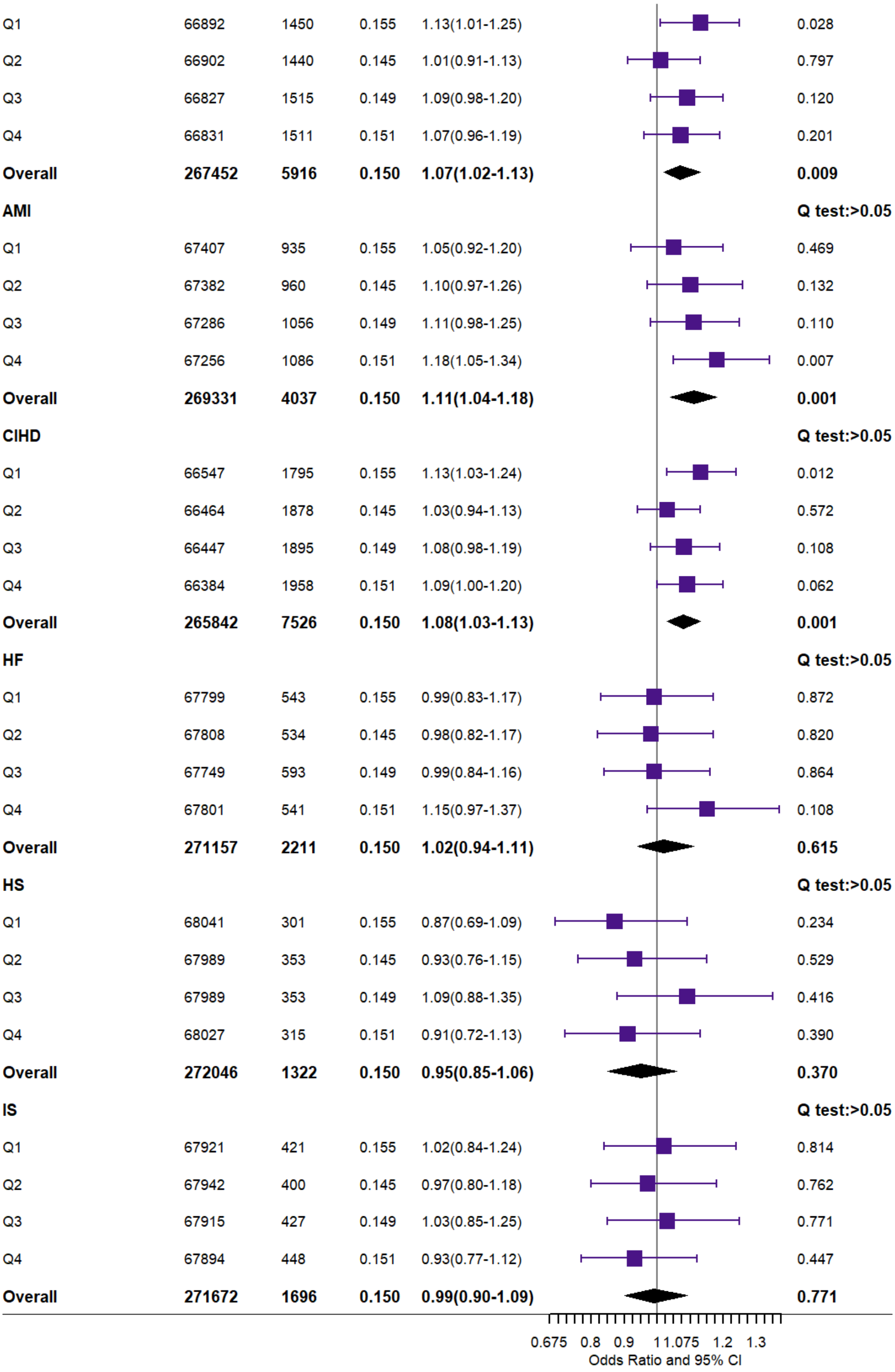

Figure S5. Assessment of independent higher GLU in crude difference with the risk of minor CVD by weighted GRS in dataset A.

TG by GLU subgroup	No.Control	No.Case	Δlog(TG)	Odds Ratio	P value
**AP**					**Q test:>0.05**
Q1	66886	1456	0.213	1.07(0.96-1.19)	0.204
Q2	66911	1431	0.207	1.22(1.10-1.36)	<0.001
Q3	66824	1518	0.210	1.11(1.00-1.23)	0.051
Q4	66831	1511	0.211	1.21(1.09-1.34)	<0.001
**Overall**	**267452**	**5916**	**0.210**	**1.15(1.09-1.21)**	**<0.001**
**AMI**					**Q test:>0.05**
Q1	67392	950	0.213	1.13(0.99-1.29)	0.065
Q2	67359	983	0.207	1.25(1.10-1.43)	<0.001
Q3	67313	1029	0.210	1.28(1.13-1.46)	<0.001
Q4	67267	1075	0.211	1.25(1.11-1.42)	<0.001
**Overall**	**269331**	**4037**	**0.210**	**1.23(1.16-1.31)**	**<0.001**
**CIHD**					**Q test:>0.05**
Q1	66569	1773	0.213	1.11(1.01-1.22)	0.038
Q2	66461	1881	0.207	1.15(1.05-1.26)	0.004
Q3	66484	1858	0.210	1.16(1.05-1.27)	0.003
Q4	66328	2014	0.211	1.13(1.03-1.23)	0.011
**Overall**	**265842**	**7526**	**0.210**	**1.13(1.08-1.19)**	**<0.001**
**HF**					**Q test:>0.05**
Q1	67786	556	0.213	1.14(0.97-1.36)	0.120
Q2	67794	548	0.207	1.04(0.88-1.23)	0.652
Q3	67802	540	0.210	1.23(1.03-1.46)	0.019
Q4	67775	567	0.211	1.14(0.97-1.35)	0.120
**Overall**	**271157**	**2211**	**0.210**	**1.14(1.04-1.24)**	**0.003**
**HS**					**Q test:>0.05**
Q1	68021	321	0.213	0.99(0.79-1.23)	0.896
Q2	67986	356	0.207	1.00(0.81-1.23)	0.997
Q3	68011	331	0.210	1.22(0.98-1.52)	0.078
Q4	68028	314	0.211	0.99(0.79-1.24)	0.934
**Overall**	**272046**	**1322**	**0.210**	**1.04(0.94-1.16)**	**0.436**
**IS**					**Q test:>0.05**
Q1	67901	441	0.213	1.12(0.92-1.35)	0.259
Q2	67923	419	0.207	1.16(0.96-1.41)	0.132
Q3	67926	416	0.210	1.10(0.90-1.33)	0.360
Q4	67922	420	0.211	1.13(0.93-1.37)	0.226
**Overall**	**271672**	**1696**	**0.210**	**1.13(1.02-1.24)**	**0.017**

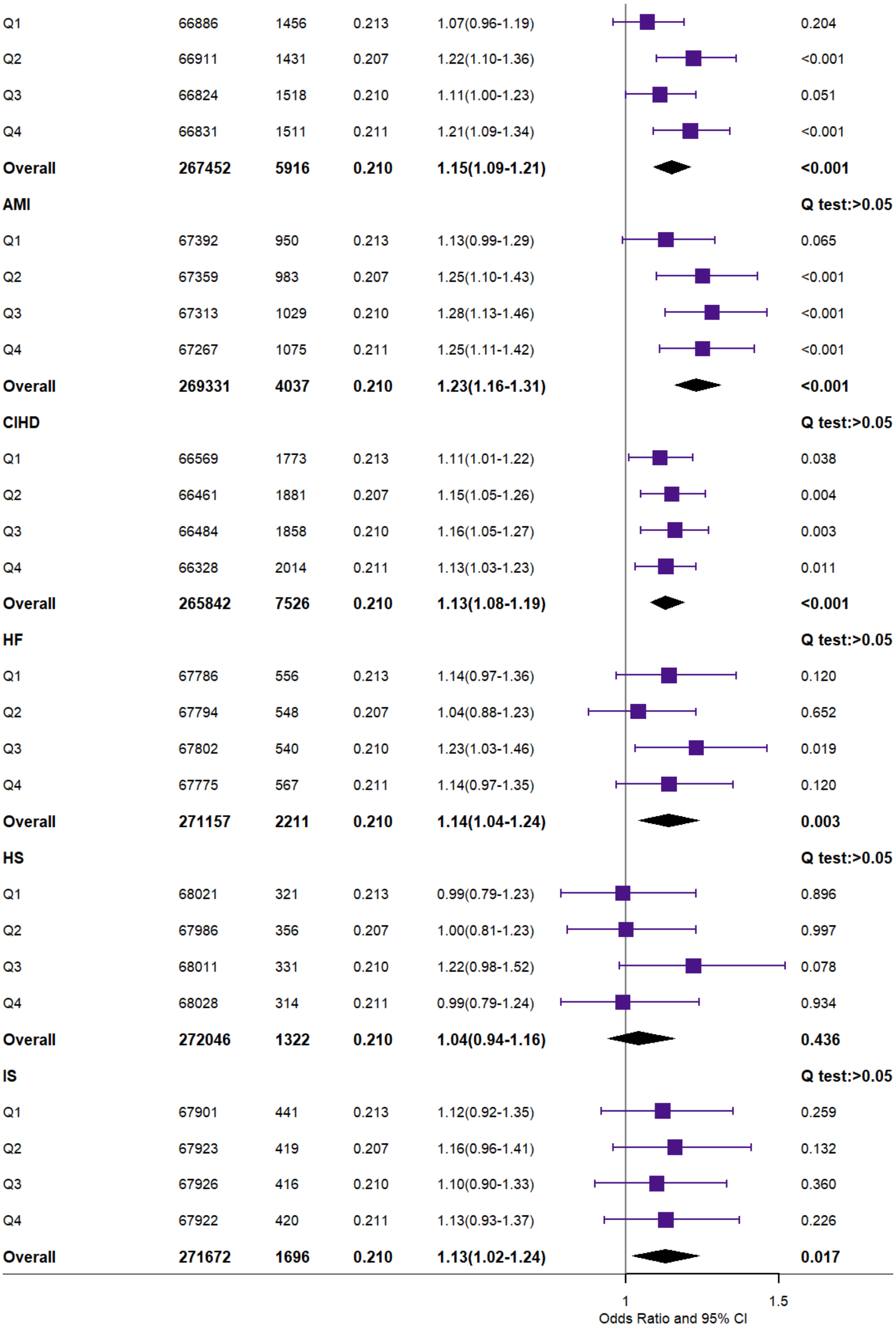

Figure S6. Assessment of independent higher TG in crude difference with the risk of minor CVD by weighted GRS in dataset A.

GLU by TG subgroup	No.Control	No.Case	ΔGLU	Odds Ratio	P value
**Ovrall CVD**					**Q test:>0.05**
Q1	64296	4046	0.62(1-SD)	0.89(0.66-1.19)	0.434
Q2	64249	4093	0.62(1-SD)	1.16(0.85-1.58)	0.359
Q3	64195	4147	0.62(1-SD)	1.15(0.85-1.57)	0.364
Q4	64081	4261	0.62(1-SD)	1.25(0.91-1.71)	0.171
**Overall**	**256821**	**16547**	**0.62(1-SD)**	**1.09(0.94-1.27)**	**0.266**
**IHD**					**Q test:>0.05**
Q1	65638	2704	0.62(1-SD)	0.96(0.67-1.37)	0.824
Q2	65638	2704	0.62(1-SD)	1.26(0.86-1.84)	0.239
Q3	65574	2768	0.62(1-SD)	1.34(0.93-1.94)	0.119
Q4	65480	2862	0.62(1-SD)	1.61(1.10-2.35)	0.013
**Overall**	**262330**	**11038**	**0.62(1-SD)**	**1.26(1.05-1.51)**	**0.015**
**CED**					**Q test:>0.05**
Q1	67053	1289	0.62(1-SD)	0.84(0.51-1.38)	0.484
Q2	67056	1286	0.62(1-SD)	1.08(0.63-1.85)	0.777
Q3	67029	1313	0.62(1-SD)	0.73(0.43-1.23)	0.233
Q4	67016	1326	0.62(1-SD)	0.65(0.38-1.12)	0.124
**Overall**	**268154**	**5214**	**0.62(1-SD)**	**0.80(0.62-1.04)**	**0.102**
**TG by GLU subgroup**	**No.Control**	**No.Case**	**Δlog(TG)**	**Odds Ratio**	**P value**
**CVD**					**Q test:>0.05**
Q1	64186	4156	0.50(1-SD)	1.30(1.08-1.57)	0.005
Q2	64220	4122	0.50(1-SD)	1.23(1.02-1.50)	0.033
Q3	64247	4095	0.50(1-SD)	1.43(1.19-1.73)	<0.001
Q4	64168	4174	0.50(1-SD)	1.41(1.17-1.71)	<0.001
**Overall**	**256821**	**16547**	**0.50(1-SD)**	**1.34(1.22-1.48)**	**<0.001**
**IHD**					**Q test:>0.05**
Q1	65587	2755	0.50(1-SD)	1.37(1.09-1.71)	0.006
Q2	65645	2697	0.50(1-SD)	1.16(0.92-1.47)	0.218
Q3	65568	2774	0.50(1-SD)	1.47(1.17-1.84)	<0.001
Q4	65530	2812	0.50(1-SD)	1.57(1.25-1.98)	<0.001
**Overall**	**262330**	**11038**	**0.50(1-SD)**	**1.38(1.23-1.55)**	**<0.001**
**CED**					**Q test:>0.05**
Q1	67013	1329	0.50(1-SD)	1.36(0.99-1.86)	0.057
Q2	66988	1354	0.50(1-SD)	1.43(1.03-1.97)	0.031
Q3	67091	1251	0.50(1-SD)	1.14(0.82-1.58)	0.444
Q4	67062	1280	0.50(1-SD)	1.18(0.85-1.65)	0.325
**Overall**	**268154**	**5214**	**0.50(1-SD)**	**1.27(1.08-1.50)**	**0.003**

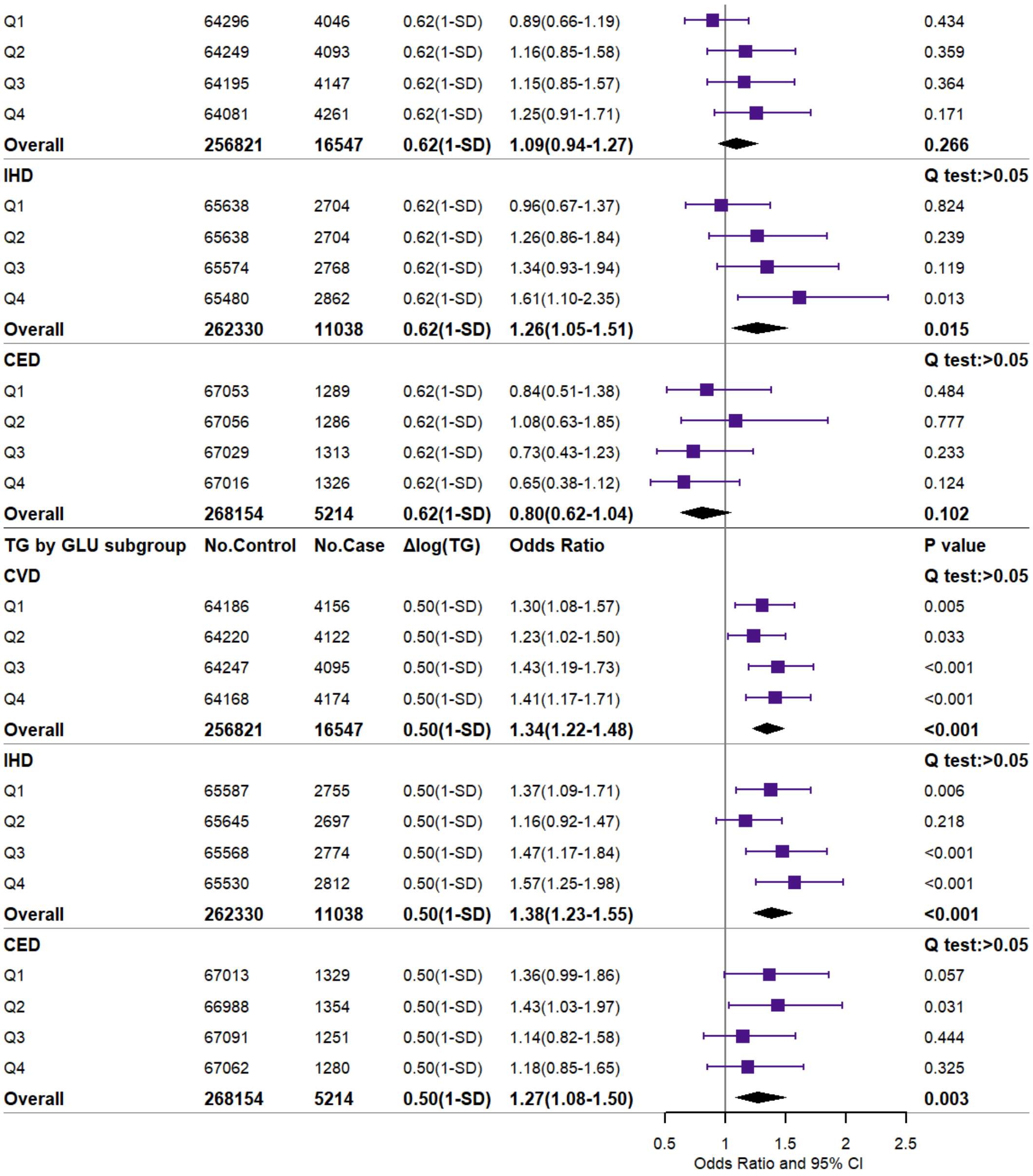

Figure S7. Assessment of independent higher GLU and TG in scaled difference with the risk of major CVD by unweighted GRS in dataset A.

GLU by TG subgroup	No.Control	No.Case	ΔGLU	Odds Ratio	P value
**AP**					**Q test:>0.05**
Q1	66871	1471	0.62(1-SD)	0.87(0.54-1.40)	0.570
Q2	66903	1439	0.62(1-SD)	1.25(0.75-2.08)	0.399
Q3	66883	1459	0.62(1-SD)	1.29(0.78-2.12)	0.323
Q4	66795	1547	0.62(1-SD)	1.27(0.76-2.10)	0.361
**Overall**	**267452**	**5916**	**0.62(1-SD)**	**1.14(0.89-1.47)**	**0.295**
**AMI**					**Q test:>0.05**
Q1	67381	961	0.62(1-SD)	1.07(0.60-1.91)	0.828
Q2	67333	1009	0.62(1-SD)	1.59(0.87-2.93)	0.132
Q3	67341	1001	0.62(1-SD)	1.27(0.69-2.31)	0.444
Q4	67276	1066	0.62(1-SD)	1.99(1.09-3.66)	0.025
**Overall**	**269331**	**4037**	**0.62(1-SD)**	**1.43(1.06-1.93)**	**0.020**
**CIHD**					**Q test:>0.05**
Q1	66512	1830	0.62(1-SD)	1.07(0.70-1.64)	0.768
Q2	66514	1828	0.62(1-SD)	1.23(0.78-1.94)	0.379
Q3	66462	1880	0.62(1-SD)	1.34(0.86-2.10)	0.195
Q4	66354	1988	0.62(1-SD)	1.78(1.14-2.80)	0.012
**Overall**	**265842**	**7526**	**0.62(1-SD)**	**1.32(1.06-1.65)**	**0.015**
**HF**					**Q test:>0.05**
Q1	67787	555	0.62(1-SD)	1.13(0.53-2.42)	0.753
Q2	67781	561	0.62(1-SD)	0.90(0.40-2.03)	0.809
Q3	67795	547	0.62(1-SD)	1.97(0.88-4.45)	0.101
Q4	67794	548	0.62(1-SD)	1.71(0.74-3.94)	0.211
**Overall**	**271157**	**2211**	**0.62(1-SD)**	**1.35(0.90-2.01)**	**0.148**
**HS**					**Q test:>0.05**
Q1	68042	300	0.62(1-SD)	0.58(0.21-1.61)	0.292
Q2	67991	351	0.62(1-SD)	0.97(0.35-2.66)	0.949
Q3	67999	343	0.62(1-SD)	0.62(0.22-1.70)	0.348
Q4	68014	328	0.62(1-SD)	0.71(0.24-2.07)	0.528
**Overall**	**272046**	**1322**	**0.62(1-SD)**	**0.70(0.42-1.17)**	**0.177**
**IS**					**Q test:>0.05**
Q1	67931	411	0.62(1-SD)	1.60(0.66-3.88)	0.296
Q2	67920	422	0.62(1-SD)	1.44(0.57-3.66)	0.444
Q3	67929	413	0.62(1-SD)	0.80(0.32-2.03)	0.645
Q4	67892	450	0.62(1-SD)	0.62(0.25-1.56)	0.310
**Overall**	**271672**	**1696**	**0.62(1-SD)**	**1.02(0.65-1.61)**	**0.923**

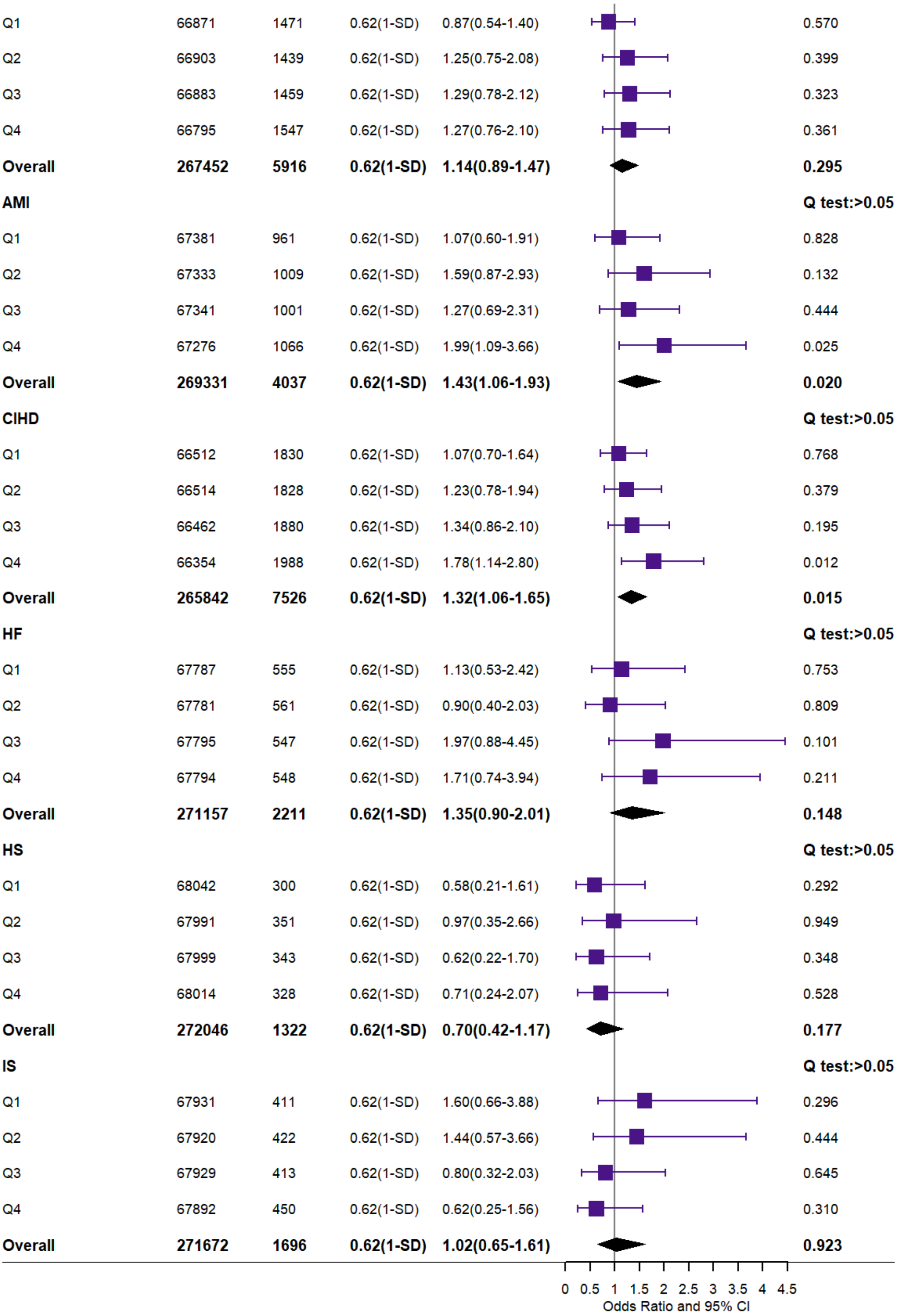

Figure S8. Assessment of independent higher GLU in scaled difference with the risk of minor CVD by unweighted GRS in dataset A.

TG by GLU subgroup	No.Control	No.Case	Δlog(TG)	Odds Ratio	P value
**AP**					**Q test:>0.05**
Q1	66859	1483	0.50(1-SD)	1.54(1.14-2.08)	0.005
Q2	66871	1471	0.50(1-SD)	1.11(0.81-1.52)	0.522
Q3	66861	1481	0.50(1-SD)	1.51(1.12-2.05)	0.007
Q4	66861	1481	0.50(1-SD)	1.49(1.09-2.04)	0.012
**Overall**	**267452**	**5916**	**0.50(1-SD)**	**1.40(1.20-1.64)**	**<0.001**
**AMI**					**Q test:>0.05**
Q1	67347	995	0.50(1-SD)	1.28(0.89-1.84)	0.180
Q2	67367	975	0.50(1-SD)	1.40(0.96-2.06)	0.082
Q3	67318	1024	0.50(1-SD)	1.35(0.94-1.93)	0.105
Q4	67299	1043	0.50(1-SD)	1.68(1.16-2.43)	0.006
**Overall**	**269331**	**4037**	**0.50(1-SD)**	**1.42(1.18-1.70)**	**<0.001**
**CIHD**					**Q test:>0.05**
Q1	66488	1854	0.50(1-SD)	1.34(1.02-1.75)	0.033
Q2	66495	1847	0.50(1-SD)	1.28(0.97-1.69)	0.085
Q3	66449	1893	0.50(1-SD)	1.46(1.11-1.90)	0.006
Q4	66410	1932	0.50(1-SD)	1.70(1.29-2.24)	<0.001
**Overall**	**265842**	**7526**	**0.50(1-SD)**	**1.43(1.25-1.64)**	**<0.001**
**HF**					**Q test:>0.05**
Q1	67800	542	0.50(1-SD)	0.95(0.58-1.54)	0.820
Q2	67797	545	0.50(1-SD)	0.91(0.55-1.52)	0.721
Q3	67795	547	0.50(1-SD)	1.20(0.73-1.95)	0.477
Q4	67765	577	0.50(1-SD)	1.55(0.94-2.54)	0.083
**Overall**	**271157**	**2211**	**0.50(1-SD)**	**1.12(0.88-1.44)**	**0.355**
**HS**					**Q test:>0.05**
Q1	68005	337	0.50(1-SD)	1.03(0.56-1.90)	0.923
Q2	67992	350	0.50(1-SD)	1.37(0.73-2.57)	0.320
Q3	68033	309	0.50(1-SD)	1.19(0.62-2.26)	0.604
Q4	68016	326	0.50(1-SD)	1.05(0.54-2.02)	0.890
**Overall**	**272046**	**1322**	**0.50(1-SD)**	**1.15(0.84-1.59)**	**0.372**
**IS**					**Q test:>0.05**
Q1	67917	425	0.50(1-SD)	1.91(1.10-3.32)	0.022
Q2	67912	430	0.50(1-SD)	1.48(0.84-2.61)	0.176
Q3	67945	397	0.50(1-SD)	0.81(0.46-1.43)	0.466
Q4	67898	444	0.50(1-SD)	1.31(0.75-2.30)	0.349
**Overall**	**271672**	**1696**	**0.50(1-SD)**	**1.32(1.00-1.75)**	**0.051**

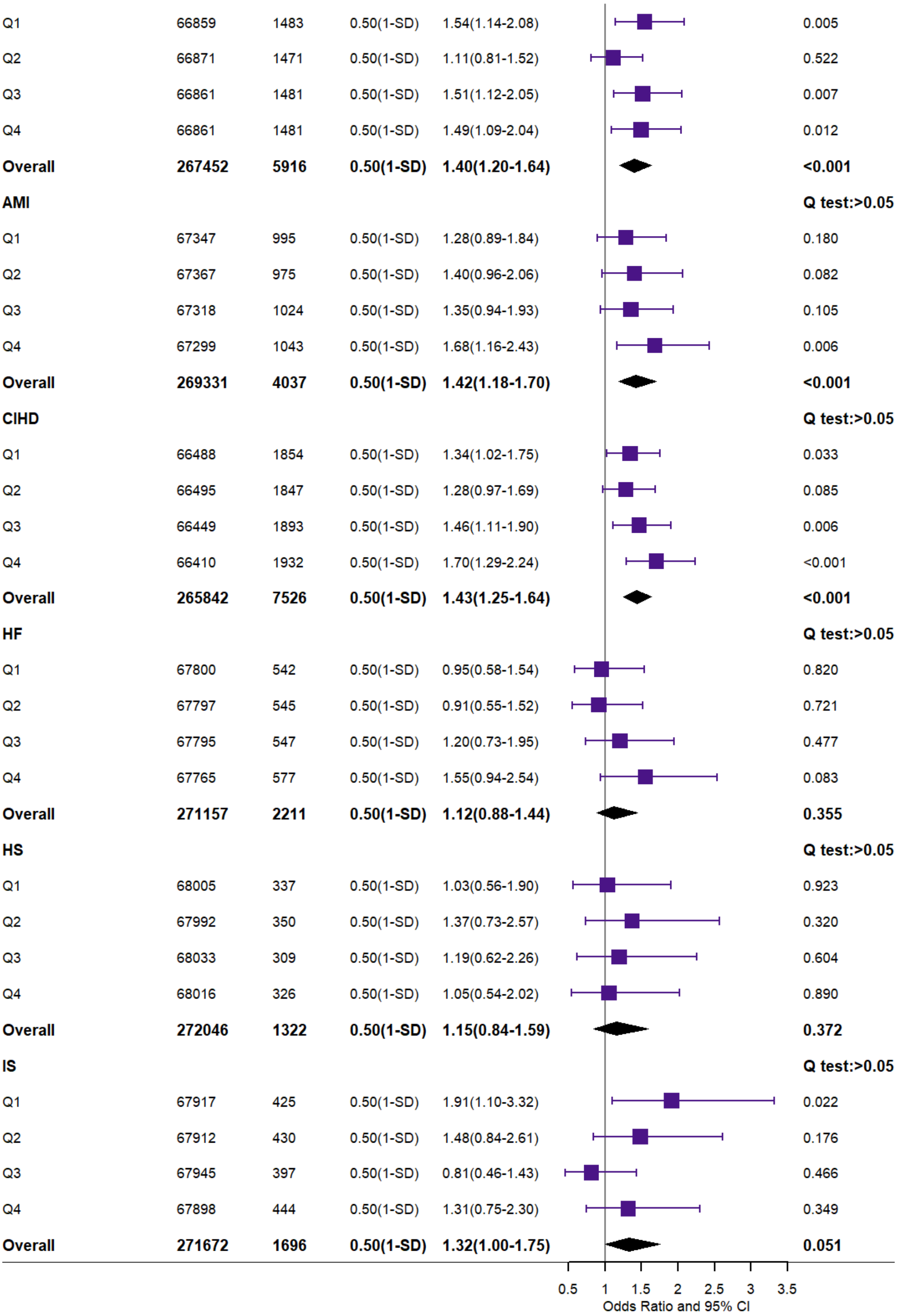

Figure S9. Assessment of independent higher TG in scaled difference with the risk of minor CVD by unweighted GRS in dataset A.

GLU by TG subgroup	No.Control	No.Case	ΔGLU	Odds Ratio	P value
**Ovrall CVD**					**Q test:>0.05**
Q1	77072	9697	0.70(1-SD)	1.25(0.95-1.65)	0.105
Q2	76629	10140	0.70(1-SD)	1.14(0.89-1.47)	0.302
Q3	76108	10661	0.70(1-SD)	0.94(0.73-1.21)	0.627
Q4	75600	11169	0.70(1-SD)	1.00(0.77-1.30)	0.999
**Overall**	**305409**	**41667**	**0.70(1-SD)**	**1.07(0.94-1.22)**	**0.317**
**IHD**					**Q test:>0.05**
Q1	79536	7233	0.70(1-SD)	1.43(1.05-1.95)	0.025
Q2	79021	7748	0.70(1-SD)	1.24(0.93-1.64)	0.141
Q3	78646	8123	0.70(1-SD)	0.97(0.73-1.29)	0.845
Q4	78093	8676	0.70(1-SD)	1.28(0.96-1.70)	0.097
**Overall**	**315296**	**31780**	**0.70(1-SD)**	**1.21(1.04-1.39)**	**0.012**
**CED**					**Q test:>0.05**
Q1	84022	2747	0.70(1-SD)	1.00(0.62-1.61)	0.998
Q2	84038	2731	0.70(1-SD)	0.75(0.48-1.18)	0.214
Q3	83856	2913	0.70(1-SD)	0.95(0.61-1.47)	0.819
Q4	83849	2920	0.70(1-SD)	0.69(0.44-1.09)	0.113
**Overall**	**335765**	**11311**	**0.70(1-SD)**	**0.84(0.67-1.05)**	**0.122**
**TG by GLU subgroup**	**No.Control**	**No.Case**	**Δlog(TG)**	**Odds Ratio**	**P value**
**CVD**					**Q test:>0.05**
Q1	76506	10263	0.50(1-SD)	1.63(1.45-1.84)	<0.001
Q2	76297	10472	0.50(1-SD)	1.47(1.31-1.66)	<0.001
Q3	76413	10356	0.50(1-SD)	1.40(1.24-1.58)	<0.001
Q4	76193	10576	0.50(1-SD)	1.43(1.27-1.62)	<0.001
**Overall**	**305409**	**41667**	**0.50(1-SD)**	**1.48(1.40-1.58)**	**<0.001**
**IHD**					**Q test:>0.05**
Q1	79049	7720	0.50(1-SD)	1.71(1.50-1.96)	<0.001
Q2	78808	7961	0.50(1-SD)	1.51(1.32-1.73)	<0.001
Q3	78826	7943	0.50(1-SD)	1.53(1.34-1.75)	<0.001
Q4	78613	8156	0.50(1-SD)	1.46(1.28-1.68)	<0.001
**Overall**	**315296**	**31780**	**0.50(1-SD)**	**1.55(1.45-1.66)**	**<0.001**
**CED**					**Q test:>0.05**
Q1	83889	2880	0.50(1-SD)	1.32(1.08-1.62)	0.007
Q2	83924	2845	0.50(1-SD)	1.22(0.99-1.51)	0.063
Q3	84000	2769	0.50(1-SD)	1.14(0.93-1.41)	0.213
Q4	83952	2817	0.50(1-SD)	1.34(1.09-1.66)	0.007
**Overall**	**335765**	**11311**	**0.50(1-SD)**	**1.26(1.13-1.40)**	**<0.001**

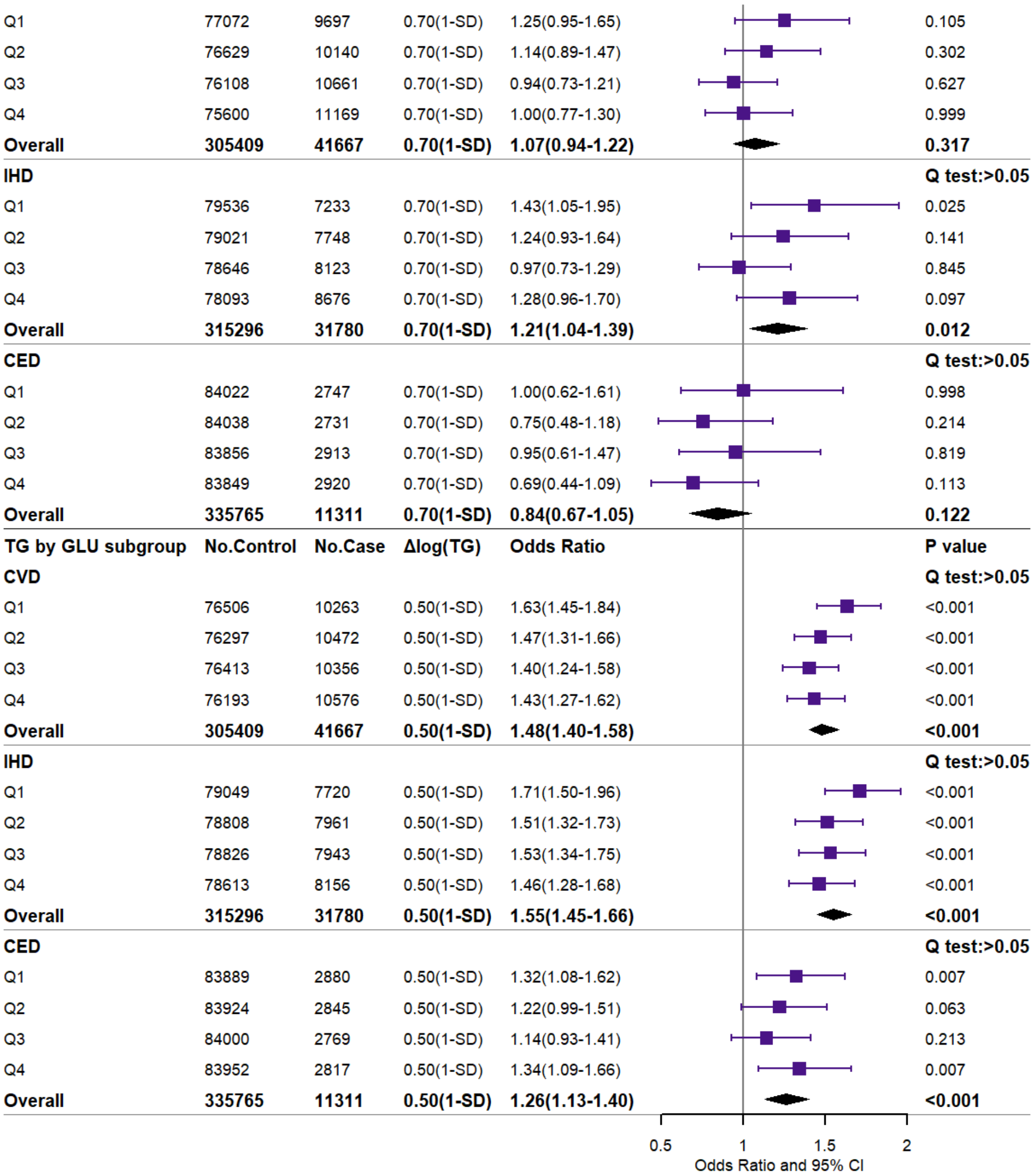

Figure S10. Assessment of independent higher GLU and TG in scaled difference with the risk of major CVD by unweighted GRS in dataset B.

GLU by TG subgroup	No.Control	No.Case	ΔGLU	Odds Ratio	P value
**AP**					**Q test:>0.05**
Q1	82152	4617	0.70(1-SD)	1.52(1.04-2.22)	0.030
Q2	81842	4927	0.70(1-SD)	1.64(1.16-2.32)	0.005
Q3	81538	5231	0.70(1-SD)	1.08(0.77-1.52)	0.651
Q4	81080	5689	0.70(1-SD)	1.30(0.92-1.84)	0.133
**Overall**	**326612**	**20464**	**0.70(1-SD)**	**1.36(1.14-1.62)**	**<0.001**
**AMI**					**Q test:>0.05**
Q1	83912	2857	0.70(1-SD)	1.17(0.73-1.87)	0.522
Q2	83761	3008	0.70(1-SD)	1.18(0.77-1.82)	0.454
Q3	83479	3290	0.70(1-SD)	1.09(0.71-1.65)	0.701
Q4	83281	3488	0.70(1-SD)	1.46(0.95-2.24)	0.084
**Overall**	**334433**	**12643**	**0.70(1-SD)**	**1.21(0.97-1.51)**	**0.086**
**CIHD**					**Q test:>0.05**
Q1	81229	5540	0.70(1-SD)	1.27(0.89-1.79)	0.186
Q2	80797	5972	0.70(1-SD)	1.20(0.88-1.66)	0.251
Q3	80430	6339	0.70(1-SD)	1.09(0.80-1.49)	0.602
Q4	80073	6696	0.70(1-SD)	1.15(0.83-1.58)	0.401
**Overall**	**322529**	**24547**	**0.70(1-SD)**	**1.17(0.99-1.37)**	**0.064**
**HF**					**Q test:>0.05**
Q1	85345	1424	0.70(1-SD)	0.92(0.48-1.76)	0.791
Q2	85348	1421	0.70(1-SD)	1.20(0.65-2.23)	0.555
Q3	85237	1532	0.70(1-SD)	1.08(0.59-1.96)	0.811
Q4	85273	1496	0.70(1-SD)	1.01(0.54-1.91)	0.970
**Overall**	**341203**	**5873**	**0.70(1-SD)**	**1.05(0.77-1.43)**	**0.776**
**HS**					**Q test:>0.05**
Q1	86255	514	0.70(1-SD)	2.51(0.87-7.29)	0.090
Q2	86221	548	0.70(1-SD)	0.62(0.24-1.64)	0.338
Q3	86202	567	0.70(1-SD)	0.80(0.31-2.09)	0.649
Q4	86265	504	0.70(1-SD)	0.69(0.23-2.03)	0.500
**Overall**	**344943**	**2133**	**0.70(1-SD)**	**0.95(0.57-1.57)**	**0.829**
**IS**					**Q test:>0.05**
Q1	85749	1020	0.70(1-SD)	0.57(0.26-1.21)	0.143
Q2	85749	1020	0.70(1-SD)	0.92(0.45-1.89)	0.826
Q3	85726	1043	0.70(1-SD)	2.11(1.03-4.32)	0.042
Q4	85614	1155	0.70(1-SD)	0.73(0.35-1.49)	0.384
**Overall**	**342838**	**4238**	**0.70(1-SD)**	**0.95(0.66-1.36)**	**0.769**
**DM**					**Q test:>0.05**
Q1	82377	4392	0.70(1-SD)	5.33(3.57-7.96)	<0.001
Q2	82110	4659	0.70(1-SD)	3.80(2.63-5.48)	<0.001
Q3	81799	4970	0.70(1-SD)	4.63(3.23-6.64)	<0.001
Q4	81309	5460	0.70(1-SD)	5.16(3.58-7.42)	<0.001
**Overall**	**327595**	**19481**	**0.70(1-SD)**	**4.66(3.87-5.61)**	**<0.001**
**DLM**					**Q test:>0.05**
Q1	73058	13711	0.70(1-SD)	1.19(0.94-1.52)	0.154
Q2	71630	15139	0.70(1-SD)	1.08(0.86-1.34)	0.511
Q3	69906	16863	0.70(1-SD)	1.38(1.12-1.71)	0.003
Q4	67848	18921	0.70(1-SD)	1.20(0.97-1.48)	0.099
**Overall**	**282442**	**64634**	**0.70(1-SD)**	**1.20(1.07-1.34)**	**0.001**

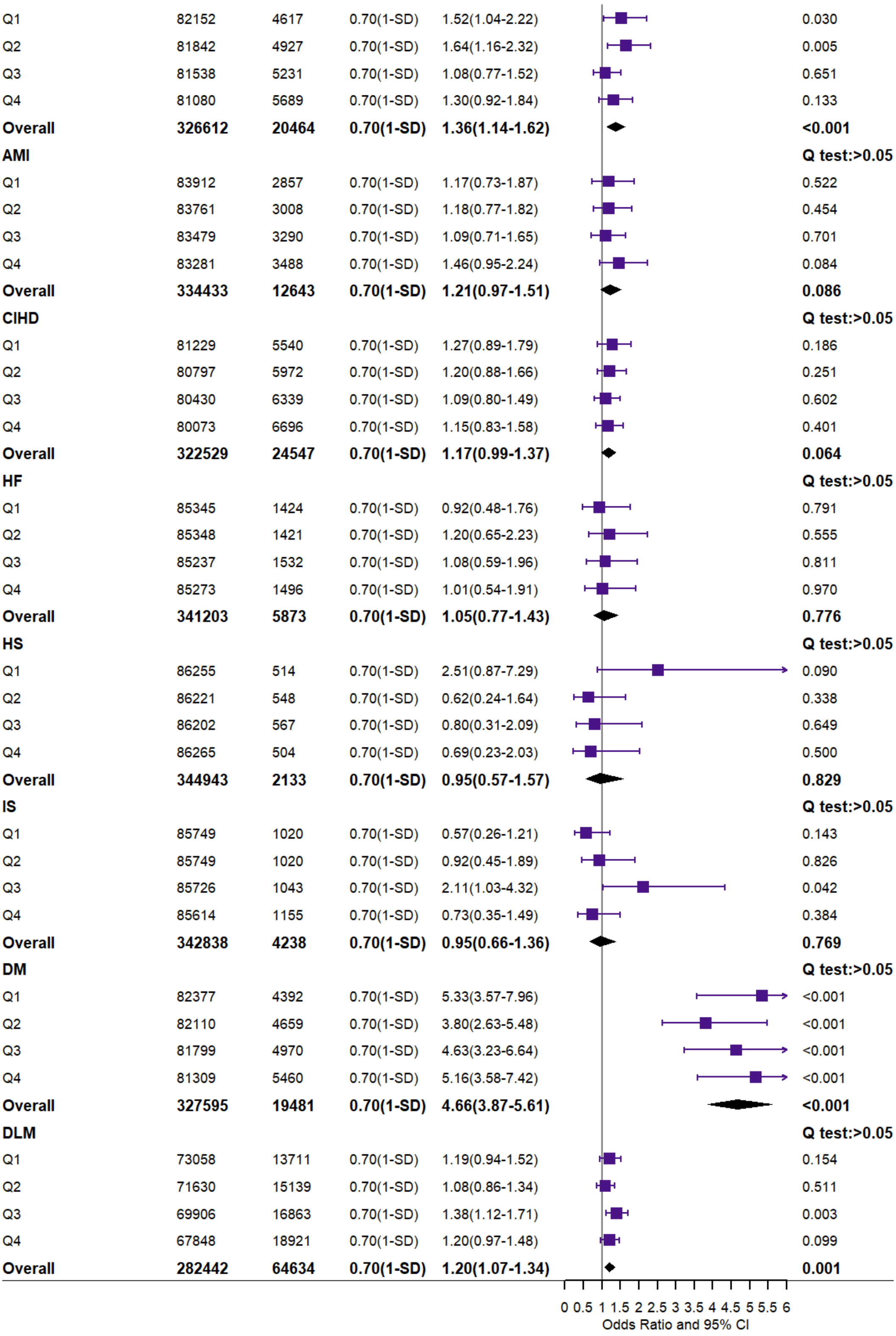

Figure S11. Assessment of independent higher GLU in scaled difference with the risk of minor CVD by unweighted GRS in dataset B.

TG by GLU subgroup	No.Control	No.Case	Δlog(TG)	Odds Ratio	P value
**AP**					**Q test:>0.05**
Q1	81890	4879	0.50(1-SD)	1.80(1.53-2.12)	<0.001
Q2	81644	5125	0.50(1-SD)	1.68(1.42-1.98)	<0.001
Q3	81658	5111	0.50(1-SD)	1.66(1.41-1.95)	<0.001
Q4	81420	5349	0.50(1-SD)	1.41(1.20-1.66)	<0.001
**Overall**	**326612**	**20464**	**0.50(1-SD)**	**1.63(1.50-1.76)**	**<0.001**
**AMI**					**Q test:>0.05**
Q1	83712	3057	0.50(1-SD)	1.72(1.41-2.11)	<0.001
Q2	83607	3162	0.50(1-SD)	1.49(1.21-1.83)	<0.001
Q3	83676	3093	0.50(1-SD)	1.60(1.31-1.96)	<0.001
Q4	83438	3331	0.50(1-SD)	1.73(1.42-2.11)	<0.001
**Overall**	**334433**	**12643**	**0.50(1-SD)**	**1.63(1.48-1.81)**	**<0.001**
**CIHD**					**Q test:>0.05**
Q1	80780	5989	0.50(1-SD)	1.68(1.45-1.95)	<0.001
Q2	80630	6139	0.50(1-SD)	1.53(1.32-1.79)	<0.001
Q3	80639	6130	0.50(1-SD)	1.56(1.35-1.82)	<0.001
Q4	80480	6289	0.50(1-SD)	1.52(1.31-1.77)	<0.001
**Overall**	**322529**	**24547**	**0.50(1-SD)**	**1.57(1.46-1.70)**	**<0.001**
**HF**					**Q test:>0.05**
Q1	85342	1427	0.50(1-SD)	1.24(0.93-1.65)	0.142
Q2	85268	1501	0.50(1-SD)	1.27(0.95-1.70)	0.109
Q3	85328	1441	0.50(1-SD)	1.29(0.97-1.73)	0.083
Q4	85265	1504	0.50(1-SD)	1.22(0.91-1.63)	0.187
**Overall**	**341203**	**5873**	**0.50(1-SD)**	**1.26(1.09-1.45)**	**0.002**
**HS**					**Q test:>0.05**
Q1	86234	535	0.50(1-SD)	1.21(0.76-1.91)	0.423
Q2	86235	534	0.50(1-SD)	1.10(0.68-1.76)	0.704
Q3	86240	529	0.50(1-SD)	0.80(0.50-1.29)	0.365
Q4	86234	535	0.50(1-SD)	1.08(0.67-1.74)	0.753
**Overall**	**344943**	**2133**	**0.50(1-SD)**	**1.04(0.82-1.31)**	**0.764**
**IS**					**Q test:>0.05**
Q1	85734	1035	0.50(1-SD)	1.08(0.77-1.51)	0.650
Q2	85680	1089	0.50(1-SD)	1.17(0.84-1.64)	0.356
Q3	85721	1048	0.50(1-SD)	1.58(1.13-2.22)	0.008
Q4	85703	1066	0.50(1-SD)	1.30(0.92-1.82)	0.135
**Overall**	**342838**	**4238**	**0.50(1-SD)**	**1.27(1.07-1.51)**	**0.005**
**DM**					**Q test:>0.05**
Q1	82613	4156	0.50(1-SD)	1.89(1.58-2.27)	<0.001
Q2	82202	4567	0.50(1-SD)	1.63(1.36-1.95)	<0.001
Q3	81744	5025	0.50(1-SD)	1.75(1.48-2.07)	<0.001
Q4	81036	5733	0.50(1-SD)	1.88(1.60-2.22)	<0.001
**Overall**	**327595**	**19481**	**0.50(1-SD)**	**1.78(1.63-1.94)**	**<0.001**
**DLM**					**Q test:>0.05**
Q1	70694	16075	0.50(1-SD)	2.38(2.15-2.63)	<0.001
Q2	70853	15916	0.50(1-SD)	2.48(2.23-2.75)	<0.001
Q3	70591	16178	0.50(1-SD)	2.57(2.32-2.85)	<0.001
Q4	70304	16465	0.50(1-SD)	2.63(2.37-2.92)	<0.001
**Overall**	**282442**	**64634**	**0.50(1-SD)**	**2.52(2.39-2.65)**	**<0.001**

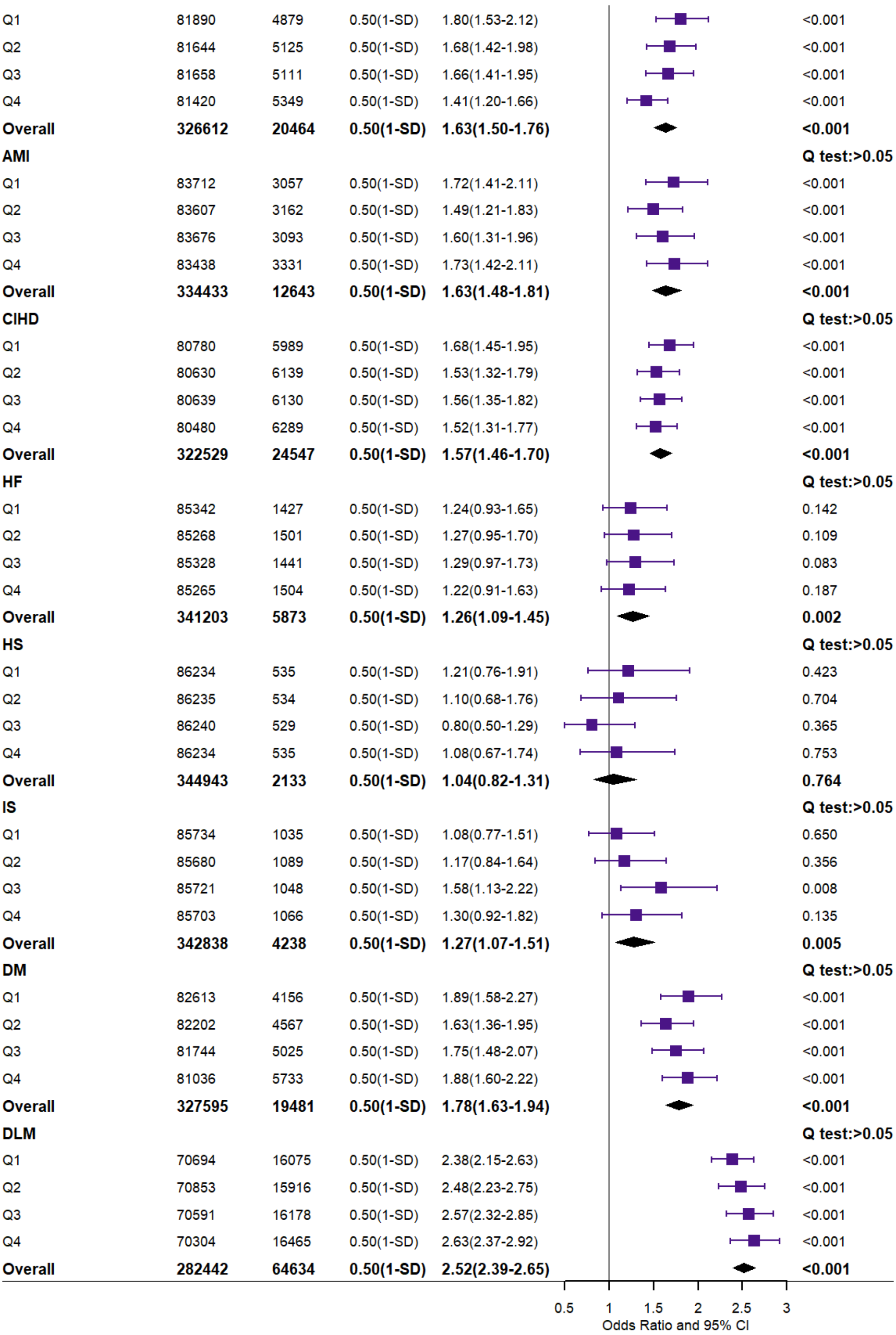

Figure S12. Assessment of independent higher TG in scaled difference with the risk of minor CVD by unweighted GRS in dataset B.

GLU by TG subgroup	No.Control	No.Case	ΔGLU	Odds Ratio	P value
**Ovrall CVD**					**Q test:>0.05**
Q1	64288	4093	0.62(1-SD)	1.40(0.92-2.13)	0.116
Q2	64239	4120	0.62(1-SD)	1.34(0.78-2.29)	0.288
Q3	64212	4075	0.62(1-SD)	1.34(0.86-2.08)	0.197
Q4	64082	4259	0.62(1-SD)	0.96(0.60-1.52)	0.853
**Overall**	**256821**	**16547**	**0.62(1-SD)**	**1.24(0.99-1.57)**	**0.064**
**IHD**					**Q test:>0.05**
Q1	65685	2696	0.62(1-SD)	1.46(0.88-2.43)	0.146
Q2	65650	2709	0.62(1-SD)	1.37(0.71-2.63)	0.344
Q3	65566	2721	0.62(1-SD)	1.41(0.83-2.39)	0.210
Q4	65429	2912	0.62(1-SD)	1.03(0.59-1.79)	0.914
**Overall**	**262330**	**11038**	**0.62(1-SD)**	**1.30(0.98-1.72)**	**0.066**
**CED**					**Q test:>0.05**
Q1	67076	1305	0.62(1-SD)	1.42(0.69-2.91)	0.336
Q2	67030	1329	0.62(1-SD)	0.76(0.31-1.90)	0.564
Q3	66998	1289	0.62(1-SD)	0.93(0.44-1.98)	0.855
Q4	67050	1291	0.62(1-SD)	1.11(0.50-2.49)	0.797
**Overall**	**268154**	**5214**	**0.62(1-SD)**	**1.05(0.71-1.56)**	**0.811**
**TG by GLU subgroup**	**No.Control**	**No.Case**	**Δlog(TG)**	**Odds Ratio**	**P value**
**CVD**					**Q test:>0.05**
Q1	64256	4086	0.50(1-SD)	1.46(1.22-1.75)	<0.001
Q2	64224	4119	0.50(1-SD)	1.21(1.02-1.44)	0.029
Q3	64143	4198	0.50(1-SD)	1.31(1.10-1.56)	0.003
Q4	64198	4144	0.50(1-SD)	1.19(1.00-1.41)	0.049
**Overall**	**256821**	**16547**	**0.50(1-SD)**	**1.29(1.18-1.40)**	**<0.001**
**IHD**					**Q test:>0.05**
Q1	65626	2716	0.50(1-SD)	1.52(1.23-1.89)	<0.001
Q2	65600	2743	0.50(1-SD)	1.35(1.10-1.67)	0.005
Q3	65541	2800	0.50(1-SD)	1.45(1.17-1.79)	<0.001
Q4	65563	2779	0.50(1-SD)	1.27(1.03-1.56)	0.025
**Overall**	**262330**	**11038**	**0.50(1-SD)**	**1.39(1.25-1.55)**	**<0.001**
**CED**					**Q test:>0.05**
Q1	67020	1322	0.50(1-SD)	1.35(0.99-1.82)	0.055
Q2	67052	1291	0.50(1-SD)	0.98(0.73-1.32)	0.876
Q3	67005	1336	0.50(1-SD)	1.15(0.86-1.56)	0.347
Q4	67077	1265	0.50(1-SD)	1.09(0.81-1.47)	0.564
**Overall**	**268154**	**5214**	**0.50(1-SD)**	**1.14(0.98-1.32)**	**0.097**

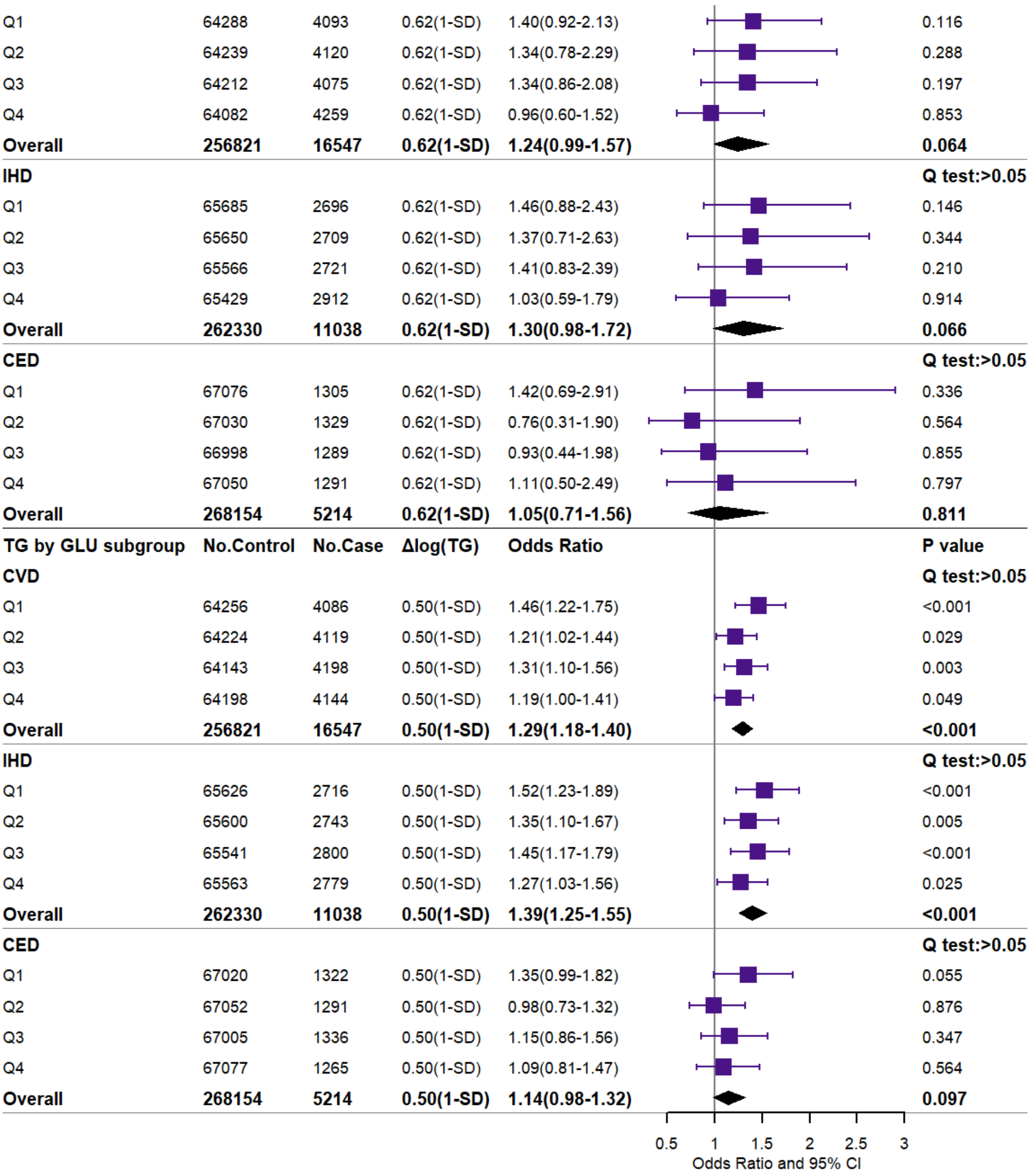

Figure S13. Assessment of independent higher GLU and TG in scaled difference with the risk of major CVD by weighted external reported SNPs in dataset A.

GLU by TG subgroup	No.Control	No.Case	ΔGLU	Odds Ratio	P value
**AP**					**Q test:>0.05**
Q1	66927	1454	0.62(1-SD)	1.28(0.65-2.54)	0.478
Q2	66943	1416	0.62(1-SD)	1.26(0.52-3.06)	0.614
Q3	66841	1446	0.62(1-SD)	2.26(1.10-4.64)	0.026
Q4	66741	1600	0.62(1-SD)	1.11(0.53-2.30)	0.785
**Overall**	**267452**	**5916**	**0.62(1-SD)**	**1.41(0.97-2.05)**	**0.073**
**AMI**					**Q test:>0.05**
Q1	67440	941	0.62(1-SD)	1.18(0.50-2.74)	0.708
Q2	67396	963	0.62(1-SD)	1.75(0.60-5.10)	0.304
Q3	67282	1005	0.62(1-SD)	2.61(1.11-6.16)	0.028
Q4	67213	1128	0.62(1-SD)	0.82(0.35-1.95)	0.653
**Overall**	**269331**	**4037**	**0.62(1-SD)**	**1.41(0.90-2.21)**	**0.136**
**CIHD**					**Q test:>0.05**
Q1	66596	1785	0.62(1-SD)	1.86(1.00-3.47)	0.050
Q2	66493	1866	0.62(1-SD)	1.32(0.61-2.88)	0.481
Q3	66433	1854	0.62(1-SD)	0.96(0.51-1.82)	0.901
Q4	66320	2021	0.62(1-SD)	1.07(0.56-2.07)	0.831
**Overall**	**265842**	**7526**	**0.62(1-SD)**	**1.26(0.90-1.75)**	**0.182**
**HF**					**Q test:>0.05**
Q1	67854	527	0.62(1-SD)	1.74(0.57-5.37)	0.333
Q2	67809	550	0.62(1-SD)	1.42(0.35-5.82)	0.623
Q3	67713	574	0.62(1-SD)	0.74(0.24-2.29)	0.606
Q4	67781	560	0.62(1-SD)	0.86(0.25-2.90)	0.807
**Overall**	**271157**	**2211**	**0.62(1-SD)**	**1.11(0.60-2.02)**	**0.746**
**HS**					**Q test:>0.05**
Q1	68064	317	0.62(1-SD)	1.44(0.34- 6.01)	0.620
Q2	67996	363	0.62(1-SD)	0.88(0.16- 4.90)	0.889
Q3	67980	307	0.62(1-SD)	2.35(0.51-10.81)	0.274
Q4	68006	335	0.62(1-SD)	0.53(0.11- 2.52)	0.424
**Overall**	**272046**	**1322**	**0.62(1-SD)**	**1.13(0.52- 2.45)**	**0.756**
**IS**					**Q test:>0.05**
Q1	67972	409	0.62(1-SD)	1.97(0.56-7.00)	0.292
Q2	67956	403	0.62(1-SD)	0.35(0.07-1.79)	0.207
Q3	67842	445	0.62(1-SD)	0.87(0.24-3.09)	0.828
Q4	67902	439	0.62(1-SD)	0.76(0.19-2.99)	0.694
**Overall**	**271672**	**1696**	**0.62(1-SD)**	**0.87(0.44-1.72)**	**0.681**

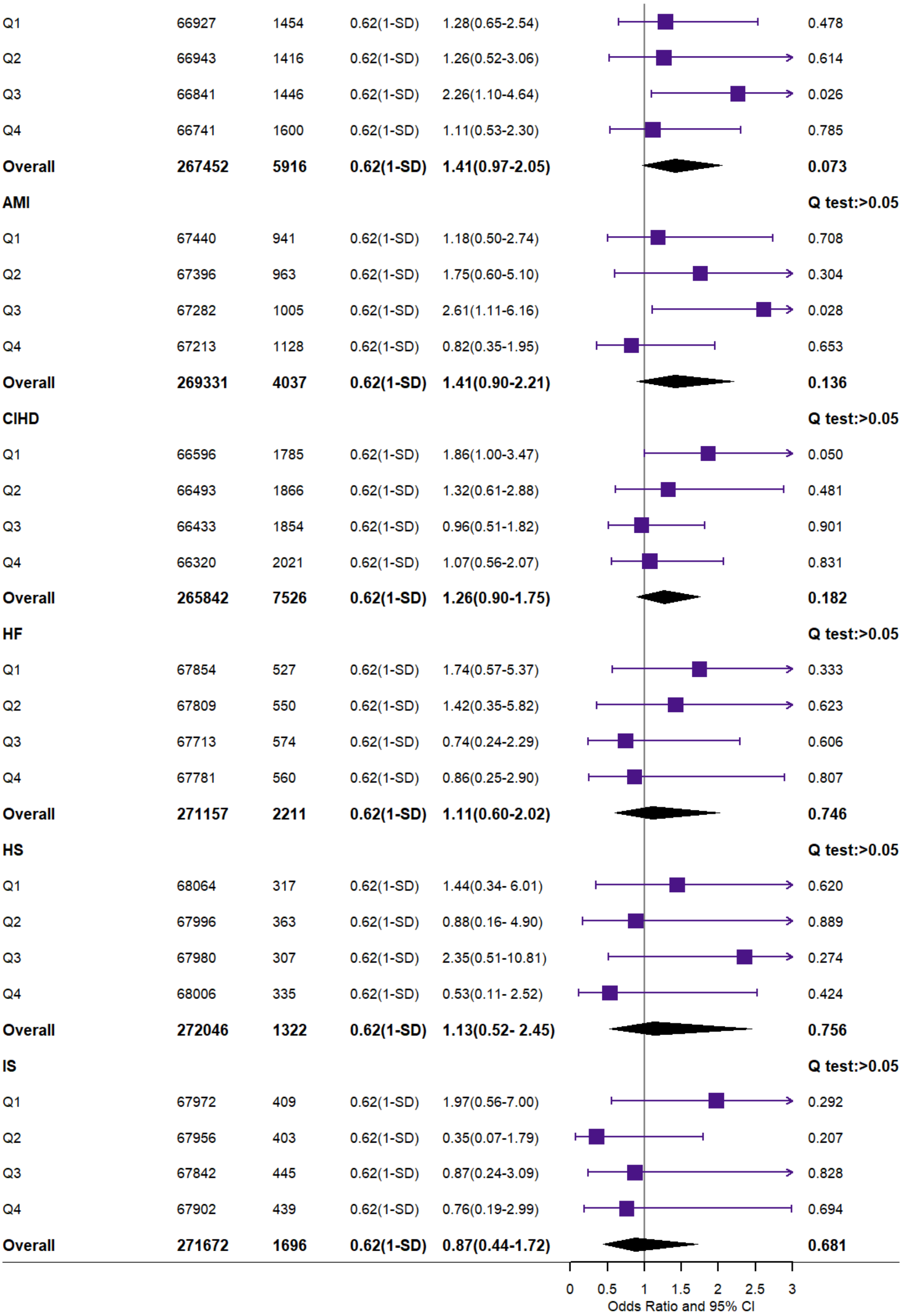

Figure S14. Assessment of independent higher GLU in scaled difference with the risk of minor CVD by weighted external reported SNPs in dataset A.

TG by GLU subgroup	No.Control	No.Case	Δlog(TG)	Odds Ratio	P value
**AP**					**Q test:>0.05**
Q1	66868	1474	0.50(1-SD)	1.61(1.20-2.14)	0.001
Q2	66901	1442	0.50(1-SD)	1.30(0.98-1.73)	0.070
Q3	66817	1524	0.50(1-SD)	1.72(1.29-2.28)	<0.001
Q4	66866	1476	0.50(1-SD)	1.46(1.11-1.93)	0.008
**Overall**	**267452**	**5916**	**0.50(1-SD)**	**1.51(1.31-1.74)**	**<0.001**
**AMI**					**Q test:>0.05**
Q1	67351	991	0.50(1-SD)	1.61(1.14-2.29)	0.007
Q2	67353	990	0.50(1-SD)	1.84(1.31-2.59)	<0.001
Q3	67335	1006	0.50(1-SD)	1.88(1.33-2.66)	<0.001
Q4	67292	1050	0.50(1-SD)	1.61(1.16-2.24)	0.005
**Overall**	**269331**	**4037**	**0.50(1-SD)**	**1.73(1.46-2.05)**	**<0.001**
**CIHD**					**Q test:>0.05**
Q1	66500	1842	0.50(1-SD)	1.69(1.30-2.19)	<0.001
Q2	66459	1884	0.50(1-SD)	1.49(1.16-1.92)	0.002
Q3	66477	1864	0.50(1-SD)	1.46(1.13-1.89)	0.004
Q4	66406	1936	0.50(1-SD)	1.24(0.97-1.58)	0.088
**Overall**	**265842**	**7526**	**0.50(1-SD)**	**1.46(1.28-1.65)**	**<0.001**
**HF**					**Q test:>0.05**
Q1	67787	555	0.50(1-SD)	1.52(0.95-2.42)	0.079
Q2	67795	548	0.50(1-SD)	1.69(1.07-2.65)	0.024
Q3	67784	557	0.50(1-SD)	1.51(0.95-2.39)	0.083
Q4	67791	551	0.50(1-SD)	1.04(0.66-1.64)	0.864
**Overall**	**271157**	**2211**	**0.50(1-SD)**	**1.41(1.12-1.77)**	**0.003**
**HS**					**Q test:>0.05**
Q1	68028	314	0.50(1-SD)	0.74(0.40-1.36)	0.325
Q2	67998	345	0.50(1-SD)	1.08(0.61-1.92)	0.781
Q3	67982	359	0.50(1-SD)	0.83(0.47-1.46)	0.516
Q4	68038	304	0.50(1-SD)	1.00(0.55-1.83)	0.996
**Overall**	**272046**	**1322**	**0.50(1-SD)**	**0.90(0.67-1.21)**	**0.498**
**IS**					**Q test:>0.05**
Q1	67897	445	0.50(1-SD)	2.14(1.27-3.59)	0.004
Q2	67925	418	0.50(1-SD)	1.07(0.64-1.80)	0.797
Q3	67911	430	0.50(1-SD)	1.21(0.72-2.04)	0.472
Q4	67939	403	0.50(1-SD)	1.72(1.02-2.90)	0.043
**Overall**	**271672**	**1696**	**0.50(1-SD)**	**1.48(1.14-1.92)**	**0.003**

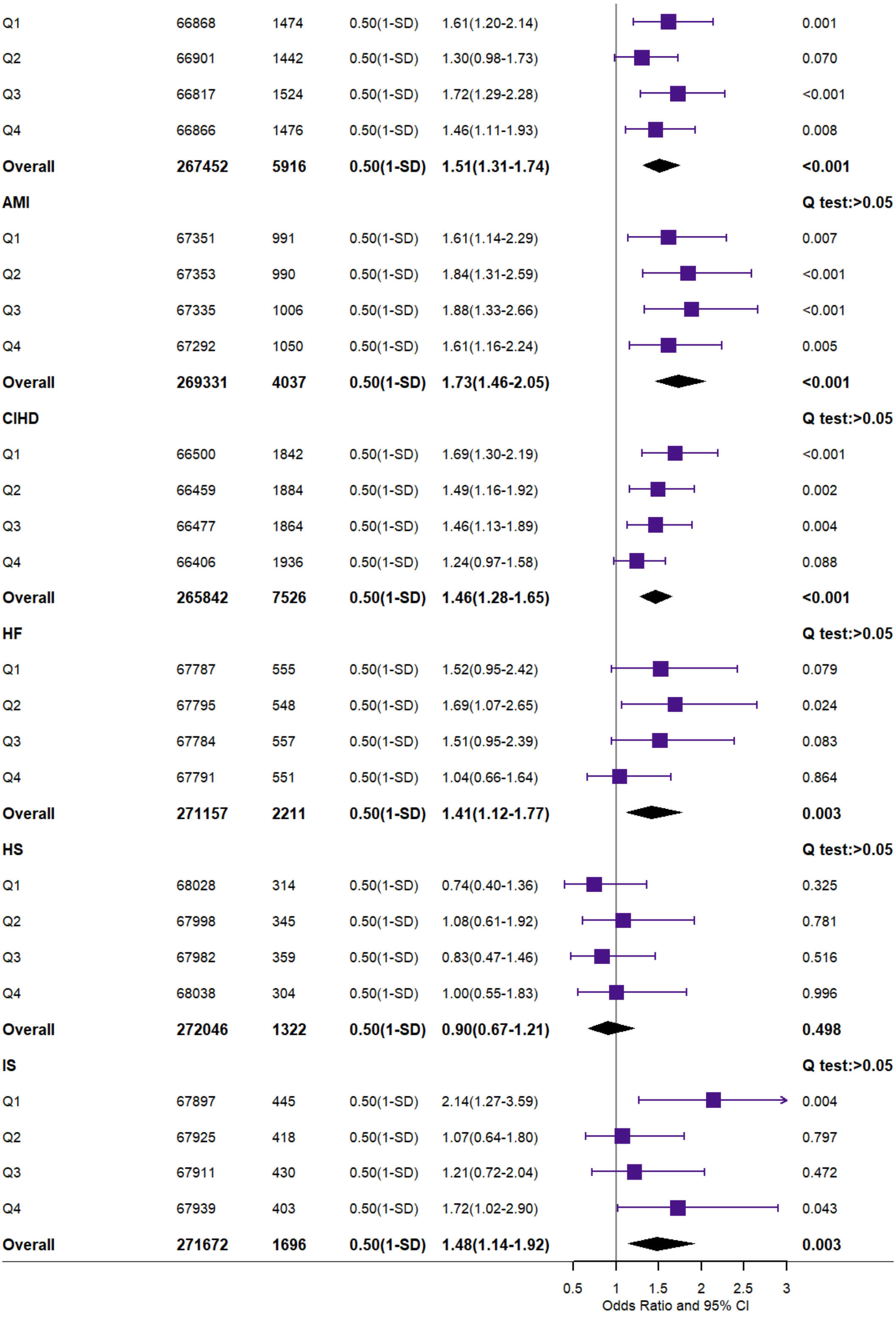

Figure S15. Assessment of independent higher TG in scaled difference with the risk of minor CVD by weighted external reported SNPs in dataset A.

GLU by TG subgroup	No.Control	No.Case	ΔGLU	Odds Ratio	P value
**Ovrall CVD**					**Q test:>0.05**
Q1	65204	3210	0.62(1-SD)	0.95(0.90-1.00)	0.063
Q2	64302	3970	0.62(1-SD)	0.96(0.91-1.01)	0.080
Q3	63910	4501	0.62(1-SD)	0.97(0.93-1.01)	0.182
Q4	63405	4866	0.62(1-SD)	0.99(0.96-1.03)	0.746
**Overall**	**256821**	**16547**	**0.62(1-SD)**	**0.97(0.95-0.99)**	**0.017**
**IHD**					**Q test:>0.05**
Q1	66425	1989	0.62(1-SD)	0.96(0.90-1.03)	0.231
Q2	65670	2602	0.62(1-SD)	0.96(0.90-1.02)	0.183
Q3	65339	3072	0.62(1-SD)	0.99(0.94-1.04)	0.721
Q4	64896	3375	0.62(1-SD)	1.00(0.96-1.05)	0.888
**Overall**	**262330**	**11038**	**0.62(1-SD)**	**0.98(0.96-1.01)**	**0.256**
**CED**					**Q test:>0.05**
Q1	67266	1148	0.62(1-SD)	0.93(0.86-1.02)	0.133
Q2	66979	1293	0.62(1-SD)	0.95(0.87-1.03)	0.223
Q3	67049	1362	0.62(1-SD)	0.93(0.86-1.00)	0.053
Q4	66860	1411	0.62(1-SD)	0.97(0.90-1.03)	0.321
**Overall**	**268154**	**5214**	**0.62(1-SD)**	**0.95(0.91-0.99)**	**0.007**
**TG by GLU subgroup**	**No.Control**	**No.Case**	**Δlog(TG)**	**Odds Ratio**	**P value**
**CVD**					**Q test:>0.05**
Q1	64434	3955	0.50(1-SD)	1.08(1.03-1.13)	<0.001
Q2	64530	3864	0.50(1-SD)	1.13(1.08-1.18)	<0.001
Q3	64285	4075	0.50(1-SD)	1.14(1.09-1.20)	<0.001
Q4	63572	4653	0.50(1-SD)	1.13(1.08-1.17)	<0.001
**Overall**	**256821**	**16547**	**0.50(1-SD)**	**1.12(1.10-1.15)**	**<0.001**
**IHD**					**Q test:>0.05**
Q1	65794	2595	0.50(1-SD)	1.10(1.04-1.16)	<0.001
Q2	65854	2540	0.50(1-SD)	1.17(1.10-1.23)	<0.001
Q3	65618	2742	0.50(1-SD)	1.19(1.12-1.25)	<0.001
Q4	65064	3161	0.50(1-SD)	1.18(1.12-1.24)	<0.001
**Overall**	**262330**	**11038**	**0.50(1-SD)**	**1.16(1.13-1.19)**	**<0.001**
**CED**					**Q test:>0.05**
Q1	67098	1291	0.50(1-SD)	1.05(0.97-1.13)	0.245
Q2	67132	1262	0.50(1-SD)	1.10(1.02-1.19)	0.018
Q3	67084	1276	0.50(1-SD)	1.07(0.99-1.16)	0.086
Q4	66840	1385	0.50(1-SD)	1.07(1.00-1.15)	0.067
**Overall**	**268154**	**5214**	**0.50(1-SD)**	**1.07(1.03-1.11)**	**<0.001**

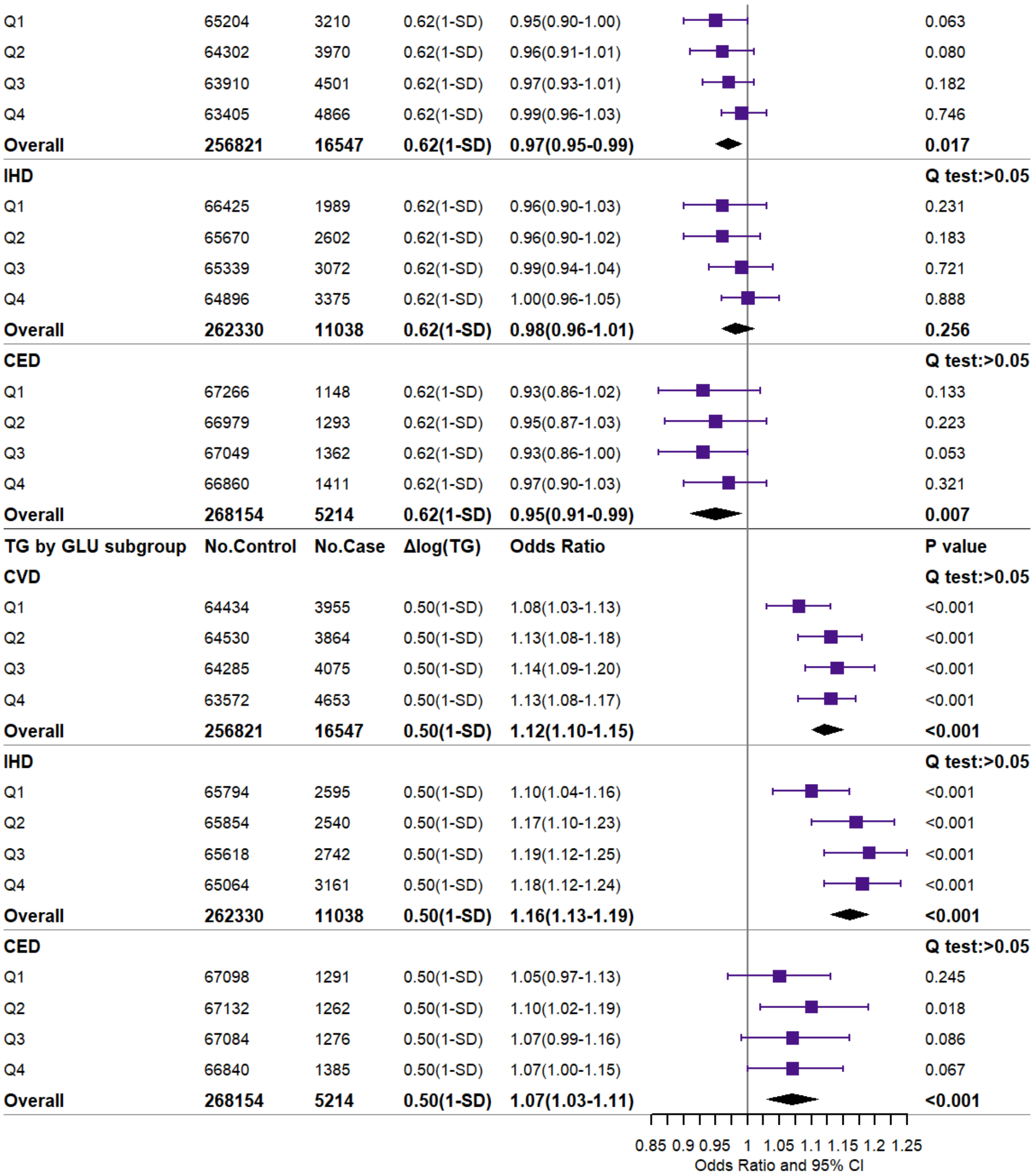

Figure S16. Assessment of independent higher GLU and TG in scaled difference with the risk of major CVD by observational phenotypes in dataset A.

GLU by TG subgroup	No.Control	No.Case	ΔGLU	Odds Ratio	P value
**AP**					**Q test:>0.05**
Q1	67341	1073	0.62(1-SD)	0.94(0.86-1.03)	0.189
Q2	66919	1353	0.62(1-SD)	0.98(0.90-1.06)	0.557
Q3	66736	1675	0.62(1-SD)	1.02(0.95-1.09)	0.607
Q4	66456	1815	0.62(1-SD)	1.05(0.99-1.12)	0.098
**Overall**	**267452**	**5916**	**0.62(1-SD)**	**1.01(0.97-1.05)**	**0.633**
**AMI**					**Q test:>0.05**
Q1	67722	692	0.62(1-SD)	1.00(0.89-1.12)	0.934
Q2	67309	963	0.62(1-SD)	0.97(0.88-1.06)	0.487
Q3	67307	1104	0.62(1-SD)	1.00(0.92-1.09)	0.948
Q4	66993	1278	0.62(1-SD)	1.02(0.95-1.10)	0.590
**Overall**	**269331**	**4037**	**0.62(1-SD)**	**1.00(0.95-1.04)**	**0.909**
**CIHD**					**Q test:>0.05**
Q1	67069	1345	0.62(1-SD)	0.98(0.90-1.07)	0.661
Q2	66494	1778	0.62(1-SD)	0.93(0.87-1.00)	0.046
Q3	66324	2087	0.62(1-SD)	0.97(0.91-1.04)	0.385
Q4	65955	2316	0.62(1-SD)	0.98(0.93-1.03)	0.458
**Overall**	**265842**	**7526**	**0.62(1-SD)**	**0.97(0.94-1.00)**	**0.052**
**HF**					**Q test:>0.05**
Q1	67956	458	0.62(1-SD)	0.93(0.81-1.07)	0.320
Q2	67762	510	0.62(1-SD)	0.89(0.78-1.01)	0.066
Q3	67799	612	0.62(1-SD)	0.98(0.88-1.10)	0.786
Q4	67640	631	0.62(1-SD)	1.06(0.96-1.18)	0.268
**Overall**	**271157**	**2211**	**0.62(1-SD)**	**0.98(0.92-1.04)**	**0.444**
**HS**					**Q test:>0.05**
Q1	68106	308	0.62(1-SD)	0.89(0.75-1.06)	0.186
Q2	67940	332	0.62(1-SD)	0.95(0.81-1.12)	0.570
Q3	68085	326	0.62(1-SD)	1.01(0.86-1.18)	0.904
Q4	67915	356	0.62(1-SD)	1.02(0.89-1.17)	0.761
**Overall**	**272046**	**1322**	**0.62(1-SD)**	**0.97(0.90-1.05)**	**0.502**
**IS**					**Q test:>0.05**
Q1	68055	359	0.62(1-SD)	0.93(0.80-1.09)	0.388
Q2	67878	394	0.62(1-SD)	0.88(0.76-1.02)	0.078
Q3	67968	443	0.62(1-SD)	0.92(0.81-1.05)	0.222
Q4	67771	500	0.62(1-SD)	0.90(0.80-1.01)	0.079
**Overall**	**271672**	**1696**	**0.62(1-SD)**	**0.91(0.85-0.97)**	**0.005**

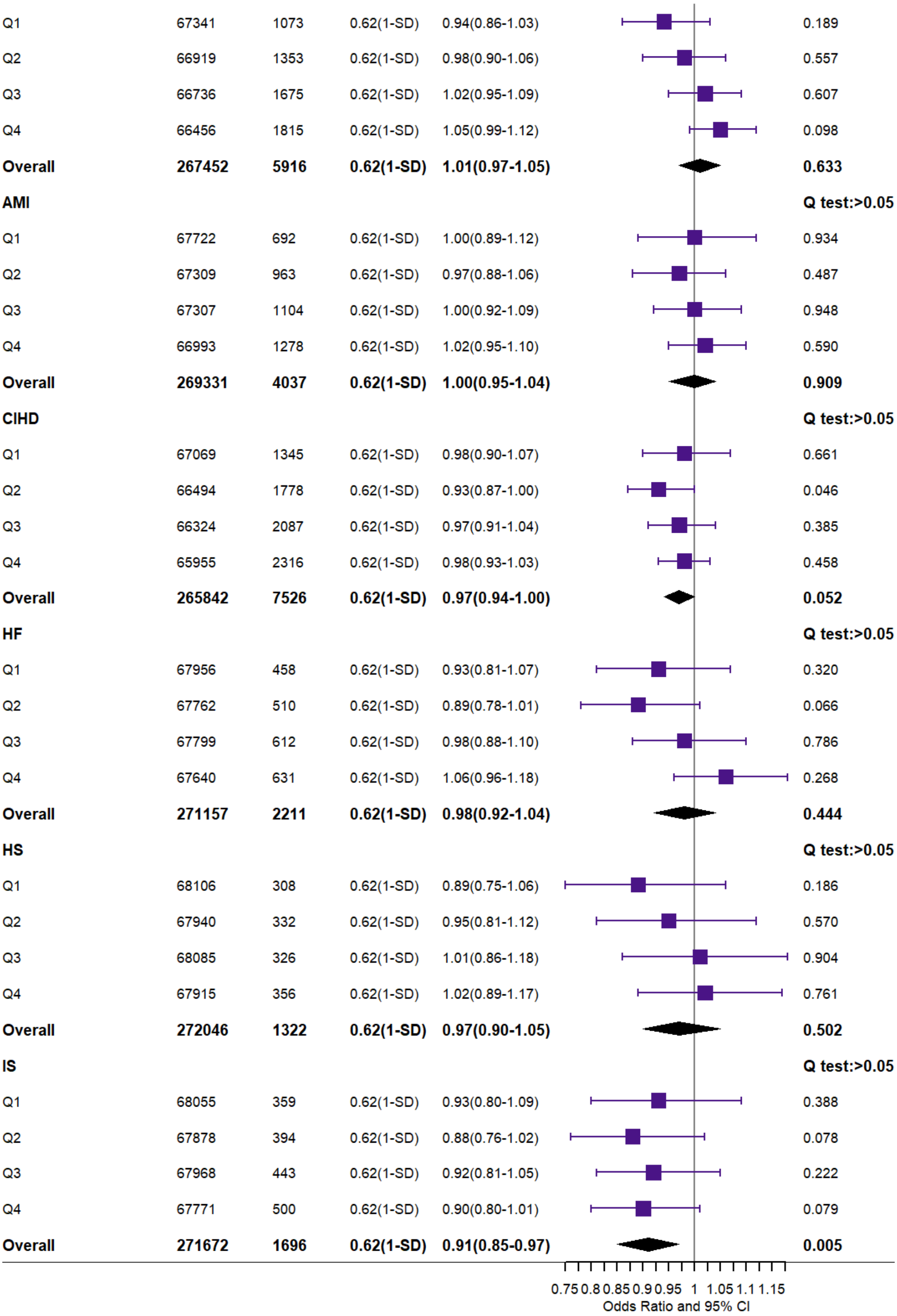

Figure S17. Assessment of independent higher GLU in scaled difference with the risk of minor CVD by observational phenotypes in dataset A.

TG by GLU subgroup	No.Control	No.Case	Δlog(TG)	Odds Ratio	P value
**AP**					**Q test:>0.05**
Q1	67058	1331	0.50(1-SD)	1.16(1.07-1.24)	<0.001
Q2	67036	1358	0.50(1-SD)	1.21(1.12-1.30)	<0.001
Q3	66895	1465	0.50(1-SD)	1.28(1.19-1.37)	<0.001
Q4	66463	1762	0.50(1-SD)	1.26(1.18-1.34)	<0.001
**Overall**	**267452**	**5916**	**0.50(1-SD)**	**1.23(1.19-1.27)**	**<0.001**
**AMI**					**Q test:>0.05**
Q1	67388	1001	0.50(1-SD)	1.12(1.03-1.22)	0.007
Q2	67514	880	0.50(1-SD)	1.22(1.11-1.33)	<0.001
Q3	67352	1008	0.50(1-SD)	1.21(1.11-1.33)	<0.001
Q4	67077	1148	0.50(1-SD)	1.20(1.11-1.30)	<0.001
**Overall**	**269331**	**4037**	**0.50(1-SD)**	**1.19(1.14-1.24)**	**<0.001**
**CIHD**					**Q test:>0.05**
Q1	66570	1819	0.50(1-SD)	1.10(1.03-1.17)	0.004
Q2	66680	1714	0.50(1-SD)	1.16(1.08-1.24)	<0.001
Q3	66526	1834	0.50(1-SD)	1.17(1.10-1.25)	<0.001
Q4	66066	2159	0.50(1-SD)	1.13(1.07-1.20)	<0.001
**Overall**	**265842**	**7526**	**0.50(1-SD)**	**1.14(1.11-1.18)**	**<0.001**
**HF**					**Q test:>0.05**
Q1	67890	499	0.50(1-SD)	1.01(0.89-1.13)	0.918
Q2	67875	519	0.50(1-SD)	0.97(0.87-1.10)	0.673
Q3	67837	523	0.50(1-SD)	1.13(1.00-1.28)	0.044
Q4	67555	670	0.50(1-SD)	1.00(0.90-1.11)	0.950
**Overall**	**271157**	**2211**	**0.50(1-SD)**	**1.03(0.97-1.09)**	**0.338**
**HS**					**Q test:>0.05**
Q1	68054	335	0.50(1-SD)	0.94(0.81-1.09)	0.423
Q2	68076	318	0.50(1-SD)	1.01(0.87-1.17)	0.918
Q3	68043	317	0.50(1-SD)	1.17(1.00-1.36)	0.053
Q4	67873	352	0.50(1-SD)	1.02(0.89-1.18)	0.755
**Overall**	**272046**	**1322**	**0.50(1-SD)**	**1.03(0.96-1.11)**	**0.408**
**IS**					**Q test:>0.05**
Q1	67969	420	0.50(1-SD)	1.01(0.89-1.15)	0.878
Q2	67972	422	0.50(1-SD)	1.15(1.01-1.31)	0.037
Q3	67948	412	0.50(1-SD)	1.06(0.93-1.22)	0.380
Q4	67783	442	0.50(1-SD)	1.06(0.93-1.20)	0.395
**Overall**	**271672**	**1696**	**0.50(1-SD)**	**1.07(1.00-1.14)**	**0.042**

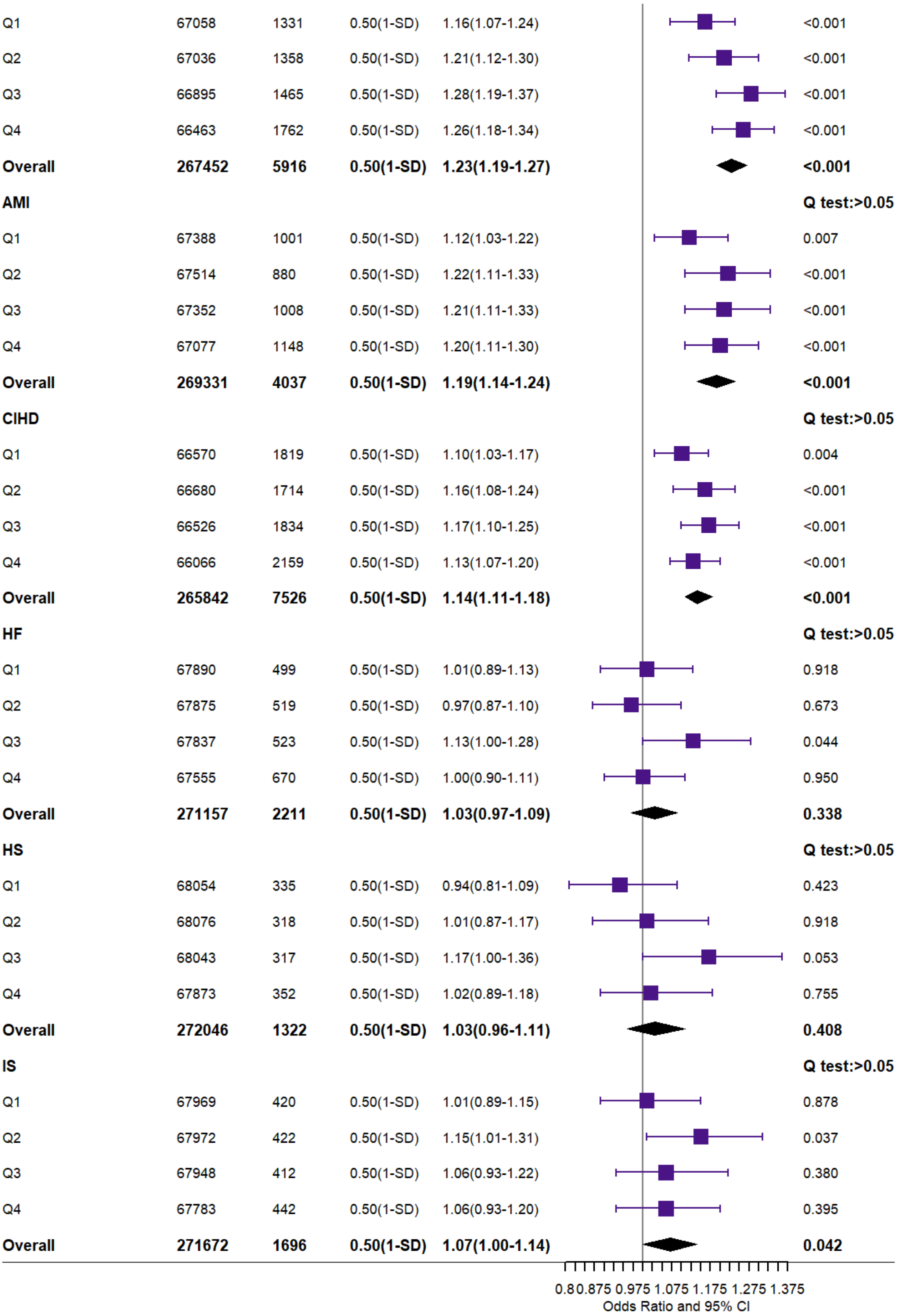

Figure S18. Assessment of independent higher TG in scaled difference with the risk of minor CVD by observational phenotypes in dataset A.

2×2 subgroup	No.control	No.case	ΔGLU	Δlog(TG)	Odds Ratio	P value
**Major CVD events**						
**Overall CVD**						
Reference	64037	4027				
Higher GLU	64530	4090	0.62(1-SD)	0.00	1.22(1.06-1.40)	0.002
Higher TG	64466	4154	0.00	0.50(1-SD)	1.33(1.23-1.43)	<0.001
Higher GLU and TG	63788	4276	0.62(1-SD)	0.50(1-SD)	1.61(1.44-1.80)	<0.001
**IHD**						
Reference	65425	2639				
Higher GLU	65864	2756	0.62(1-SD)	0.00	1.31(1.20-1.44)	<0.001
Higher TG	65886	2734	0.00	0.50(1-SD)	1.37(1.31-1.45)	<0.001
Higher GLU and TG	65155	2909	0.62(1-SD)	0.50(1-SD)	1.80(1.68-1.94)	<0.001
**CED**						
Reference	66753	1311				
Higher GLU	67358	1262	0.62(1-SD)	0.00	1.08(0.77-1.51)	0.334
Higher TG	67281	1339	0.00	0.50(1-SD)	1.29(1.07-1.56)	0.004
Higher GLU and TG	66762	1302	0.62(1-SD)	0.50(1-SD)	1.39(1.06-1.83)	0.009
**Minor CVD events**						
**AP**						
Reference	66655	1409				
Higher GLU	67139	1481	0.62(1-SD)	0.00	1.20(1.10-1.32)	<0.001
Higher TG	67142	1478	0.00	0.50(1-SD)	1.41(1.34-1.48)	<0.001
Higher GLU and TG	66516	1548	0.62(1-SD)	0.50(1-SD)	1.70(1.57-1.83)	<0.001
**AMI**						
Reference	67146	918				
Higher GLU	67643	977	0.62(1-SD)	0.00	1.34(1.17-1.55)	<0.001
Higher TG	67605	1015	0.00	0.50(1-SD)	1.49(1.38-1.62)	<0.001
Higher GLU and TG	66937	1127	0.62(1-SD)	0.50(1-SD)	2.01(1.79-2.25)	<0.001
**CIHD**						
Reference	66281	1783				
Higher GLU	66730	1890	0.62(1-SD)	0.00	1.45(1.27-1.65)	<0.001
Higher TG	66749	1871	0.00	0.50(1-SD)	1.42(1.32-1.53)	<0.001
Higher GLU and TG	66082	1982	0.62(1-SD)	0.50(1-SD)	2.05(1.84-2.28)	<0.001
**HF**						
Reference	67517	547				
Higher GLU	68090	530	0.62(1-SD)	0.00	1.08(0.74-1.58)	0.336
Higher TG	68063	557	0.00	0.50(1-SD)	1.24(1.00-1.53)	0.024
Higher GLU and TG	67487	577	0.62(1-SD)	0.50(1-SD)	1.34(0.99-1.82)	0.029
**HS**						
Reference	67721	343				
Higher GLU	68309	311	0.62(1-SD)	0.00	1.10(0.79-1.54)	0.288
Higher TG	68286	334	0.00	0.50(1-SD)	1.19(0.99-1.43)	0.032
Higher GLU and TG	67730	334	0.62(1-SD)	0.50(1-SD)	1.31(1.00-1.72)	0.025
**IS**						
Reference	67649	415				
Higher GLU	68214	406	0.62(1-SD)	0.00	1.09(0.78-1.53)	0.308
Higher TG	68175	445	0.00	0.50(1-SD)	1.37(1.13-1.65)	<0.001
Higher GLU and TG	67634	430	0.62(1-SD)	0.50(1-SD)	1.49(1.13-1.96)	0.002

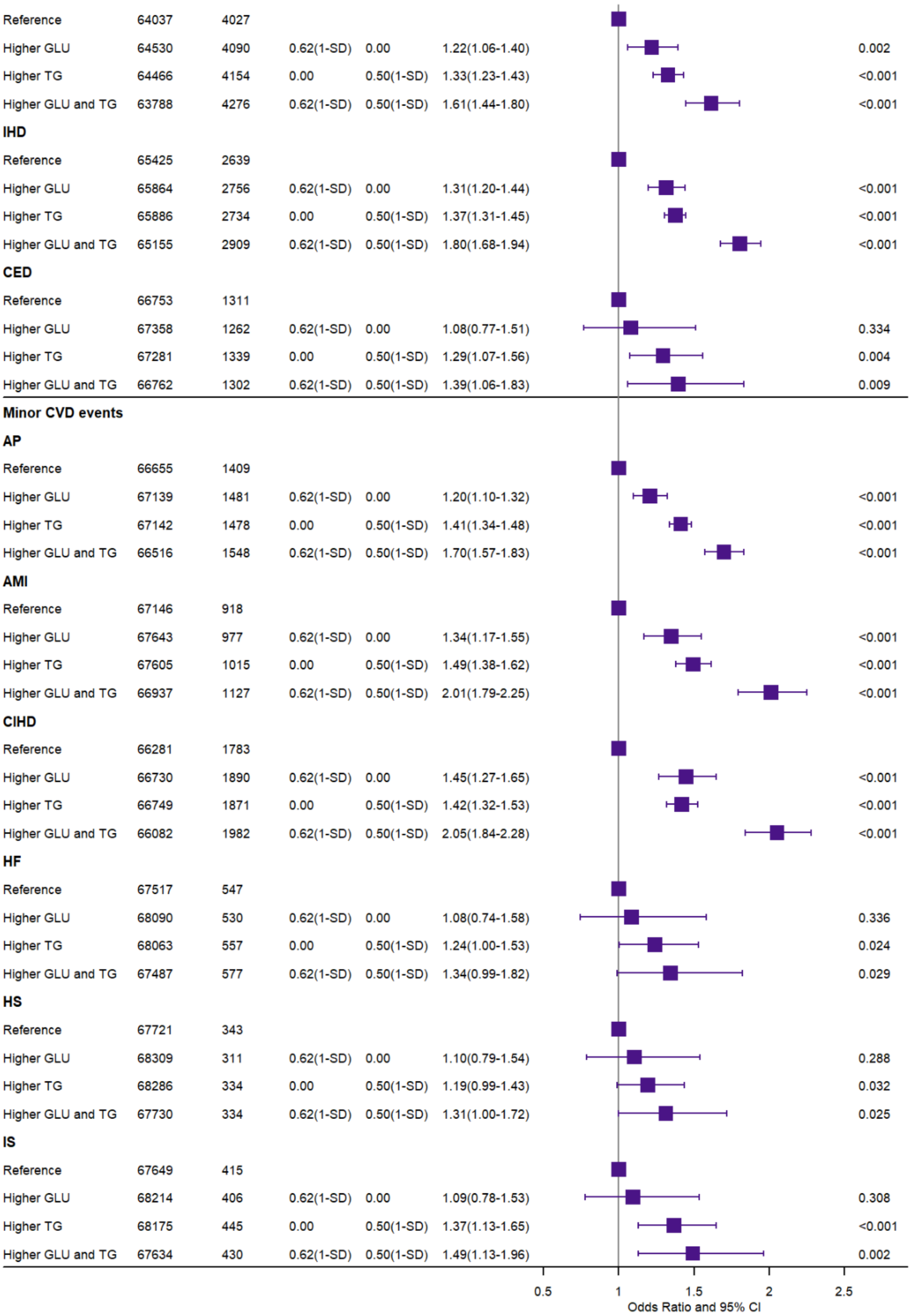

Figure S19. Association of combined exposure to GLU and TG in scaled difference with the risk of CVD by weighted GRS in dataset A.

2×2 subgroup	No.control	No.case	ΔGLU	Δlog(TG)	Odds Ratio	P value
**Major CVD events**						
**Overall CVD**						
Reference	63563	4053				
Higher GLU	64982	4086	0.134	0.007	1.00(0.96-1.05)	0.939
Higher TG	64843	4225	-0.001	0.173	1.09(1.04-1.14)	<0.001
Higher GLU and TG	63433	4183	0.128	0.178	1.13(1.08-1.18)	<0.001
**IHD**						
Reference	64942	2674				
Higher GLU	66334	2734	0.134	0.007	1.02(0.96-1.08)	0.502
Higher TG	66290	2778	-0.001	0.173	1.08(1.03-1.15)	0.004
Higher GLU and TG	64764	2852	0.128	0.178	1.18(1.11-1.24)	<0.001
**CED**						
Reference	66327	1289				
Higher GLU	67782	1286	0.134	0.007	0.99(0.91-1.07)	0.756
Higher TG	67674	1394	-0.001	0.173	1.12(1.04-1.21)	0.004
Higher GLU and TG	66371	1245	0.128	0.178	1.04(0.96-1.12)	0.365
**Minor CVD events**						
**AP**						
Reference	66168	1448				
Higher GLU	67606	1462	0.134	0.007	1.01(0.94-1.09)	0.830
Higher TG	67562	1506	-0.001	0.173	1.10(1.02-1.19)	0.012
Higher GLU and TG	66116	1500	0.128	0.178	1.16(1.07-1.25)	<0.001
**AMI**						
Reference	66657	959				
Higher GLU	68057	1011	0.134	0.007	1.06(0.97-1.16)	0.220
Higher TG	68057	1011	-0.001	0.173	1.11(1.01-1.21)	0.030
Higher GLU and TG	66560	1056	0.128	0.178	1.22(1.11-1.33)	<0.001
**CIHD**						
Reference	65814	1802				
Higher GLU	67212	1856	0.134	0.007	1.03(0.96-1.10)	0.412
Higher TG	67169	1899	-0.001	0.173	1.10(1.03-1.17)	0.007
Higher GLU and TG	65647	1969	0.128	0.178	1.20(1.12-1.28)	<0.001
**HF**						
Reference	67057	559				
Higher GLU	68511	557	0.134	0.007	1.00(0.89-1.13)	0.974
Higher TG	68540	528	-0.001	0.173	0.98(0.87-1.10)	0.720
Higher GLU and TG	67049	567	0.128	0.178	1.11(0.98-1.25)	0.089
**HS**						
Reference	67284	332				
Higher GLU	68749	319	0.134	0.007	0.94(0.81-1.10)	0.439
Higher TG	68713	355	-0.001	0.173	1.06(0.91-1.24)	0.427
Higher GLU and TG	67300	316	0.128	0.178	0.97(0.83-1.14)	0.742
**IS**						
Reference	67218	398				
Higher GLU	68633	435	0.134	0.007	1.09(0.95-1.25)	0.208
Higher TG	68611	457	-0.001	0.173	1.19(1.04-1.37)	0.011
Higher GLU and TG	67210	406	0.128	0.178	1.11(0.96-1.28)	0.148

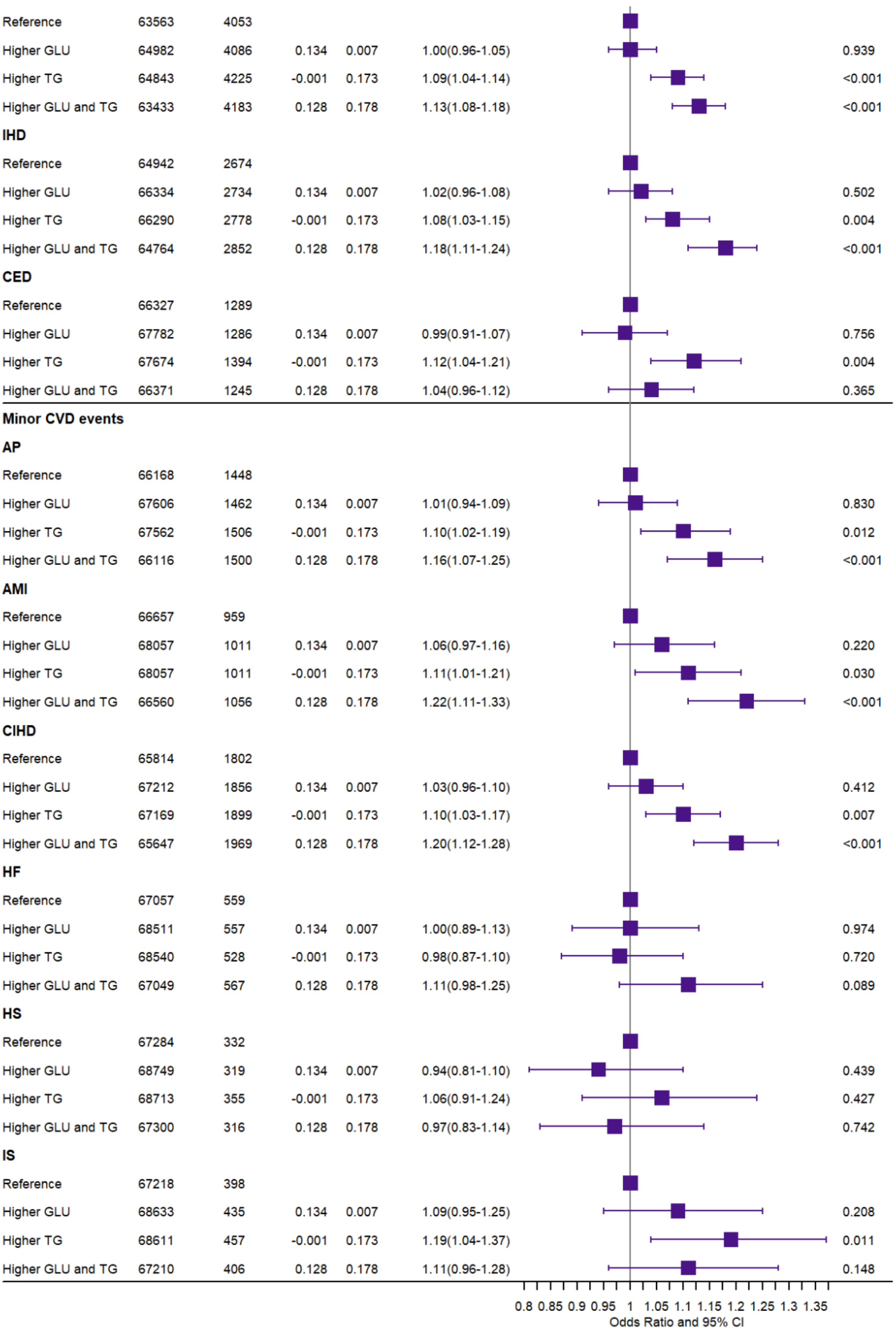

Figure S20. Association of combined exposure to GLU and TG in crude difference with the risk of CVD by unweighted GRS in dataset A.

2×2 subgroup	No.control	No.case	ΔGLU	Δlog(TG)	Odds Ratio	P value
**Major CVD events**						
**Overall CVD**						
Reference	76537	9771				
Higher GLU	77164	10066	0.119	0.007	1.03(1.00-1.06)	0.063
Higher TG	76266	10964	0.007	0.183	1.18(1.14-1.21)	<0.001
Higher GLU and TG	75442	10866	0.125	0.189	1.17(1.13-1.21)	<0.001
**IHD**						
Reference	78986	7322				
Higher GLU	79571	7659	0.119	0.007	1.05(1.01-1.09)	0.009
Higher TG	78871	8359	0.007	0.183	1.19(1.15-1.23)	<0.001
Higher GLU and TG	77868	8440	0.125	0.189	1.21(1.17-1.26)	<0.001
**CED**						
Reference	83550	2758				
Higher GLU	84510	2720	0.119	0.007	0.98(0.92-1.03)	0.382
Higher TG	84263	2967	0.007	0.183	1.09(1.04-1.15)	0.001
Higher GLU and TG	83442	2866	0.125	0.189	1.06(1.00-1.11)	0.053
**Minor CVD events**						
**AP**						
Reference	81708	4600				
Higher GLU	82286	4944	0.119	0.007	1.08(1.03-1.13)	<0.001
Higher TG	81826	5404	0.007	0.183	1.22(1.17-1.28)	<0.001
Higher GLU and TG	80792	5516	0.125	0.189	1.26(1.21-1.31)	<0.001
**AMI**						
Reference	83422	2886				
Higher GLU	84251	2979	0.119	0.007	1.03(0.97-1.08)	0.341
Higher TG	83897	3333	0.007	0.183	1.19(1.13-1.25)	<0.001
Higher GLU and TG	82863	3445	0.125	0.189	1.23(1.17-1.30)	<0.001
**CIHD**						
Reference	80659	5649				
Higher GLU	81367	5863	0.119	0.007	1.04(1.00-1.08)	0.087
Higher TG	80751	6479	0.007	0.183	1.19(1.14-1.24)	<0.001
Higher GLU and TG	79752	6556	0.125	0.189	1.21(1.16-1.26)	<0.001
**HF**						
Reference	84890	1418				
Higher GLU	85803	1427	0.119	0.007	1.01(0.93-1.09)	0.828
Higher TG	85720	1510	0.007	0.183	1.09(1.01-1.17)	0.029
Higher GLU and TG	84790	1518	0.125	0.189	1.10(1.02-1.18)	0.017
**HS**						
Reference	85789	519				
Higher GLU	86687	543	0.119	0.007	1.03(0.91-1.16)	0.616
Higher TG	86680	550	0.007	0.183	1.05(0.93-1.19)	0.389
Higher GLU and TG	85787	521	0.125	0.189	1.00(0.89-1.13)	0.954
**IS**						
Reference	85265	1043				
Higher GLU	86233	997	0.119	0.007	0.95(0.87-1.03)	0.227
Higher TG	86149	1081	0.007	0.183	1.04(0.96-1.14)	0.324
Higher GLU and TG	85191	1117	0.125	0.189	1.08(0.99-1.18)	0.080
**Basic disease**						
**DM**						
Reference	82258	4050				
Higher GLU	82229	5001	0.119	0.007	1.29(1.23-1.35)	<0.001
Higher TG	82557	4673	0.007	0.183	1.23(1.17-1.28)	<0.001
Higher GLU and TG	80551	5757	0.125	0.189	1.60(1.53-1.67)	<0.001
**DLM**						
Reference	72016	14292				
Higher GLU	72672	14558	0.119	0.007	1.02(0.99-1.05)	0.147
Higher TG	69531	17699	0.007	0.183	1.38(1.35-1.42)	<0.001
Higher GLU and TG	68223	18085	0.125	0.189	1.44(1.41-1.48)	<0.001

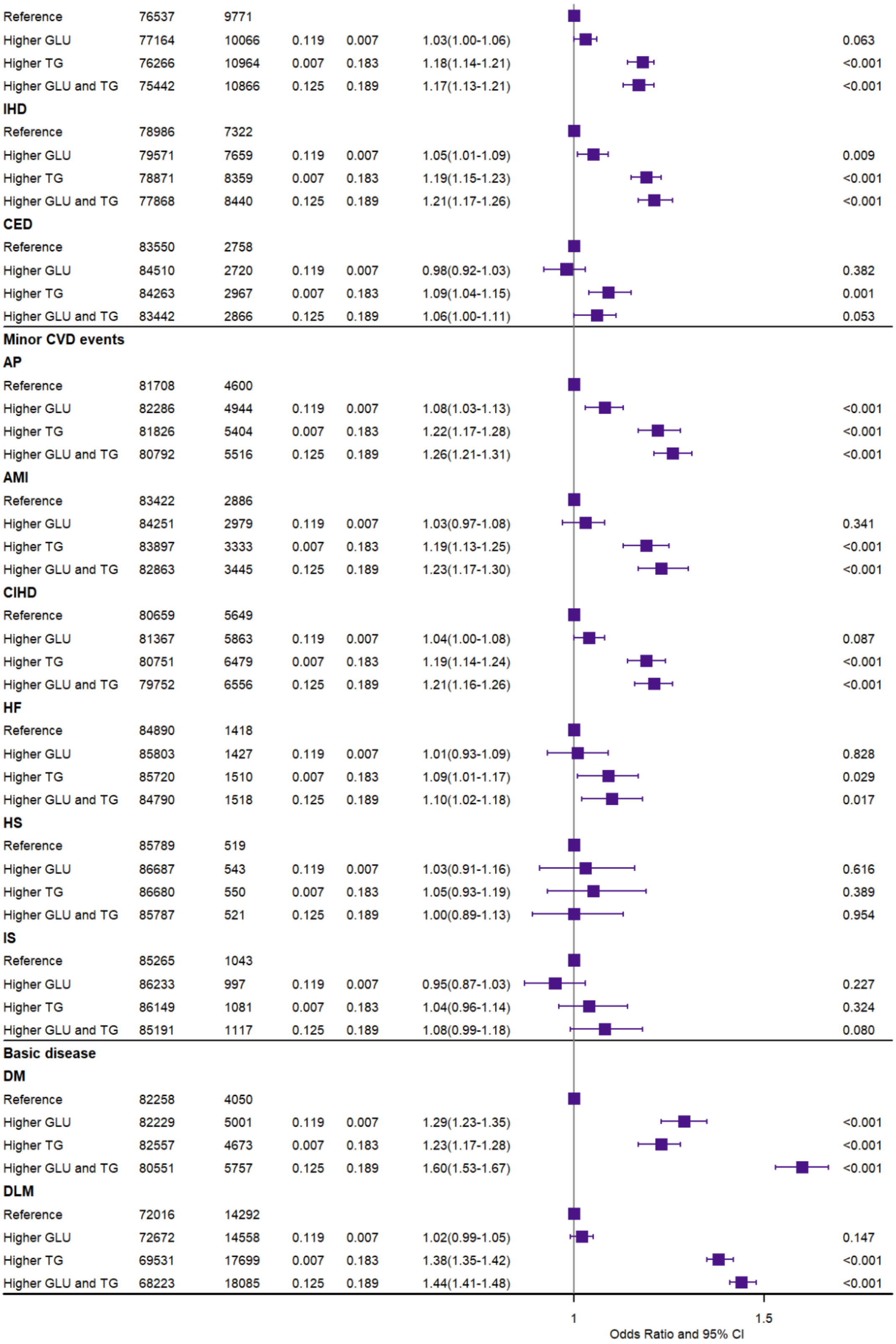

Figure S21. Association of combined exposure to GLU and TG in crude difference with the risk of CVD by weighted GRS in dataset B.

2×2 subgroup	No.control	No.case	ΔGLU	Δlog(TG)	Odds Ratio	P value
**Major CVD events**						
**Overall CVD**						
Reference	64305	4035				
Higher GLU	64222	4178	0.085	0.006	1.04(1.00-1.09)	0.061
Higher TG	64175	4170	-0.014	0.185	1.11(1.06-1.17)	<0.001
Higher GLU and TG	64119	4164	0.075	0.192	1.13(1.08-1.19)	<0.001
**IHD**						
Reference	65690	2650				
Higher GLU	65645	2755	0.085	0.006	1.05(0.99-1.11)	0.088
Higher TG	65536	2809	-0.014	0.185	1.14(1.08-1.21)	<0.001
Higher GLU and TG	65459	2824	0.075	0.192	1.17(1.11-1.24)	<0.001
**CED**						
Reference	67027	1313				
Higher GLU	67079	1321	0.085	0.006	1.01(0.93-1.09)	0.793
Higher TG	67045	1300	-0.014	0.185	1.05(0.97-1.14)	0.206
Higher GLU and TG	67003	1280	0.075	0.192	1.06(0.98-1.14)	0.179
**Minor CVD events**						
**AP**						
Reference	66921	1419				
Higher GLU	66949	1451	0.085	0.006	1.03(0.96-1.11)	0.397
Higher TG	66848	1497	-0.014	0.185	1.15(1.06-1.24)	<0.001
Higher GLU and TG	66734	1549	0.075	0.192	1.22(1.14-1.32)	<0.001
**AMI**						
Reference	67408	932				
Higher GLU	67428	972	0.085	0.006	1.05(0.96-1.15)	0.318
Higher TG	67296	1049	-0.014	0.185	1.22(1.12-1.34)	<0.001
Higher GLU and TG	67199	1084	0.075	0.192	1.29(1.18-1.41)	<0.001
**CIHD**						
Reference	66564	1776				
Higher GLU	66525	1875	0.085	0.006	1.07(1.00-1.14)	0.058
Higher TG	66395	1950	-0.014	0.185	1.19(1.11-1.27)	<0.001
Higher GLU and TG	66358	1925	0.075	0.192	1.19(1.11-1.27)	<0.001
**HF**						
Reference	67818	522				
Higher GLU	67845	555	0.085	0.006	1.07(0.94-1.21)	0.296
Higher TG	67764	581	-0.014	0.185	1.19(1.06-1.35)	0.004
Higher GLU and TG	67730	553	0.075	0.192	1.15(1.02-1.30)	0.021
**HS**						
Reference	68003	337				
Higher GLU	68057	343	0.085	0.006	1.02(0.88-1.18)	0.818
Higher TG	68023	322	-0.014	0.185	0.96(0.83-1.12)	0.639
Higher GLU and TG	67963	320	0.075	0.192	0.98(0.84-1.14)	0.794
**IS**						
Reference	67930	410				
Higher GLU	67998	402	0.085	0.006	0.99(0.86-1.14)	0.900
Higher TG	67892	453	-0.014	0.185	1.17(1.02-1.34)	0.024
Higher GLU and TG	67852	431	0.075	0.192	1.13(0.99-1.30)	0.071

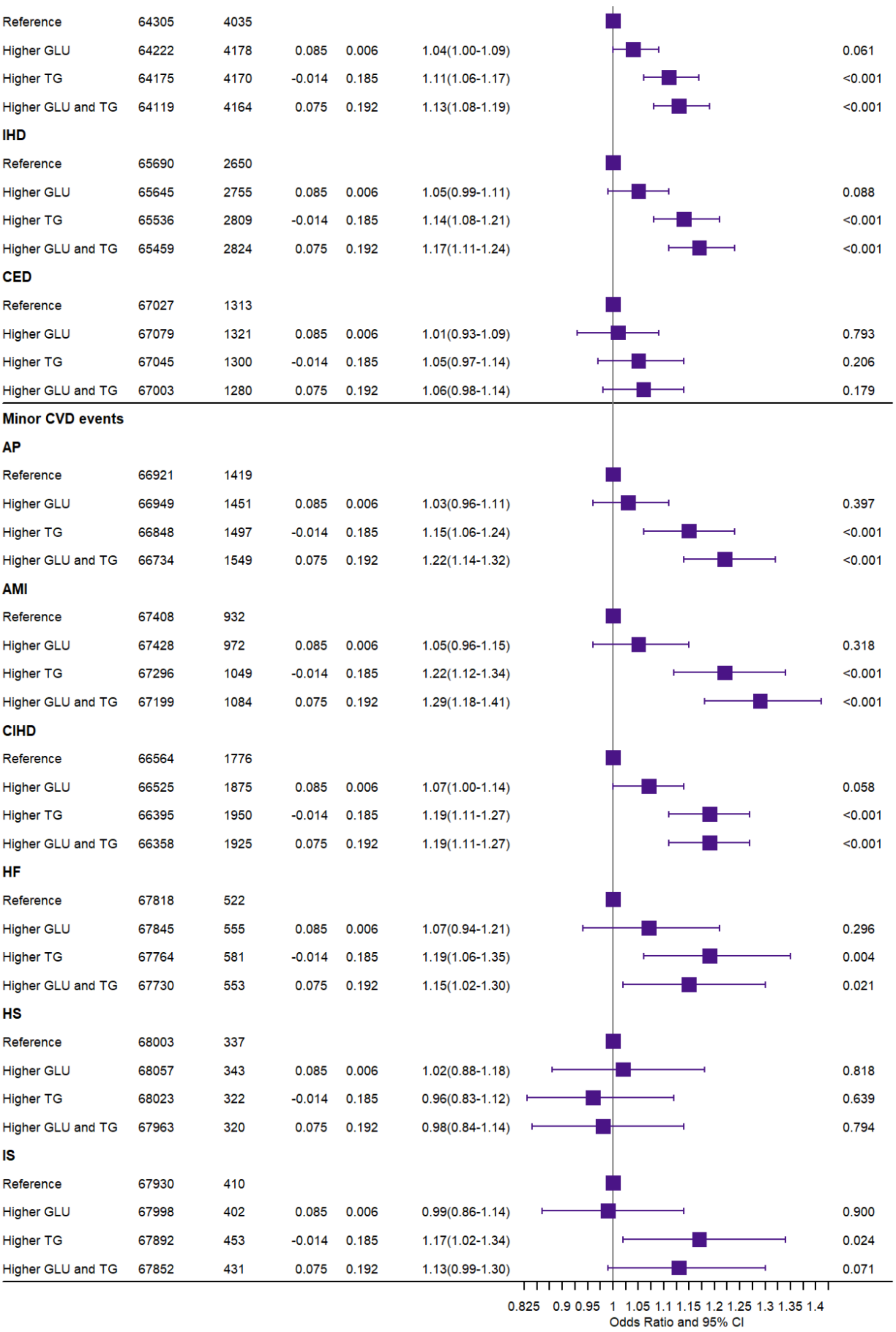

Figure S22. Association of combined exposure to GLU and TG in crude difference with the risk of CVD by weighted external reported SNPs in dataset A.

2×2 subgroup	No.control	No.case	ΔGLU	Δlog(TG)	Odds Ratio	P value
**Major CVD events**						
**Overall CVD**						
Reference	67839	3479				
Higher GLU	61667	3701	0.849	0.026	0.95(0.91-1.00)	0.065
Higher TG	61125	4340	0.007	0.819	1.20(1.14-1.26)	<0.001
Higher GLU and TG	66190	5027	0.930	0.823	1.16(1.10-1.21)	<0.001
**IHD**						
Reference	69129	2189				
Higher GLU	62966	2402	0.849	0.026	0.97(0.91-1.03)	0.313
Higher TG	62519	2946	0.007	0.819	1.26(1.19-1.34)	<0.001
Higher GLU and TG	67716	3501	0.930	0.823	1.24(1.17-1.32)	<0.001
**CED**						
Reference	70097	1221				
Higher GLU	64148	1220	0.849	0.026	0.93(0.86-1.01)	0.101
Higher TG	64133	1332	0.007	0.819	1.13(1.04-1.23)	0.005
Higher GLU and TG	69776	1441	0.930	0.823	1.04(0.95-1.13)	0.392
**Minor CVD events**						
**AP**						
Reference	70166	1152				
Higher GLU	64094	1274	0.849	0.026	0.97(0.90-1.06)	0.546
Higher TG	63928	1537	0.007	0.819	1.35(1.24-1.46)	<0.001
Higher GLU and TG	69264	1953	0.930	0.823	1.40(1.30-1.52)	<0.001
**AMI**						
Reference	70536	782				
Higher GLU	64495	873	0.849	0.026	0.99(0.90-1.10)	0.893
Higher TG	64366	1099	0.007	0.819	1.32(1.19-1.45)	<0.001
Higher GLU and TG	69934	1283	0.930	0.823	1.32(1.20-1.46)	<0.001
**CIHD**						
Reference	69831	1487				
Higher GLU	63732	1636	0.849	0.026	0.96(0.89-1.03)	0.249
Higher TG	63419	2046	0.007	0.819	1.24(1.16-1.33)	<0.001
Higher GLU and TG	68860	2357	0.930	0.823	1.18(1.10-1.27)	<0.001
**HF**						
Reference	70849	469				
Higher GLU	64869	499	0.849	0.026	0.89(0.78-1.01)	0.079
Higher TG	64916	549	0.007	0.819	0.97(0.85-1.10)	0.604
Higher GLU and TG	70523	694	0.930	0.823	1.00(0.88-1.13)	0.960
**HS**						
Reference	70983	335				
Higher GLU	65063	305	0.849	0.026	0.91(0.78-1.07)	0.252
Higher TG	65147	318	0.007	0.819	1.00(0.85-1.17)	0.966
Higher GLU and TG	70853	364	0.930	0.823	1.01(0.86-1.19)	0.901
**IS**						
Reference	70937	381				
Higher GLU	64996	372	0.849	0.026	0.87(0.76-1.01)	0.071
Higher TG	65004	461	0.007	0.819	1.12(0.97-1.29)	0.132
Higher GLU and TG	70735	482	0.930	0.823	0.97(0.84-1.12)	0.701

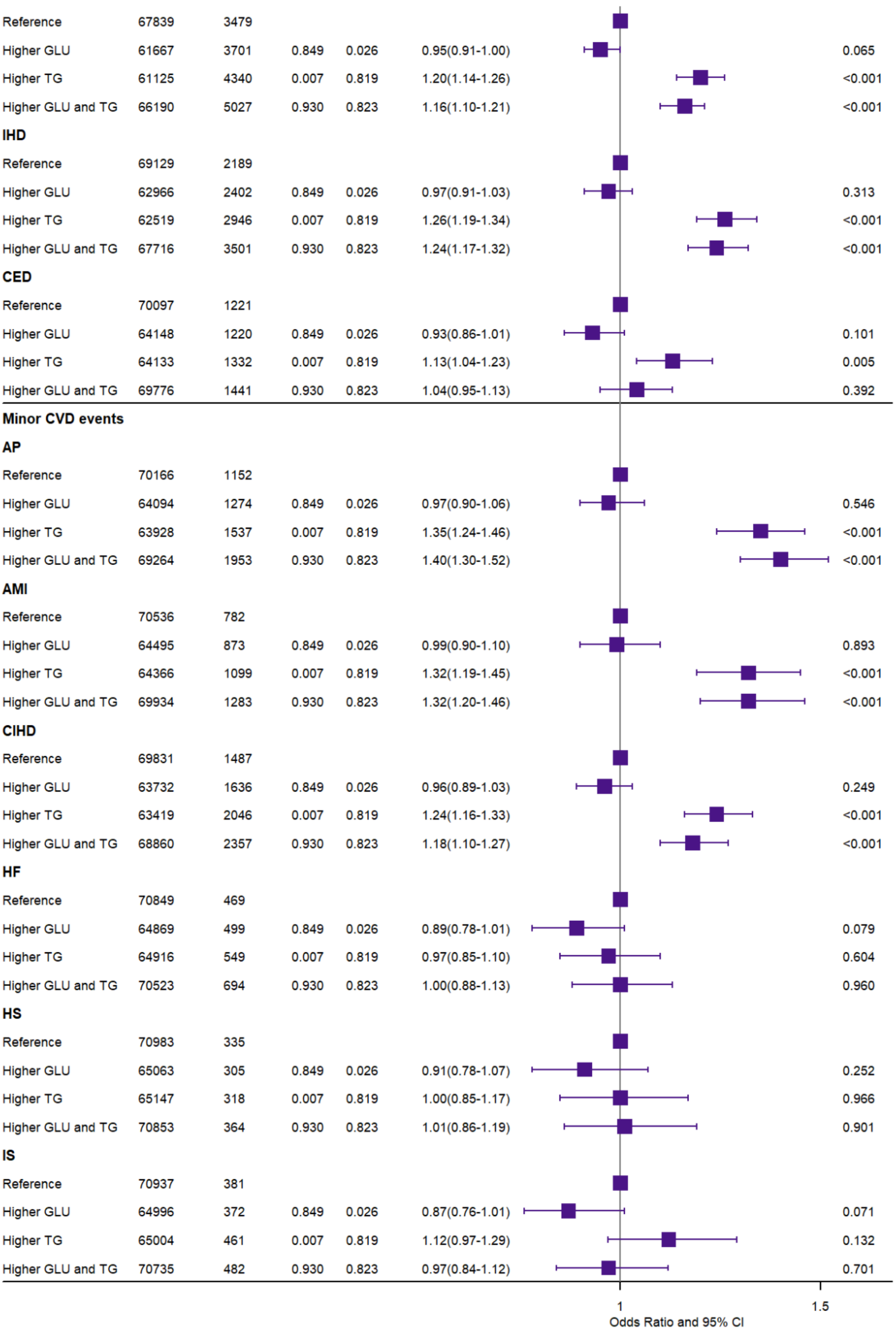

Figure S23. Association of combined exposure to GLU and TG in crude difference with the risk of CVD by observational phenotypes in dataset A.

TYG subgroup	No.control	No.case	ΔTyG	Odds Ratio	P value
**Major CVD events**					
**Overall CVD**					
Q1	64239	4103			
Q2	64287	4055	0.100	1.04(0.99-1.09)	0.128
Q3	64230	4112	0.180	1.07(1.02-1.12)	0.003
Q4	64065	4277	0.290	1.16(1.11-1.21)	<0.001
**IHD**					
Q1	65616	2726			
Q2	65679	2663	0.100	1.03(0.97-1.09)	0.332
Q3	65554	2788	0.180	1.10(1.04-1.16)	<0.001
Q4	65481	2861	0.290	1.17(1.11-1.24)	<0.001
**CED**					
Q1	67032	1310			
Q2	67038	1304	0.100	1.04(0.96-1.12)	0.333
Q3	67051	1291	0.180	1.04(0.96-1.13)	0.287
Q4	67033	1309	0.290	1.10(1.01-1.19)	0.022
**Minor CVD events**					
**AP**					
Q1	66884	1458			
Q2	66947	1395	0.100	1.01(0.94-1.09)	0.703
Q3	66850	1492	0.180	1.12(1.04-1.21)	0.003
Q4	66771	1571	0.290	1.22(1.14-1.32)	<0.001
**AMI**					
Q1	67356	986			
Q2	67348	994	0.100	1.07(0.98-1.17)	0.149
Q3	67316	1026	0.180	1.13(1.03-1.23)	0.010
Q4	67311	1031	0.290	1.17(1.07-1.28)	<0.001
**CIHD**					
Q1	66471	1871			
Q2	66572	1770	0.100	0.99(0.93-1.06)	0.797
Q3	66413	1929	0.180	1.11(1.03-1.18)	0.003
Q4	66386	1956	0.290	1.16(1.08-1.24)	<0.001
**HF**					
Q1	67772	570			
Q2	67786	556	0.100	1.02(0.91-1.15)	0.752
Q3	67802	540	0.180	1.00(0.88-1.12)	0.958
Q4	67797	545	0.290	1.05(0.93-1.19)	0.401
**HS**					
Q1	68010	332			
Q2	68008	334	0.100	1.02(0.88-1.19)	0.777
Q3	68036	306	0.180	0.94(0.81-1.10)	0.466
Q4	67992	350	0.290	1.09(0.93-1.27)	0.276
**IS**					
Q1	67937	405			
Q2	67911	431	0.100	1.11(0.97-1.28)	0.129
Q3	67902	440	0.180	1.14(1.00-1.31)	0.058
Q4	67922	420	0.290	1.14(0.99-1.31)	0.067

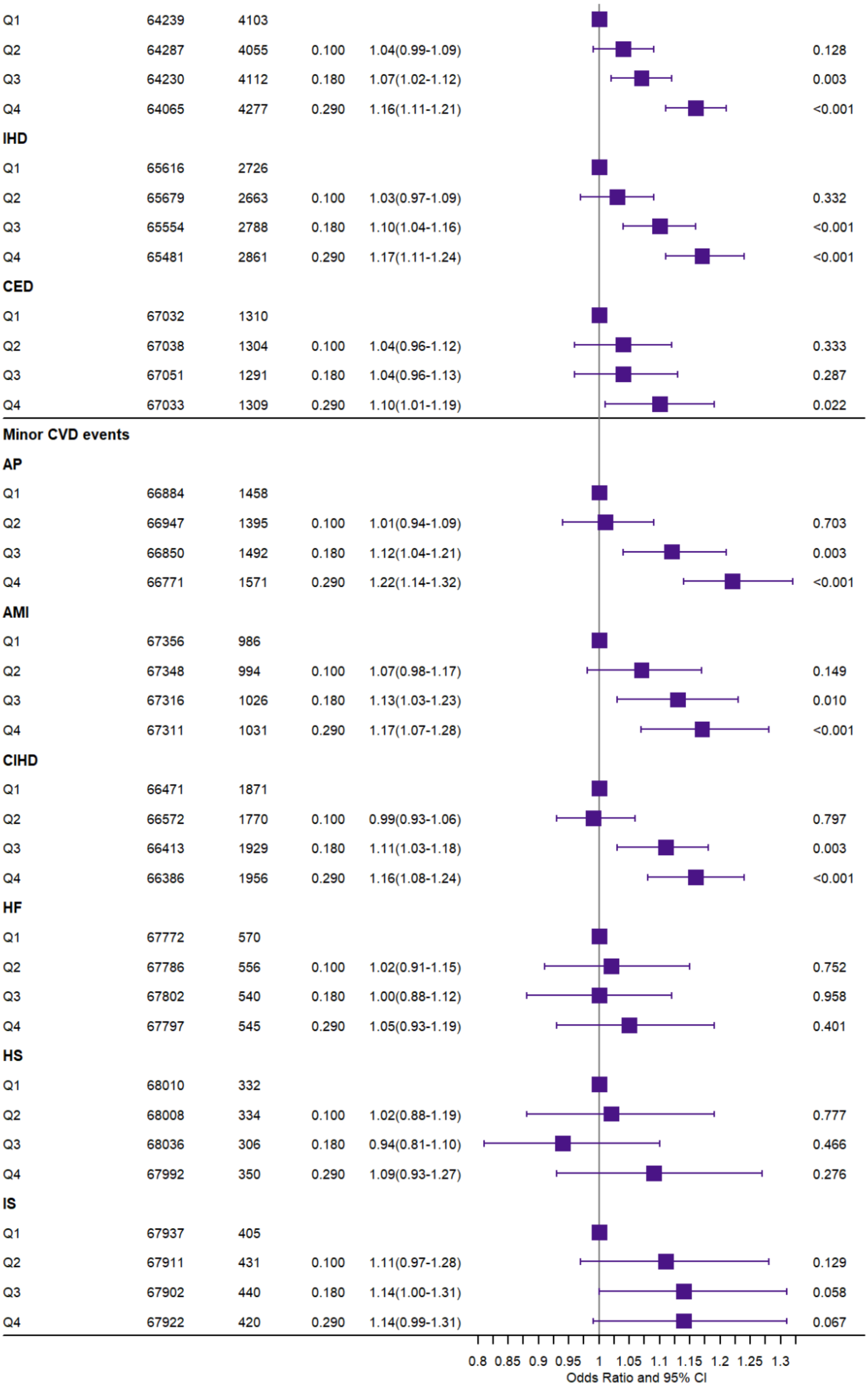

Figure S24. Association of TyG index with the risk of CVD by unweighted GRS in dataset A.

TYG subgroup	No.control	No.case	ΔTyG	Odds Ratio	P value
**Major CVD events**					
**Overall CVD**					
Q1	77090	9679			
Q2	76451	10318	0.130	1.10(1.07-1.14)	<0.001
Q3	76230	10539	0.230	1.15(1.12-1.19)	<0.001
Q4	75638	11131	0.370	1.24(1.20-1.28)	<0.001
**IHD**					
Q1	79524	7245			
Q2	78919	7850	0.130	1.12(1.08-1.16)	<0.001
Q3	78726	8043	0.230	1.18(1.14-1.22)	<0.001
Q4	78127	8642	0.370	1.28(1.24-1.33)	<0.001
**CED**					
Q1	84055	2714			
Q2	83918	2851	0.130	1.07(1.01-1.13)	0.015
Q3	83859	2910	0.230	1.10(1.05-1.17)	<0.001
Q4	83933	2836	0.370	1.08(1.02-1.14)	0.009
**Minor CVD events**					
**AP**					
Q1	82158	4611			
Q2	81753	5016	0.130	1.12(1.07-1.17)	<0.001
Q3	81608	5161	0.230	1.18(1.13-1.23)	<0.001
Q4	81093	5676	0.370	1.31(1.26-1.37)	<0.001
**AMI**					
Q1	83936	2833			
Q2	83678	3091	0.130	1.12(1.07-1.19)	<0.001
Q3	83564	3205	0.230	1.19(1.13-1.26)	<0.001
Q4	83255	3514	0.370	1.30(1.24-1.37)	<0.001
**CIHD**					
Q1	81144	5625			
Q2	80746	6023	0.130	1.10(1.06-1.15)	<0.001
Q3	80571	6198	0.230	1.16(1.12-1.21)	<0.001
Q4	80068	6701	0.370	1.27(1.22-1.32)	<0.001
**HF**					
Q1	85415	1354			
Q2	85312	1457	0.130	1.09(1.01-1.18)	0.023
Q3	85279	1490	0.230	1.14(1.06-1.23)	<0.001
Q4	85197	1572	0.370	1.20(1.11-1.29)	<0.001
**HS**					
Q1	86225	544			
Q2	86241	528	0.130	0.97(0.86-1.10)	0.658
Q3	86228	541	0.230	0.99(0.88-1.12)	0.891
Q4	86249	520	0.370	0.96(0.85-1.09)	0.532
**IS**					
Q1	85779	990			
Q2	85752	1017	0.130	1.05(0.96-1.15)	0.281
Q3	85639	1130	0.230	1.17(1.07-1.28)	<0.001
Q4	85668	1101	0.370	1.14(1.04-1.24)	0.003
**Basic disease**					
**DM**					
Q1	82465	4304			
Q2	82039	4730	0.130	1.15(1.09-1.20)	<0.001
Q3	81835	4934	0.230	1.25(1.19-1.30)	<0.001
Q4	81256	5513	0.370	1.42(1.36-1.48)	<0.001
**DLM**					
Q1	73426	13343			
Q2	71557	15212	0.130	1.22(1.19-1.26)	<0.001
Q3	70008	16761	0.230	1.43(1.40-1.47)	<0.001
Q4	67451	19318	0.370	1.78(1.74-1.83)	<0.001

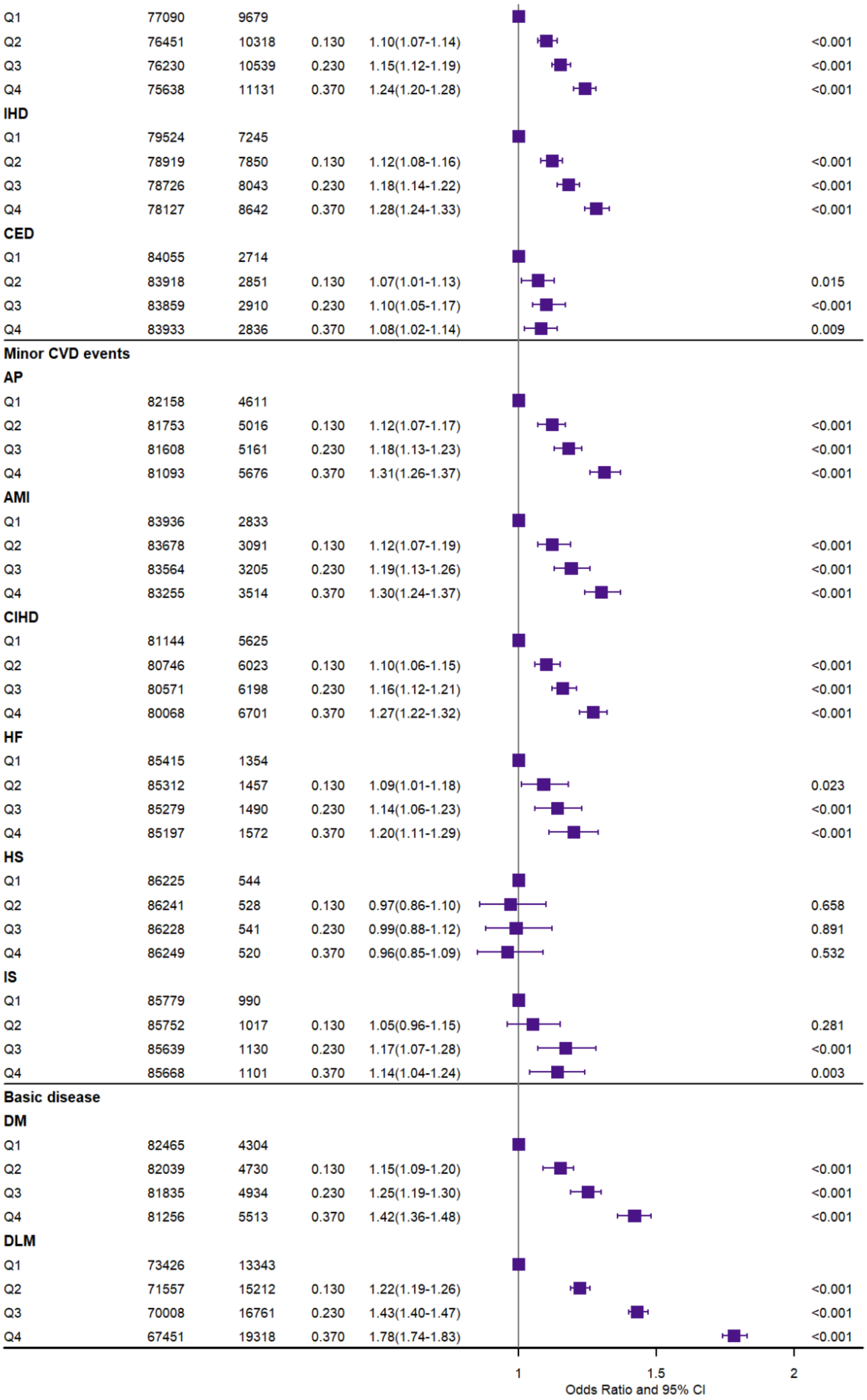

Figure S25. Association of TyG index with the risk of CVD by weighted GRS in dataset B.

TYG subgroup	No.control	No.case	ΔTyG	Odds Ratio	P value
**Major CVD events**					
**Overall CVD**					
Q1	64243	4099			
Q2	64316	4026	0.110	1.03(0.98-1.08)	0.268
Q3	64135	4207	0.190	1.11(1.06-1.16)	<0.001
Q4	64127	4215	0.310	1.15(1.10-1.21)	<0.001
**IHD**					
Q1	65650	2692			
Q2	65650	2692	0.110	1.05(0.99-1.11)	0.092
Q3	65576	2766	0.190	1.11(1.05-1.18)	<0.001
Q4	65454	2888	0.310	1.21(1.15-1.28)	<0.001
**CED**					
Q1	67020	1322			
Q2	67056	1286	0.110	1.01(0.94-1.10)	0.718
Q3	66988	1354	0.190	1.09(1.01-1.18)	0.028
Q4	67090	1252	0.310	1.04(0.96-1.13)	0.338
**Minor CVD events**					
**AP**					
Q1	66894	1448			
Q2	66947	1395	0.110	1.02(0.94-1.10)	0.626
Q3	66857	1485	0.190	1.13(1.05-1.22)	0.001
Q4	66754	1588	0.310	1.27(1.18-1.36)	<0.001
**AMI**					
Q1	67406	936			
Q2	67366	976	0.110	1.10(1.00-1.20)	0.044
Q3	67310	1032	0.190	1.20(1.09-1.31)	<0.001
Q4	67249	1093	0.310	1.33(1.22-1.46)	<0.001
**CIHD**					
Q1	66533	1809			
Q2	66489	1853	0.110	1.08(1.01-1.15)	0.035
Q3	66448	1894	0.190	1.13(1.06-1.21)	<0.001
Q4	66372	1970	0.310	1.23(1.15-1.31)	<0.001
**HF**					
Q1	67816	526			
Q2	67816	526	0.110	1.05(0.93-1.19)	0.449
Q3	67752	590	0.190	1.22(1.08-1.37)	0.001
Q4	67773	569	0.310	1.21(1.07-1.37)	0.002
**HS**					
Q1	68007	335			
Q2	68022	320	0.110	0.96(0.82-1.12)	0.600
Q3	67994	348	0.190	1.06(0.91-1.23)	0.476
Q4	68023	319	0.310	0.97(0.83-1.14)	0.727
**IS**					
Q1	67939	403			
Q2	67940	402	0.110	1.03(0.90-1.19)	0.647
Q3	67855	487	0.190	1.28(1.12-1.46)	<0.001
Q4	67938	404	0.310	1.09(0.95-1.25)	0.230

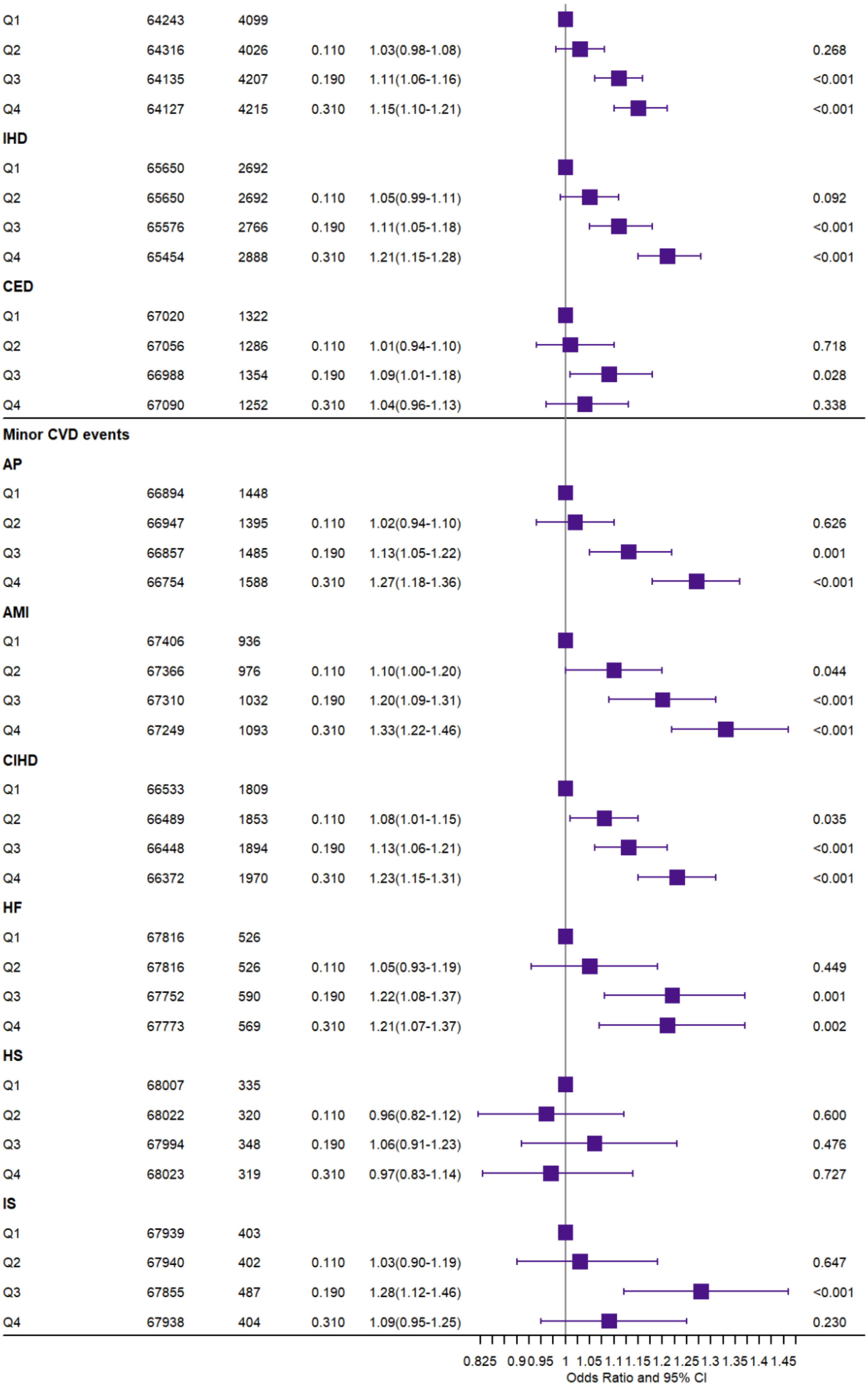

Figure S26. Association of TyG index with the risk of CVD by weighted external reported SNPs in dataset A.

TYG subgroup	No.control	No.case	ΔTyG	Odds Ratio	P value
**Major CVD events**					
**Overall CVD**					
Q1	65177	3165			
Q2	64375	3967	0.450	1.12(1.07-1.18)	<0.001
Q3	63851	4491	0.800	1.22(1.16-1.28)	<0.001
Q4	63418	4924	1.340	1.33(1.27-1.41)	<0.001
**IHD**					
Q1	66382	1960			
Q2	65744	2598	0.450	1.17(1.10-1.25)	<0.001
Q3	65273	3069	0.800	1.32(1.24-1.40)	<0.001
Q4	64931	3411	1.340	1.45(1.36-1.55)	<0.001
**CED**					
Q1	67212	1130			
Q2	67058	1284	0.450	1.06(0.97-1.15)	0.177
Q3	66975	1367	0.800	1.12(1.03-1.22)	0.008
Q4	66909	1433	1.340	1.20(1.10-1.31)	<0.001
**Minor CVD events**					
**AP**					
Q1	67314	1028			
Q2	66967	1375	0.450	1.22(1.12-1.33)	<0.001
Q3	66672	1670	0.800	1.45(1.34-1.58)	<0.001
Q4	66499	1843	1.340	1.66(1.52-1.81)	<0.001
**AMI**					
Q1	67645	697			
Q2	67408	934	0.450	1.24(1.12-1.37)	<0.001
Q3	67207	1135	0.800	1.45(1.31-1.61)	<0.001
Q4	67071	1271	1.340	1.58(1.43-1.75)	<0.001
**CIHD**					
Q1	66994	1348			
Q2	66582	1760	0.450	1.14(1.06-1.23)	<0.001
Q3	66270	2072	0.800	1.25(1.16-1.35)	<0.001
Q4	65996	2346	1.340	1.38(1.28-1.49)	<0.001
**HF**					
Q1	67880	462			
Q2	67832	510	0.450	0.88(0.77-1.00)	0.053
Q3	67749	593	0.800	0.91(0.80-1.04)	0.173
Q4	67696	646	1.340	0.95(0.83-1.09)	0.472
**HS**					
Q1	68032	310			
Q2	68015	327	0.450	1.01(0.86-1.19)	0.902
Q3	68025	317	0.800	0.98(0.83-1.16)	0.829
Q4	67974	368	1.340	1.15(0.97-1.37)	0.116
**IS**					
Q1	67988	354			
Q2	67960	382	0.450	0.93(0.80-1.08)	0.358
Q3	67870	472	0.800	1.09(0.94-1.26)	0.254
Q4	67854	488	1.340	1.10(0.94-1.28)	0.239

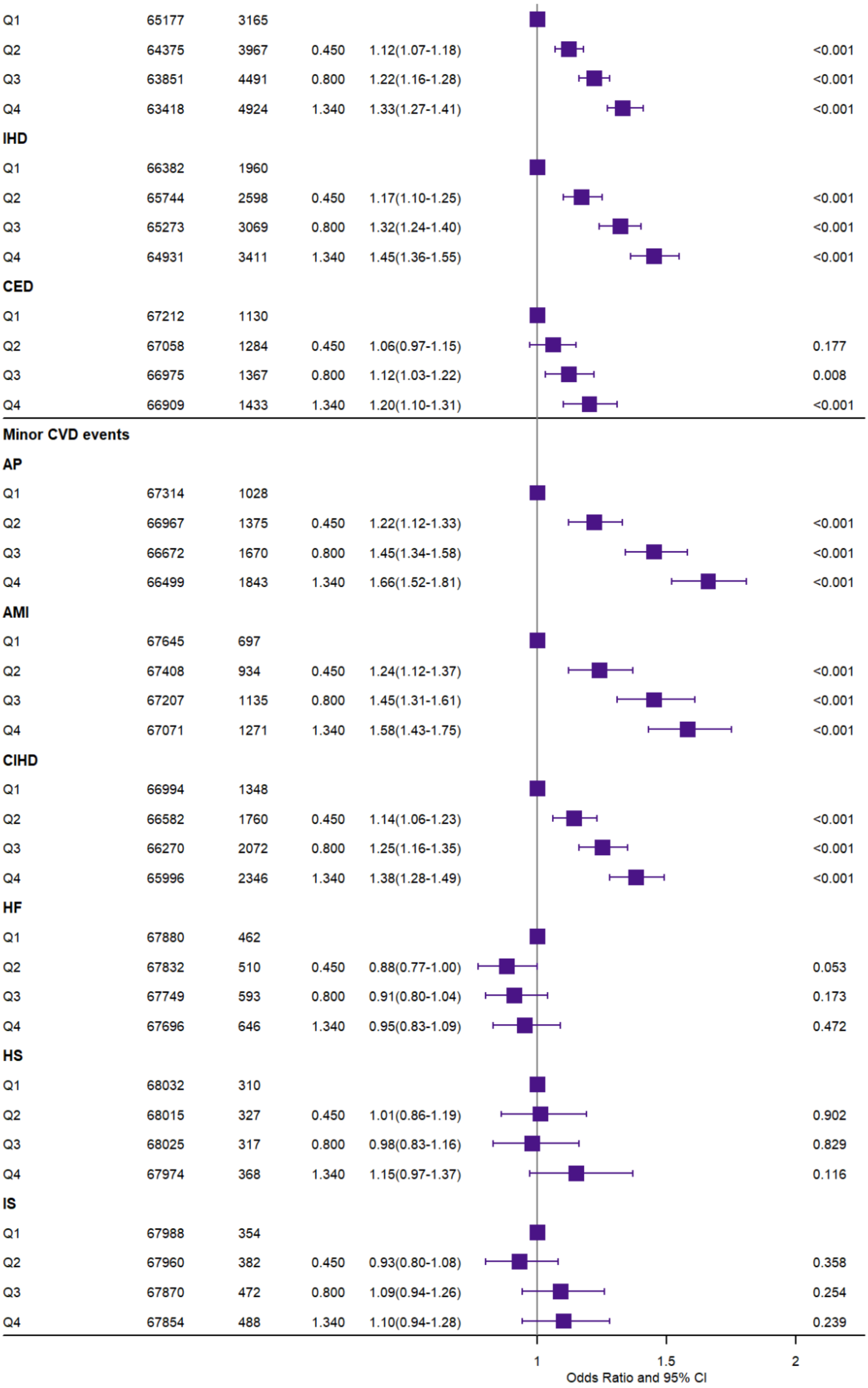

Figure S27. Association of observational TyG index with the risk of CVD in dataset A.

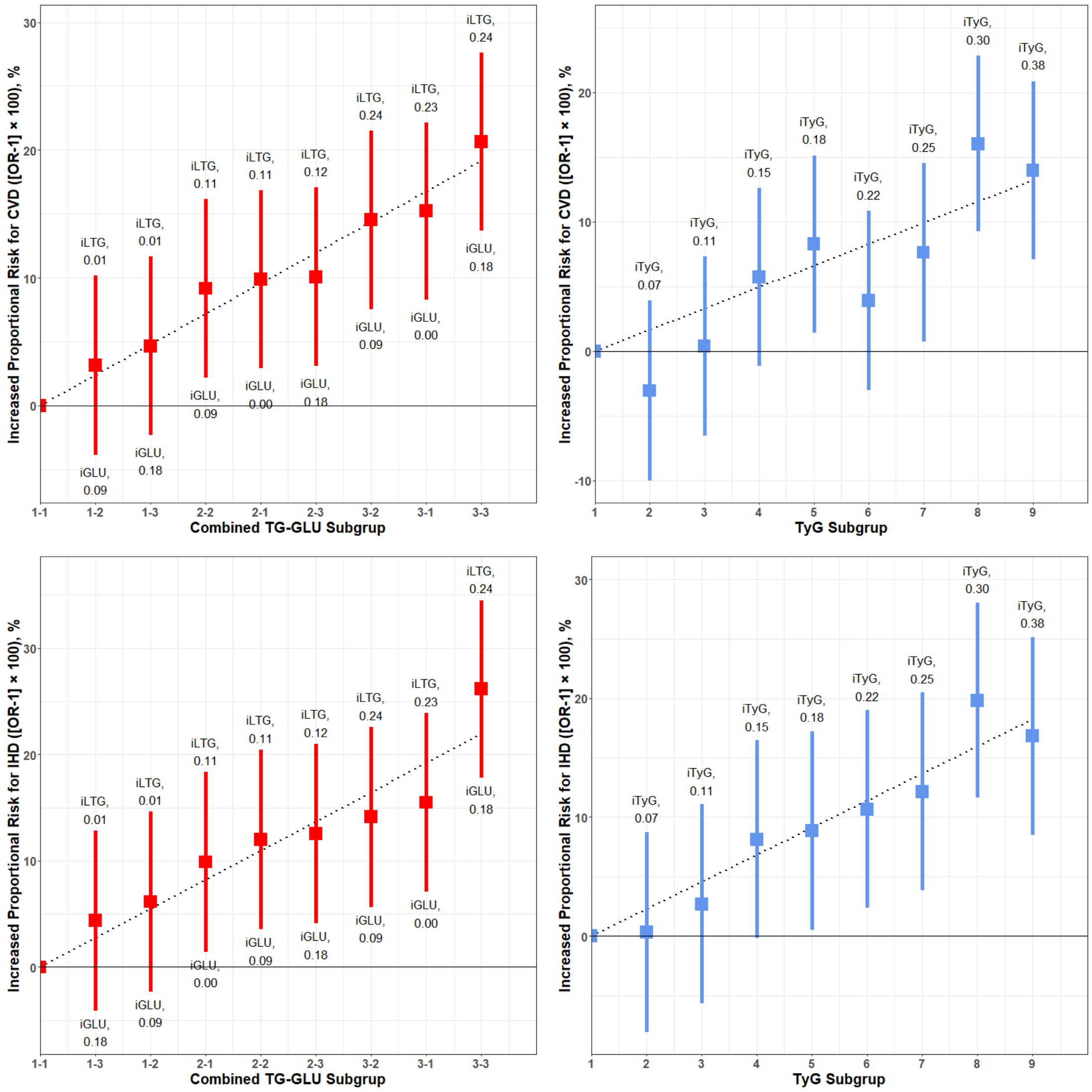

Figure S28. Dose-response associations of meta-regression for combined glucose and triglyceride on the risk of CVD and IHD by unweighted GRS in dataset A.

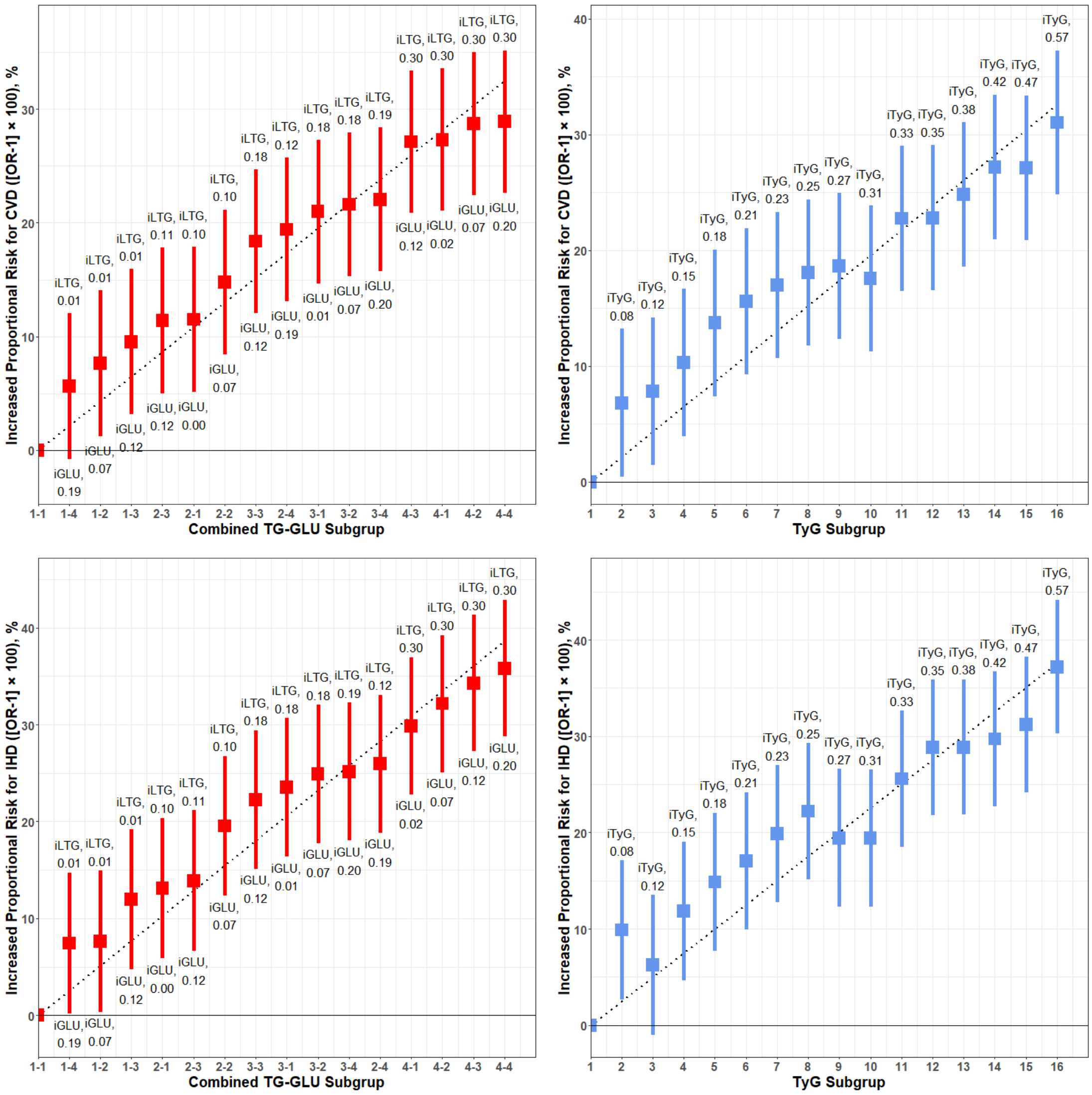

Figure S29. Dose-response associations of meta-regression for combined glucose and triglyceride on the risk of CVD and IHD by weighted GRS in dataset B.

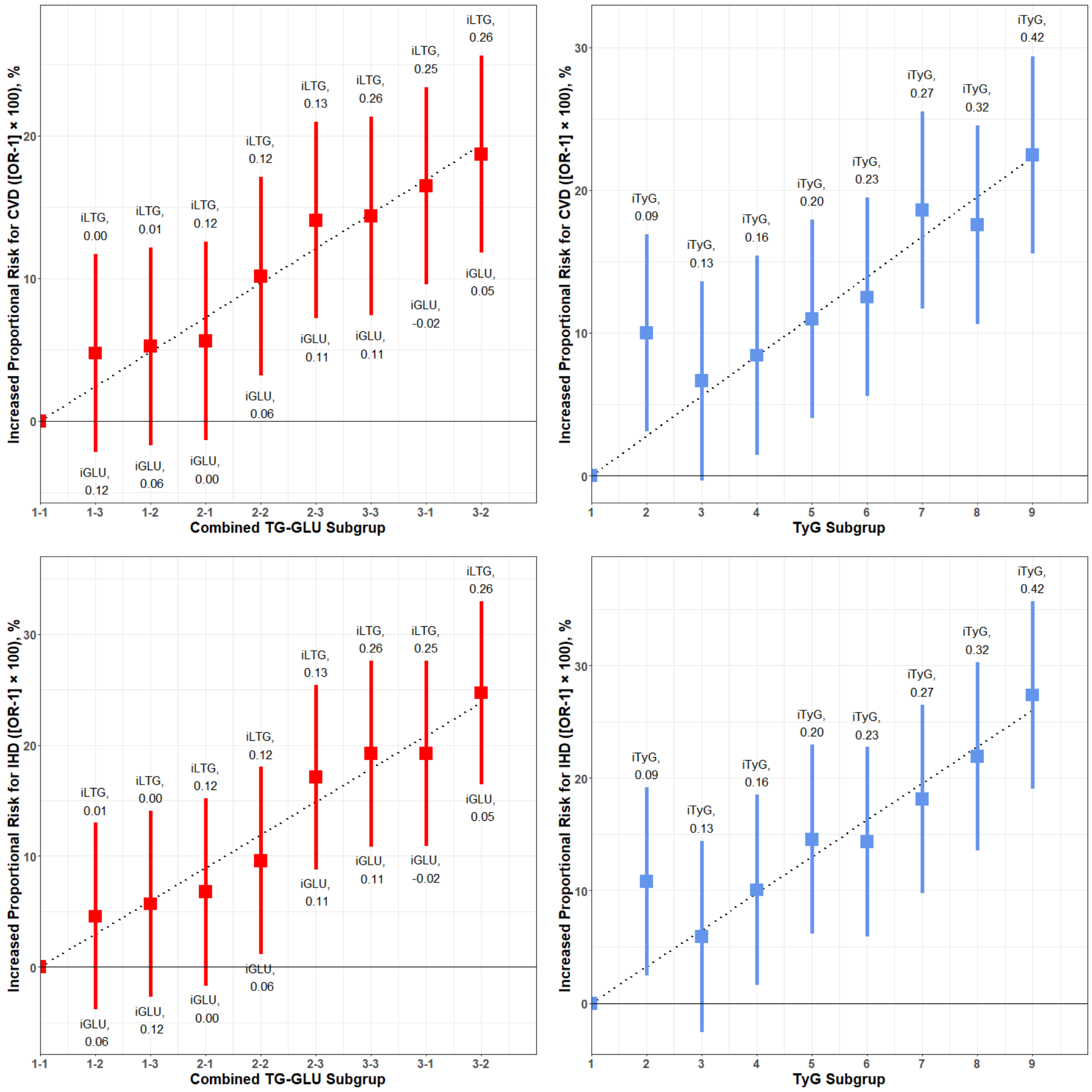

Figure S30. Dose-response associations of meta-regression for combined glucose and triglyceride on the risk of CVD and IHD by weighted external reported SNPs in dataset A.

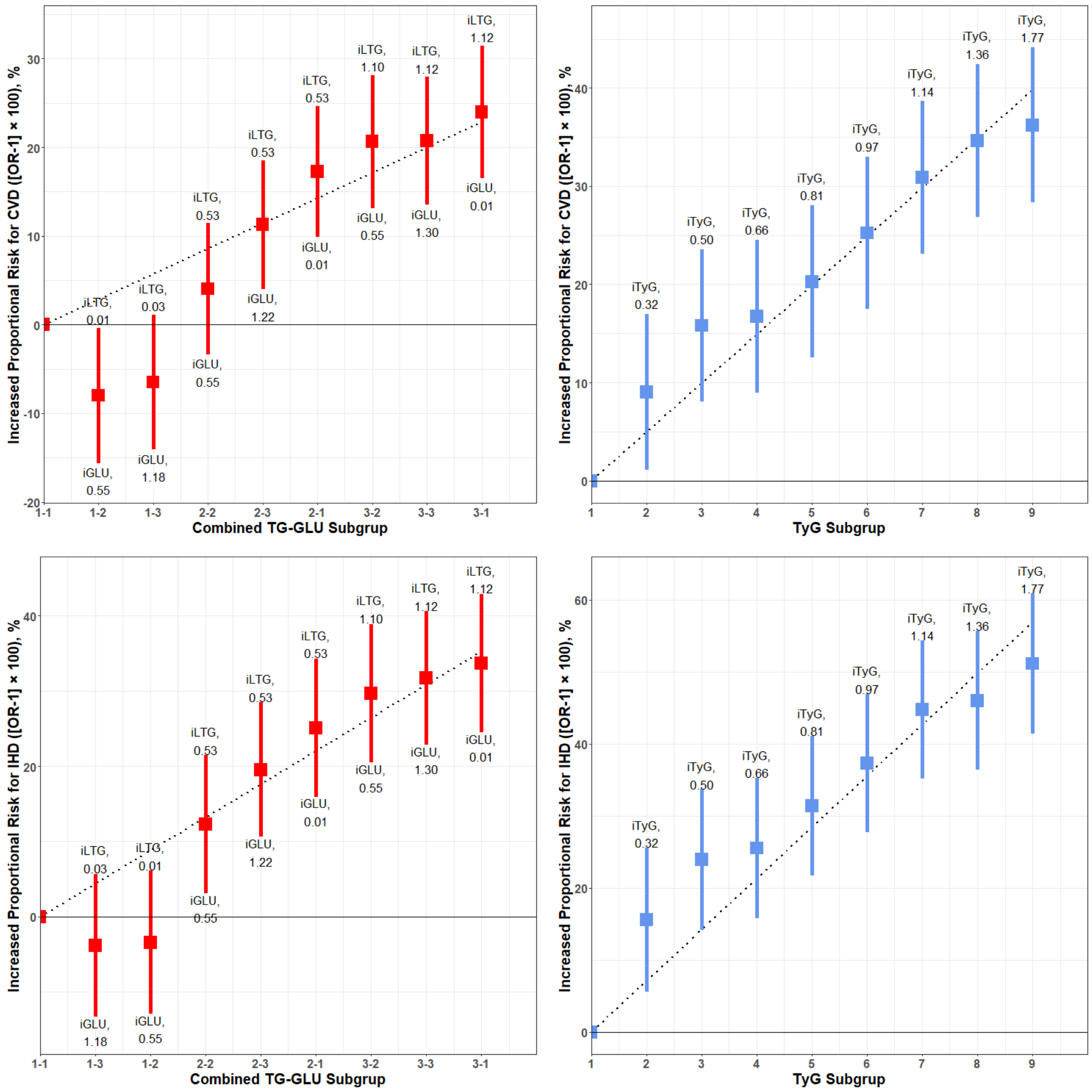

Figure S31. Dose-response associations of meta-regression for combined glucose and triglyceride on the risk of CVD and IHD by observational phenotypes in dataset A.

Subgroup	Control	Case	OR(95%CI)	P for interaction
**Combined GLU and TG**				
**Sex**				
Male	149846	6883	1.03(0.96-1.10)	>0.05
Female	106975	9664	1.11(1.04-1.17)	
**Age**				
≤median	139741	4745	1.14(1.05-1.24)	>0.05
>median	117080	11802	1.06(1.01-1.12)	
**BMI**				
≤median	129787	6533	1.08(1.01-1.16)	>0.05
>median	126407	9900	1.07(1.01-1.13)	
**Fasting time**				
≤median	144955	8540	1.08(1.01-1.15)	>0.05
>median	111857	8007	1.05(0.99-1.12)	
**HDL-C**				
≤median	126383	10416	1.04(0.98-1.10)	>0.05
>median	130384	6131	0.99(0.92-1.07)	
**LDL-C**				
≤median	126931	9608	1.17(1.10-1.24)	0.000
>median	129527	6916	0.99(0.93-1.06)	
**HbA1c**				
≤median	125362	6315	1.03(0.96-1.11)	>0.05
>median	119846	9516	1.03(0.97-1.10)	
**SBP**				
≤median	123129	6268	1.10(1.03-1.19)	>0.05
>median	116137	8960	1.05(0.99-1.12)	
**DBP**				
≤median	128232	7636	1.13(1.06-1.21)	0.014
>median	111038	7592	1.02(0.95-1.09)	
**Smoke status**				
No	148394	7644	1.12(1.05-1.19)	0.026
Yes	107690	8817	1.02(0.96-1.09)	
**TyG index**				
**Sex**				
Male	149846	6883	1.06(0.99-1.13)	>0.05
Female	106975	9664	1.08(1.02-1.14)	
**Age**				
≤median	139741	4745	1.12(1.03-1.21)	>0.05
>median	117080	11802	1.07(1.01-1.13)	
**BMI**				
≤median	129787	6533	1.12(1.05-1.21)	0.048
>median	126407	9900	1.03(0.97-1.09)	
**Fasting time**				
≤median	144955	8540	1.08(1.01-1.15)	>0.05
>median	111857	8007	1.05(0.98-1.11)	
**HDL-C**				
≤median	126383	10416	0.97(0.92-1.03)	>0.05
>median	130384	6131	0.99(0.92-1.07)	
**LDL-C**				
≤median	126931	9608	1.20(1.13-1.27)	0.000
>median	129527	6916	0.98(0.91-1.05)	
**HbA1c**				
≤median	125362	6315	1.13(1.05-1.22)	0.034
>median	119846	9516	1.03(0.97-1.09)	
**SBP**				
≤median	123129	6268	1.06(0.99-1.14)	>0.05
>median	116137	8960	1.07(1.01-1.14)	
**DBP**				
≤median	128232	7636	1.09(1.02-1.16)	>0.05
>median	111038	7592	1.05(0.98-1.12)	
**Smoke status**				
No	148394	7644	1.11(1.04-1.19)	0.040
Yes	107690	8817	1.02(0.96-1.09)	

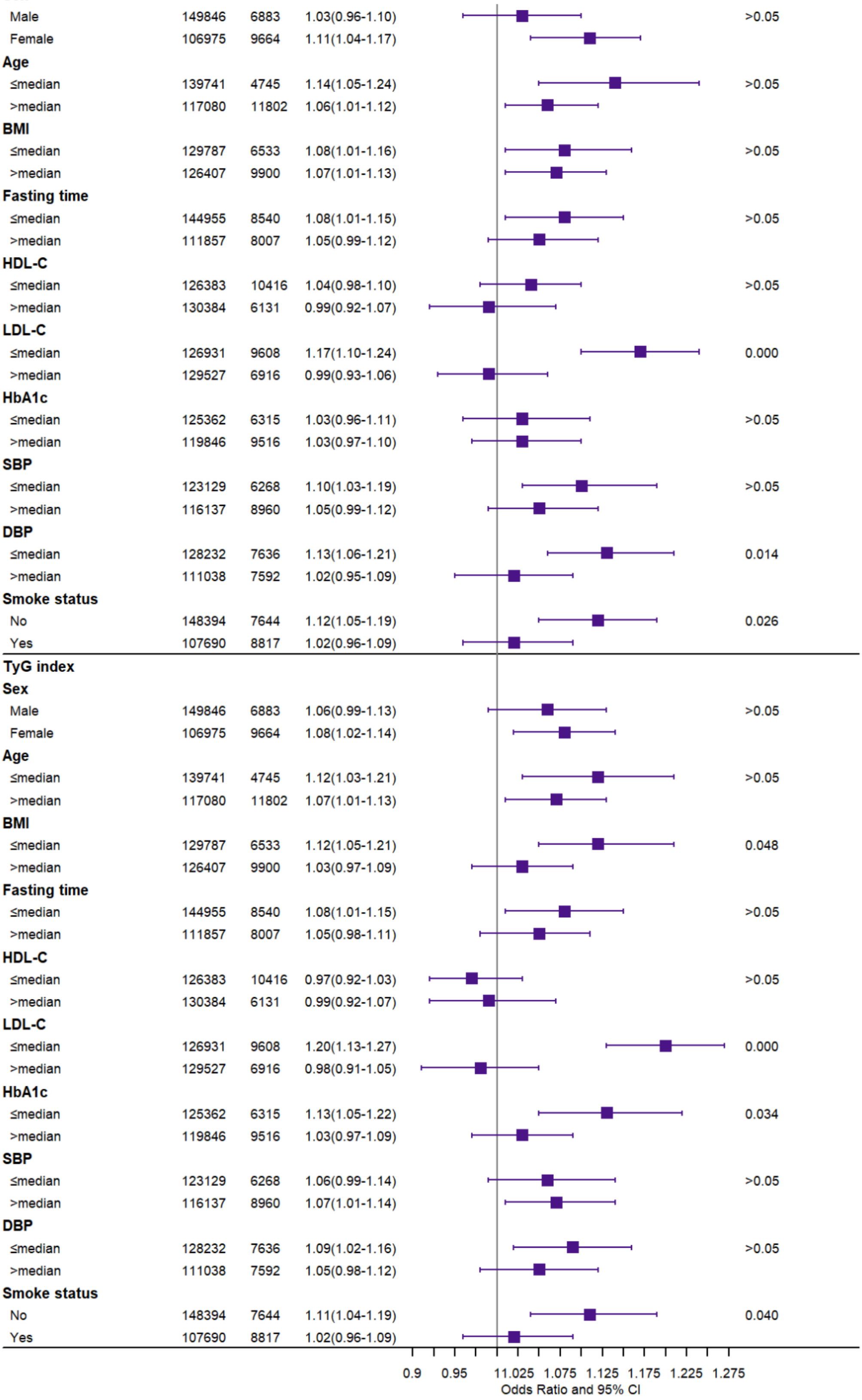

Figure S32. Association of combined GLU and TG with the risk of CVD in the subgroup in dataset A.

Disease	OR.GLU	P	OR.TG	P	OR.TyG	P
CVD	1.03(1.01-1.04)	0.001	1.06(1.05-1.08)	<0.001	1.06(1.05-1.08)	<0.001
IHD	1.04(1.02-1.06)	<0.001	1.08(1.06-1.10)	<0.001	1.08(1.06-1.10)	<0.001
CED	1.00(0.97-1.03)	0.969	1.04(1.01-1.07)	0.006	1.03(1.01-1.06)	0.019
AP	1.03(1.00-1.06)	0.026	1.09(1.06-1.12)	<0.001	1.10(1.07-1.13)	<0.001
AMI	1.05(1.02-1.09)	0.001	1.12(1.08-1.16)	<0.001	1.10(1.06-1.13)	<0.001
CIHD	1.05(1.02-1.07)	<0.001	1.09(1.06-1.11)	<0.001	1.08(1.05-1.10)	<0.001
HF	1.01(0.97-1.06)	0.543	1.04(1.00-1.09)	0.057	1.05(1.00-1.09)	0.042
HS	1.02(0.96-1.07)	0.585	1.02(0.96-1.07)	0.557	1.02(0.96-1.08)	0.511
IS	1.00(0.95-1.05)	0.881	1.07(1.02-1.12)	0.008	1.06(1.01-1.11)	0.024

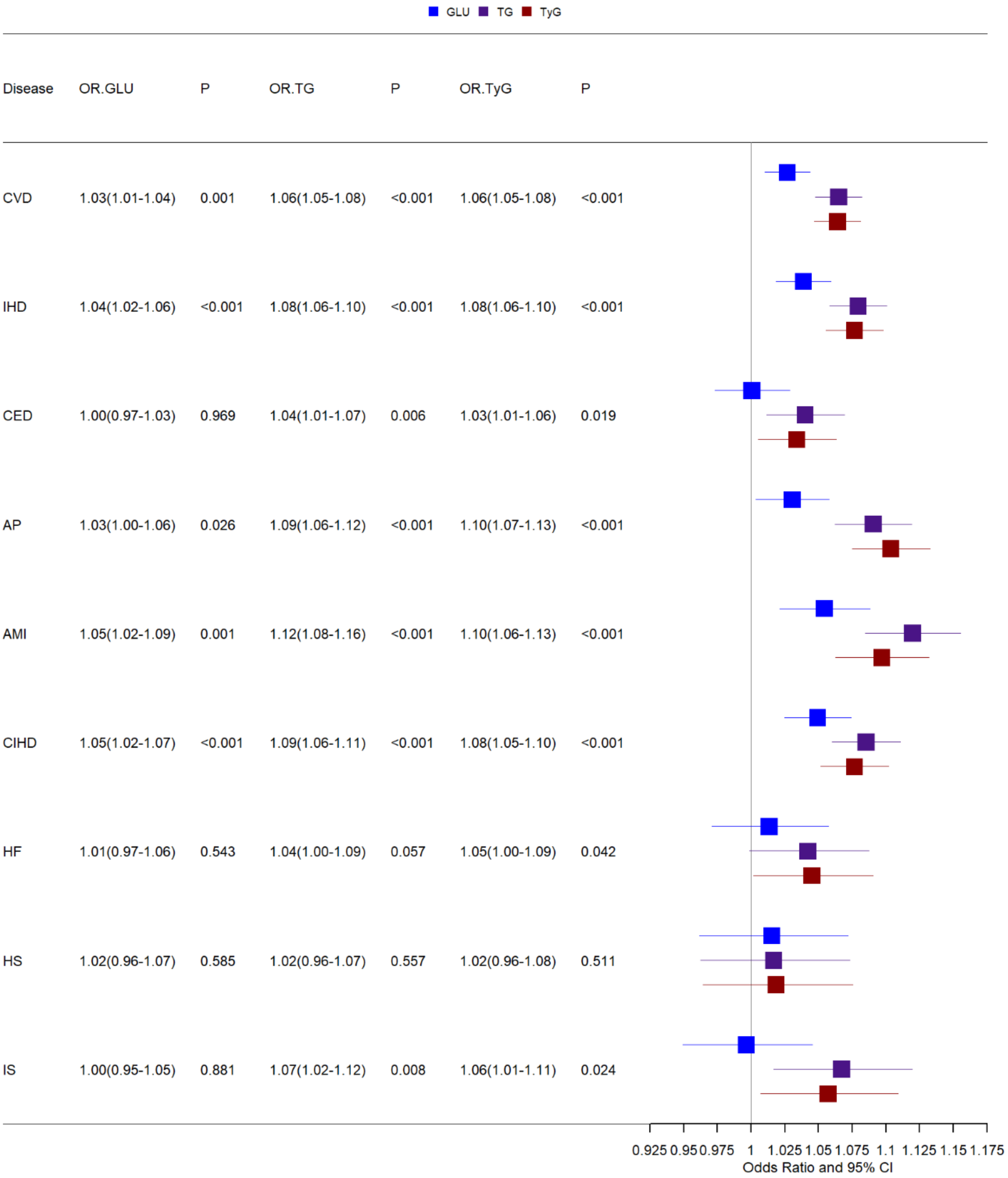

Figure S33. The effect of continuous GLU, TG, and TyG index on CVD and subtypes in dataset A.

Exposure	Outcome	OR(95%CI)	P value
GLU	Coronary heart disease	1.39(1.13-1.71)	<0.05
GLU	Myocardial infarction	1.28(1.03-1.58)	<0.05
GLU	Stroke	1.18(0.99-1.41)	0.066
GLU	Ischemic stroke	1.25(1.05-1.49)	<0.05
GLU	Cardioembolic stroke	1.15(0.82-1.62)	0.415
GLU	Large artery stroke	1.59(0.97-2.63)	0.068
GLU	Small vessel stroke	1.40(0.91-2.16)	0.126
TG	Coronary heart disease	1.93(1.66-2.24)	<0.05
TG	Myocardial infarction	1.87(1.61-2.18)	<0.05
TG	Stroke	1.09(0.97-1.23)	0.161
TG	Ischemic stroke	1.10(0.96-1.25)	0.181
TG	Cardioembolic stroke	1.05(0.85-1.31)	0.640
TG	Large artery stroke	1.54(1.11-2.13)	<0.05
TG	Small vessel stroke	1.21(0.89-1.64)	0.217
TYG	Coronary heart disease	1.80(1.46-2.21)	<0.05
TYG	Myocardial infarction	1.73(1.42-2.11)	<0.05
TYG	Stroke	1.12(0.99-1.26)	0.066
TYG	Ischemic stroke	1.11(0.98-1.27)	0.098
TYG	Cardioembolic stroke	0.97(0.79-1.19)	0.745
TYG	Large artery stroke	1.45(1.05-2.00)	<0.05
TYG	Small vessel stroke	1.34(1.01-1.78)	<0.05

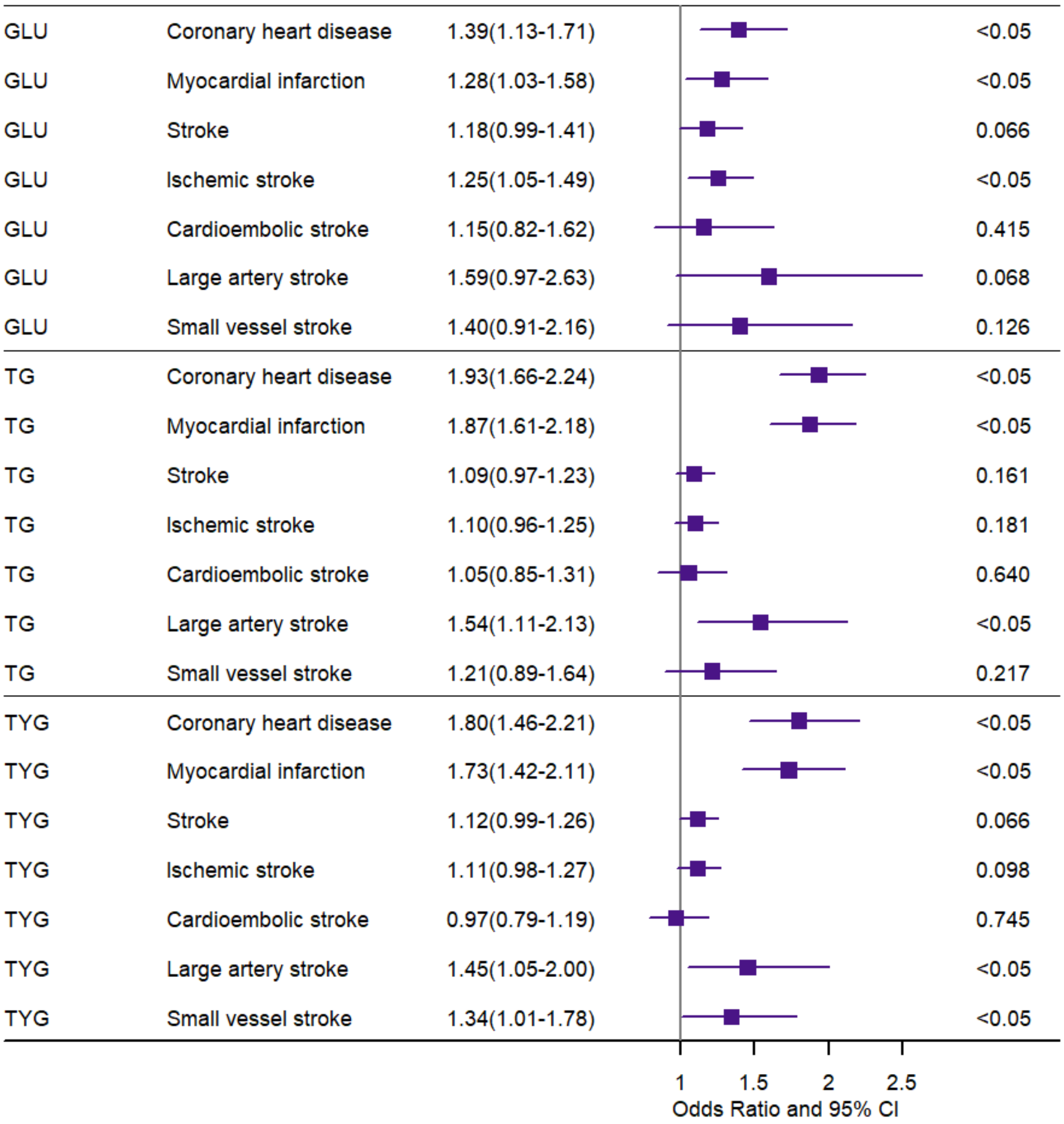

Figure S34. The association of GLU, TG, and TyG index with major CVD in external verification.

Exposure	Outcome	OR(95%CI)	P value
GLU	Coronary heart disease	1.45(1.17-1.82)	<0.05
GLU	Myocardial infarction	1.32(1.05-1.66)	<0.05
GLU	Stroke	1.14(0.95-1.38)	0.160
GLU	Ischemic stroke	1.21(1.01-1.45)	<0.05
GLU	Cardioembolic stroke	1.21(0.86-1.70)	0.264
GLU	Large artery stroke	1.50(0.88-2.54)	0.136
GLU	Small vessel stroke	1.25(0.80-1.95)	0.337
TG	Coronary heart disease	1.94(1.67-2.27)	<0.05
TG	Myocardial infarction	1.89(1.61-2.21)	<0.05
TG	Stroke	1.08(0.95-1.23)	0.226
TG	Ischemic stroke	1.07(0.94-1.23)	0.309
TG	Cardioembolic stroke	1.05(0.85-1.31)	0.652
TG	Large artery stroke	1.38(0.99-1.91)	0.056
TG	Small vessel stroke	1.22(0.89-1.67)	0.218
TYG	Coronary heart disease	1.79(1.45-2.21)	<0.05
TYG	Myocardial infarction	1.72(1.40-2.11)	<0.05
TYG	Stroke	1.10(0.97-1.24)	0.125
TYG	Ischemic stroke	1.09(0.96-1.25)	0.178
TYG	Cardioembolic stroke	0.97(0.80-1.18)	0.781
TYG	Large artery stroke	1.35(0.98-1.88)	0.070
TYG	Small vessel stroke	1.31(0.98-1.76)	0.068

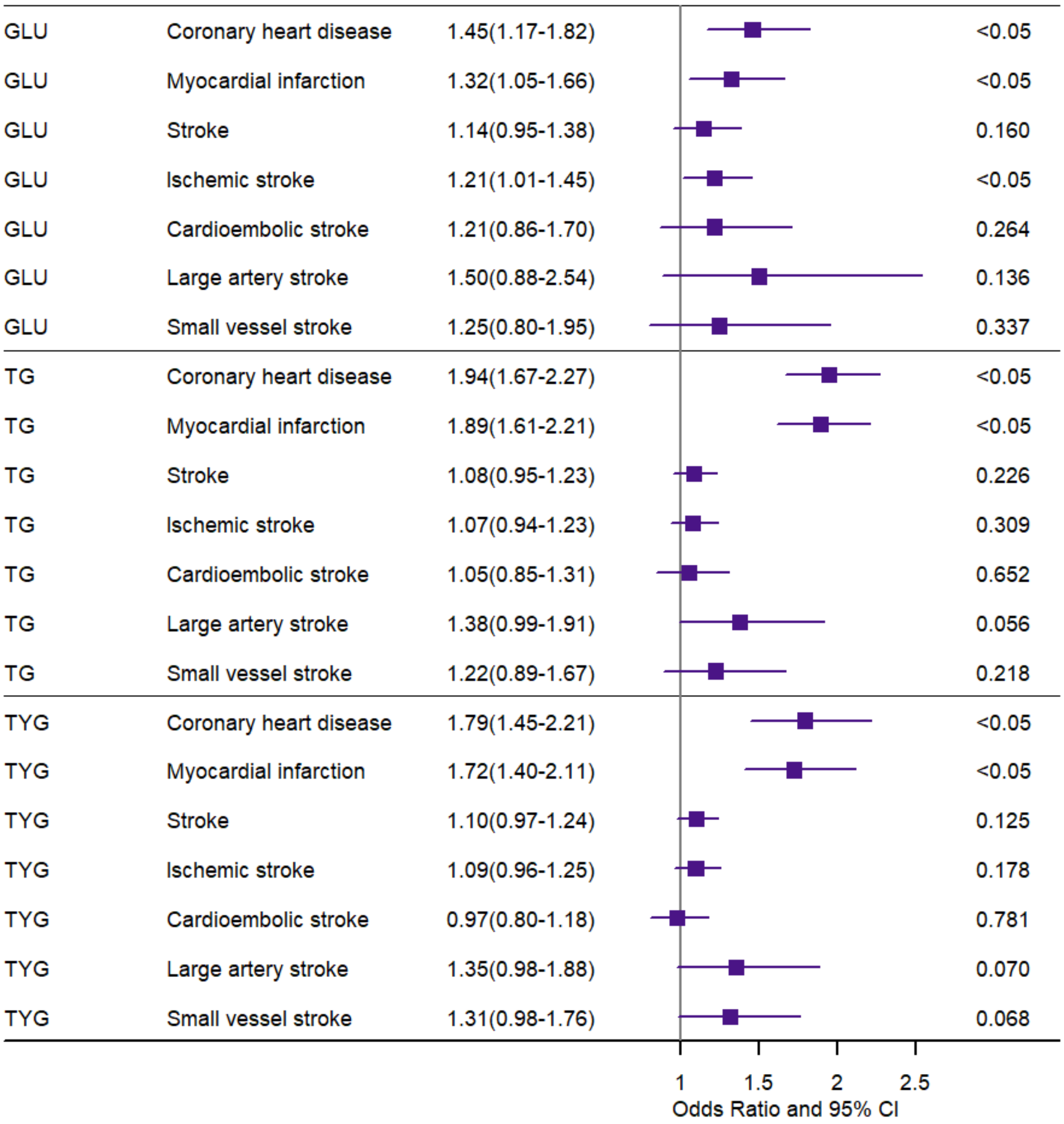

Figure S35. The association of GLU, TG, and TyG index with major CVD in external verification adjusted for BMI.
